# Biogeographic patterns of meio- and micro-eukaryotic communities in dam-induced river-reservoir systems

**DOI:** 10.1007/s00253-023-12993-4

**Published:** 2024-01-15

**Authors:** Huan Hu, Xing-Yi Wei, Li Liu, Yuan-Bo Wang, Ling-Kang Bu, Huang-Jie Jia, De-Sheng Pei

**Affiliations:** 1 Chongqing Jiaotong University, Chongqing, 400074 China; 2 Chongqing Institute of Green and Intelligent Technology, Chongqing School of University of Chinese Academy of Sciences, Chinese Academy of Sciences, Chongqing, 400714 China; 3 School of Public Health, Chongqing Medical University, Chongqing, 400016 China

**Keywords:** Community assembly, Meio- and micro-eukaryotic composition, Stochastic and deterministic processes, Species interactions, Three Gorges Reservoir

## Abstract

**Abstract:**

Although the Three Gorges Dam (TGD) is the world’s largest hydroelectric dam, little is known about the spatial–temporal patterns and community assembly mechanisms of meio- and micro-eukaryotes and its two subtaxa (zooplankton and zoobenthos). This knowledge gap is particularly evident across various habitats and during different water-level periods, primarily arising from the annual regular dam regulation. To address this inquiry, we employed mitochondrial cytochrome c oxidase I (COI) gene-based environmental DNA (eDNA) metabarcoding technology to systematically analyze the biogeographic pattern of the three communities within the Three Gorges Reservoir (TGR). Our findings reveal distinct spatiotemporal characteristics and complementary patterns in the distribution of meio- and micro-eukaryotes. The three communities showed similar biogeographic patterns and assembly processes. Notably, the diversity of these three taxa gradually decreased along the river. Their communities were less shaped by stochastic processes, which gradually decreased along the longitudinal riverine-transition-lacustrine gradient. Hence, deterministic factors, such as seasonality, environmental, and spatial variables, along with species interactions, likely play a pivotal role in shaping these communities. Environmental factors primarily drive seasonal variations in these communities, while hydrological conditions, represented as spatial distance, predominantly influence spatial variations. These three communities followed the distance-decay pattern. In winter, compared to summer, both the decay and species interrelationships are more pronounced. Taken together, this study offers fresh insights into the composition and diversity patterns of meio- and micro-eukaryotes at the spatial-temporal level. It also uncovers the mechanisms behind community assembly in various environmental niches within the dam-induced river-reservoir systems.

**Key points:**

• *Distribution and diversity of meio- and micro-eukaryotes exhibit distinct spatiotemporal patterns in the TGR.*

• *Contribution of stochastic processes in community assembly gradually decreases along the river.*

• *Deterministic factors and species interactions shape meio- and micro-eukaryotic community.*

**Graphical Abstract:**

**Figure:**
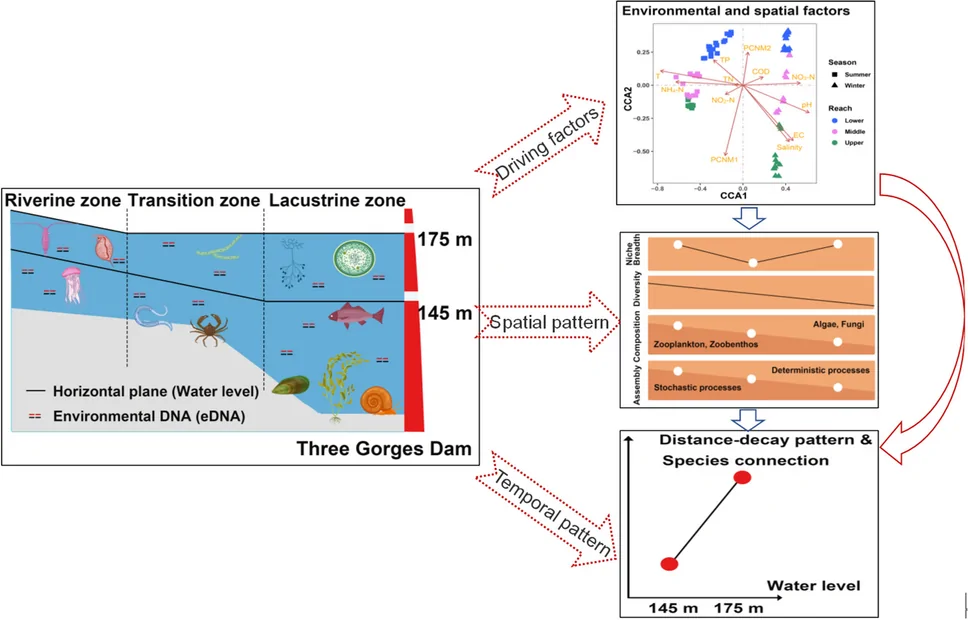

**Supplementary Information:**

The online version contains supplementary material available at 10.1007/s00253-023-12993-4.

## Introduction

The Three Gorges Reservoir (TGR) is one of the largest reservoirs for hydropower, stretching 670 km and covering 1084 km^2^ as it extends from Chongqing to the Three Gorges Dam (TGD) (Niu et al. 2019). The TGR serves as a vital source of drinking water for numerous residents (Zhou et al. 2011). Following the TGR project’s commencement in 2008, the reservoir’s water levels have displayed seasonal fluctuations, ranging from 145 m above sea level (ASL) during summer to 175 m ASL in winter (Ye et al. 2020). Consequently, the operation of the TGD results in distinct limnological characteristics in the main stem of the Yangtze River (Wetzel 2001). More precisely, the TGR transforms into a standard river-type reservoir, encompassing diverse aquatic environments (riverine, transition, and lacustrine zones) along its longitudinal axis (Qin et al. 2021), offering different environmental niches compared to natural basins or rivers. Furthermore, the regulation of water levels by the TGD during different seasons has a significant impact on the physiochemical properties of the sediments and water. This influence extends to factors such as nutrient levels, dissolved oxygen, sediment texture, temperature, pH, and light intensity. Consequently, the microbial community undergoes seasonal shifts throughout the year (Li et al. 2019).

Due to their small size and limited mobility, meio- and micro-eukaryotes in aquatic ecosystems are highly susceptible to habitat and environmental changes. Therefore, alterations in the aquatic environment will also impact the composition and diversity of their community (Mo et al. 2021). Zooplankton and zoobenthos are primary consumers in the food web, playing crucial ecological roles as key members of the meio- and micro-eukaryotes. Changes in their communities can also lead to significant impacts across all trophic levels (Que et al. 2021). Understanding the spatiotemporal patterns, assembly processes, and mechanisms of meio- and micro-eukaryotic communities in aquatic environments, along with their responses to environmental factors, is crucial for river health, water quality protection, and the development of environmental management strategies in artificial reservoirs (Chen et al. 2019; Zhang et al. 2021). Nevertheless, our comprehension of the ecological roles and environmental relevance of the meio- and micro-eukaryotic community, encompassing zooplankton and zoobenthos, within the TGR, remains limited. To address this gap, this study concentrates on investigating the meio- and micro-eukaryotic communities, along with their subcommunities: zooplankton and zoobenthos.

Traditionally, the biodiversity of meio- and micro-eukaryotes, such as zooplankton and zoobenthos, has been studied by identifying them through their morphological characteristics. However, many microorganisms are not identifiable via microscopy because of difficult morphological identification. This obstacle hinders our comprehension of meio- and micro-eukaryotic communities (Zou et al. 2021). Moreover, the traditional method of morphological identification is not only time-consuming and inefficient, but also demands trained taxonomists to carry it out (Qiu et al. 2022). Fortunately, environmental DNA (eDNA) sequencing techniques including high-throughput sequencing (HTS) and metabarcoding analysis of the mitochondrial cytochrome c oxidase I (COI) gene offer a promising solution for monitoring small eukaryotic organisms, such as zooplankton and zoobenthos (Ficetola et al. 2021). Metabarcoding also provides a semi-quantitative estimate of relative abundances, as the amount of DNA from a species in a sample correlates with the number of individuals of that species (Yang et al. 2017a). Despite a few limitations, eDNA-metabarcoding technology does provide solid information for ecological interpretations (Zou et al. 2021). For instance, the eDNA-metabarcoding technology can effectively clarify connections between community and environmental factors, unveiling interrelationships among species, with a particular focus on community assembly processes (Liu et al. 2022b; Mo et al. 2021; Zou et al. 2021).

An ecological community is a dynamic and intricate system influenced by deterministic and stochastic elements associated with ecological processes (Zhou and Ning 2017). In most cases, deterministic and stochastic processes work together to shape meio- and micro-eukaryotic communities (Chen et al. 2019). It is crucial to comprehend the roles of deterministic and stochastic processes in the assembly of meio- and micro-eukaryotic communities, which helps elucidate ecological processes and how communities respond to environmental changes (Zhang et al. 2021). The annual periodic water storage and the environmental pollution from the TGR have considerably disrupted the meio- and micro-eukaryotic communities, as evidenced by traditional morphological analysis (Wang et al. 2016, 2017; Zhou et al. 2009). However, the balance between deterministic and stochastic processes governing the assembly of these communities remains ambiguous. This is particularly true for the zooplankton and zoobenthos communities across three distinct habitats within the TGR.

This study employs eDNA-metabarcoding sequencing of the COI gene to track the biogeographic spatiotemporal dynamics of the meio- and micro-eukaryotic community. Additionally, it examines the two subcommunities, namely zooplankton and zoobenthos, along the TGR mainstream during high- and low-water level periods. This study aims to achieve three main objectives: (1) understand the spatial-temporal dynamics of composition and diversity within the three communities; (2) identify the dominant mechanism driving the assembly of these communities across various habitats and water-level periods—whether it is through stochastic or deterministic processes; and (3) investigate how specific deterministic factors (like seasonality, environmental, and spatial variables) and species interactions drive the variation in the three communities. To our knowledge, this study is the first to elucidate the assembly of meio- and micro-eukaryotic communities, specifically zooplankton and zoobenthos, in various habitats (riverine, transition, and lacustrine zones) of the TGR under high- and low-water levels.

## Materials and methods

### Study site and eDNA sampling

**Fig. 1:**
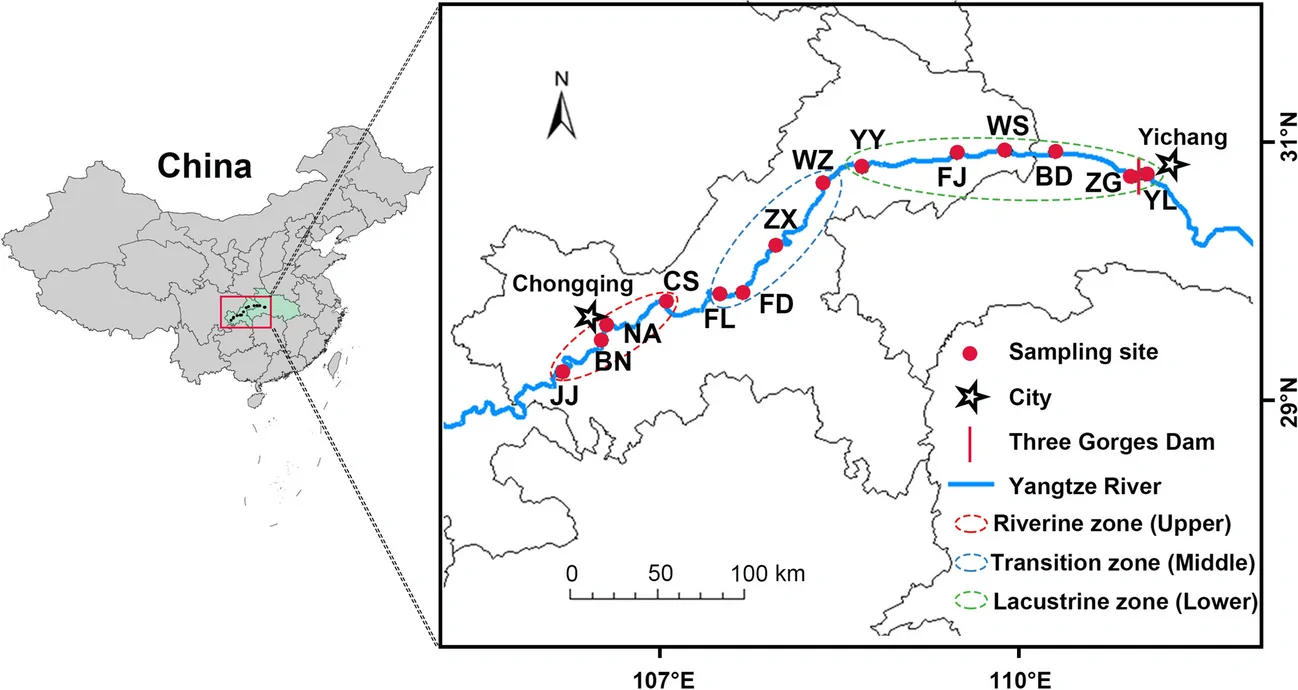
Map showing 14 sampling sites in the TGR, China. In 2021, a total of 84 surface water samples were collected during both summer and winter. These samples were gathered from three zones along the TGR: the riverine zone (upper reach, 4 sites), transition zone (middle reach, 4 sites), and lacustrine zone (lower reach, 6 sites). The sites include JJ (Jiangjin), BN (Banan), NA (Nanan), CS (Changshou), FL (Fuling), FD (Fengdu), ZX (Zhongxian), WZ (Wanzhou), YY (Yunyang), FJ (Fengjie), WS (Wushan), BD (Badong), ZG (Zigui), and YL (Yiling)

The studied aquatic ecosystem is located in the TGR, stretching from 106° to 111°50′ E and 29°16′ to 31°25′ N, covering the area from Chongqing to Yichang City, China, which includes 13 sites within the main stem of the Yangtze River and 1 site outside the TGD (Fig. 1). The 14 sites were situated across three distinct aquatic regions: the riverine zone (Jiangjin, Banan, Nanan, and Changshou), the transition zone (Fuling, Fengdu, Zhongxian, and Wanzhou), and the lacustrine zone (Fengjie, Yunyang, Wushan, Badong, Zigui, and Yiling) (Hu et al. 2023; Qin et al. 2021). In both the summer and winter of 2021, samples were collected from fourteen sites within the TGR at varying water levels: low (146.5 m) and high (172 m) impoundment. Each of the fourteen sites provided triplicate samples, collected 50–100 m apart, resulting in a total of 84 samples.

Each sampling event entailed the collection of 2 L of surface water (at a depth of 1 m) using a vertical water sampler. The collected water was temporarily kept on ice for less than 8 h before filtration. Afterward, 1 L of water was filtered through 0.22-μm pore-size polycarbonate membranes from Millipore (USA). These membranes were promptly transferred into 15-mL sterile centrifuge tubes and stored at -80 °C to facilitate eDNA extraction. The remaining 1 L of water was preserved in a clean and sterile glass container for subsequent physicochemical analysis.

### Physicochemical parameter analysis of water samples

Briefly, water temperature (T), pH, salinity, electrical conductivity (EC), and total dissolved solids (TDS) were measured in situ using professional pH/Conductivity/TDS Meters (EC500, ExStik^®^II, USA). Dissolved oxygen (DO) was also detected in situ using a multi-parameter water quality analyzer (Hydrolab DS5, Hach Company, Loveland, CO, USA). For each sample, we measured additional physicochemical parameters using 1 L of the remaining water. These parameters included chemical oxygen demand (COD), ammonia nitrogen (NH_4_-N), nitrate nitrogen (NO_3_-N), nitrite nitrogen (NO_2_-N), total nitrogen (TN), and total phosphorus (TP), following methods established in our previous studies (Liu et al. 2022a; Niu et al. 2019). Besides, the longitude and latitude of all 14 sampling sites were determined on-site using a portable global position system (GPS). The distance from each sampling location to the dam was calculated using an electronic map.

### eDNA extraction, PCR amplification, and sequencing

The eDNA extraction process involved 84 samples obtained from polycarbonate filter membranes. The E.Z.N.A^®^ Soil DNA Kit (USA) was employed for extraction, following the manufacturer’s instructions. Each eDNA sample was quantified using a QuantiFluor™-ST kit from Promega (China) and a Nanodrop 2000 spectrophotometer from Thermo (Waltham, MA, USA). Subsequently, the quality of the eDNA was assessed through 1% agarose gel electrophoresis.

To identify the meio- and micro-eukaryotic community and its subcommunities (zooplankton and zoobenthos), we amplified a partial gene fragment (313 bp) of the mitochondrial COI gene using the degenerate primers mlCOIintF and jgHCO2198 (Suter et al. 2021). The PCR reaction mixture from TransGen, China, consisted of 20 μL of TransStart FastPfu, including 4 μL of FastPfu Buffer (5 ×), 2 μL of dNTPs (2.5 mM), 0.8 μL each of forward and reverse primers (5 μM), 0.4 μL of FastPfu Polymerase (500 U), 11 μL of ddH_2_O, and 1 μL of template DNA (10 ng/μL). The PCR thermal program was executed as follows: initial denaturation at 95 °C for 5 min, followed by 35 cycles of denaturation at 95 °C for 30 s, annealing at 60 °C for 30 s, and extension at 72 °C for 45 s. A final extension step was performed at 72 °C for 10 min. Subsequently, the PCR products underwent purification, quantification, and assessment.

Lastly, the libraries were subjected to sequencing using the Illumina PE250 high-throughput platform (BIOZERON Biotechnology Co., Ltd., Shanghai, China) for COI amplicon, with a 2 × 250 bp paired-end sequencing approach. The raw sequence data have been deposited in the NCBI Sequence Read Archive (SRA) database under BioProject: PRJNA870158.

### Construction of reference database and bioinformatic analysis

The Mito-COI eukaryotic metadata comprises non-redundant mitochondrion and COI sequences retrieved from NCBI GenBank, based on keywords such as “Mitochondrion,” “Mitochondrial,” “COI,” and “COX1” (Yang et al. 2017c). Using TaxonKit, we obtained taxonomic information for each accession ID in the metadata. This process facilitated the final creation of the Mito-COI reference database (Hleap et al. 2021; Shen and Xiong 2019). The Mito-COI reference database, along with its construction method and code, has been deposited in GitHub (https://github.com/BANZUIGE/Mito-COI).

The initial amplicon sequences were processed using the standard Qiime2 pipeline (Bolyen et al. 2019), and the final abundance tables of amplicon sequence variants (ASVs) were generated using the DADA2 algorithm (Callahan et al. 2016). High-quality sequences were identified based on factors such as suitable length, high Phred-quality scores, absence of ambiguous bases, efficient trimming, and the removal of duplicate/chimeric sequences (Tee et al. 2021). Subsequently, the taxonomic classification of representative sequences from each ASV was conducted using Qiime2 and the Mito-COI reference database. To improve classification accuracy, sequences were annotated with decreasing similarity thresholds: starting at a higher threshold of 97% (Giebner et al. 2020), then at 95%, 90%, and finally at 80% for previously unclassified sequences (Mo et al. 2021; Stefanni et al. 2018; Yang et al. 2017b). The taxonomic assignments were modified based on the eukaryotic taxonomic reference to enhance the user-friendly and portability of the classification (Adl et al. 2019). The ASVs of zooplankton and zoobenthos were selected and organized into two subcommunities based on the experiment’s objective.

To reduce the impact of sequencing errors, we excluded ASVs of meio- and micro-eukaryotes found in fewer than 3 samples, or those with fewer than 30 sequences each. The ASV abundance data was then rarefied to match the sequencing depth of the samples with the lowest number of sequences. Rarefaction curves were generated based on the resampled ASV abundances, confirming the eligibility of sequencing. These normalized ASV abundance tables were subsequently used for all subsequent analyses.

### Diversity analysis

Alpha diversity (ASV richness and the Shannon index) was calculated using the vegan package in R. To assess differences in community diversity across different reaches, we employed analysis of variance (ANOVA). To compare alpha diversity and environmental variables between seasons, we conducted Mann-Whitney *U* tests. Furthermore, we calculated Spearman’s correlation coefficient to evaluate the relationship between environmental variables and alpha diversity of the meio- and micro-eukaryotes, along with its two subtaxa.

To analyze beta diversity among the three communities, we conducted non-metric multidimensional scaling (NMDS) ordination based on Bray-Curtis similarity. Differences in these communities between seasons or reaches were assessed through analysis of similarity (ANOSIM). Additionally, phylogenetic lineages were established using Bray-Curtis similarity across all samples, employing the phyloseq package. Visualization of these lineages was achieved using the ggtree package in R (Gao et al. 2021a).

### Neutral community model analysis

To assess the extent to which stochastic processes influence the three communities across two seasons (summer and winter) and three reaches (upper, middle, and lower), we employed the Sloan neutral community model (NCM) (Sloan et al. 2006). This model was implemented using an R code developed by a previous study (Chen et al. 2019). Nonlinear least-squares were applied to determine the best fit between the frequency of ASV occurrence and their relative abundance, and the overall fit to the neutral model was indicated by the parameter *R*^2^. When the *R*^2^ value is near 1, the community assembly is completely compatible with stochastic processes. We categorized all ASVs into three groups: those within the 95% confidence intervals of NCM predictions (neutral partition), those occurring more frequently than these intervals (above partition), and those occurring less frequently than these intervals (below partition).

### Factors affecting community variation

To enhance clarity and precision, the ASV data underwent an initial Hellinger transformation, while environmental variables were logarithmically transformed to ensure unbiased estimations. Spatial vectors representing the sampling sites were generated using principal coordinates of neighboring matrix (PCNM) analysis through the vegan package, based on geographical coordinates. The selection of environmental and spatial variables (represented by PCNM vectors) was carried out using forward selection via the “ordiR2step” function. Only impactful factors with significance on the community were retained for subsequent analysis (Chen et al. 2019).

Variation partitioning analysis (VPA) with adjusted *R*^2^ coefficients was performed to evaluate the relative contributions of seasonal, environmental, and spatial factors in shaping community composition during both single or two seasons. Linear least-squares regression was performed to establish the distance-decay relationship between Bray-Curtis similarity among the three communities and the geographical distance of sampling sites during two seasons (Zou et al. 2021). The connections between the three communities’ composition and environmental and spatial variables (represented by PCNM vectors) were then established through canonical correspondence analysis (CCA).

Levins’ habitat niche breadth index (*B*) was calculated for the three communities across different reaches and seasons using the “niche.width” function in the spaa package. The community-level *B* value (*Bcom*) represents the average *B* value of all ASVs within a specific community (Mo et al. 2021).

### Co-occurrence network analysis

Co-occurrence network analysis was performed on meio- and micro-eukaryotes during two seasons. This analysis was carried out using the SparCC package at the ASV level, specifically focusing on ASVs present in over 50% of samples and with a relative abundance of ≥ 0.1% to reduce dataset complexity. Adjusted *p* values were obtained using the Benjamini-Hochberg (BH) method for multiple comparison correction. Valid co-occurrence events were indicated by statistically strong correlations (Spearman’s correlation coefficient |*r*| > 0.7 with *p* < 0.01) (Zou et al. 2021). Furthermore, we performed topological and modular analyses using the igraph package and visualized the network graph with Gephi (Mo et al. 2021).

All computations and plot generation were performed using R package v4.1.2 (Team RC 2013).

## Results

### Comparison of environmental variables between summer and winter

The environmental variables are presented in Table S1. Among the 12 variables, only COD and TN did not exhibit a significant difference between the two seasons (Table S2). In general, during the low-water level of summer, the mean values of T, NO_2_-N, NH_4_-N, and TP were notably higher compared to the high-water level of winter. Conversely, pH, DO, EC, salinity, TDS, and NO_3_-N exhibited higher values in winter than in summer.

### Composition and spatiotemporal patterns of the meio- and micro-eukaryotes

In our Mito-COI reference database sourced from the NCBI Genebank (Fig. S1a), there were over 5,380,000 sequences. Through Illumina sequencing, 52,214,486 high-quality sequences of the COI gene were obtained from 84 samples. These samples yielded sequence counts ranging from 386,622 to 749,246 (Table S1). After denoising, we identified 17,667 qualified ASVs. These representative sequences were annotated under different similarity thresholds at the species level: 3394 ASVs at 97%, 1985 ASVs at 95%, 4346 ASVs at 90%, and 3768 ASVs at 80% (Table S3). These encompassed 3450 ASVs from zooplankton, 1501 ASVs from zoobenthos, 5650 ASVs from algae, and 2259 ASVs from fungi (Fig. S1b). For further analysis, ASV abundance data were normalized to the sequencing depth of the sample with the fewest sequences, which was 369,114. The rarefaction curves reached a plateau, indicating sufficient sequencing depth for the resampled dataset of meio- and micro-eukaryotes (Fig. S2).

**Fig. 2:**
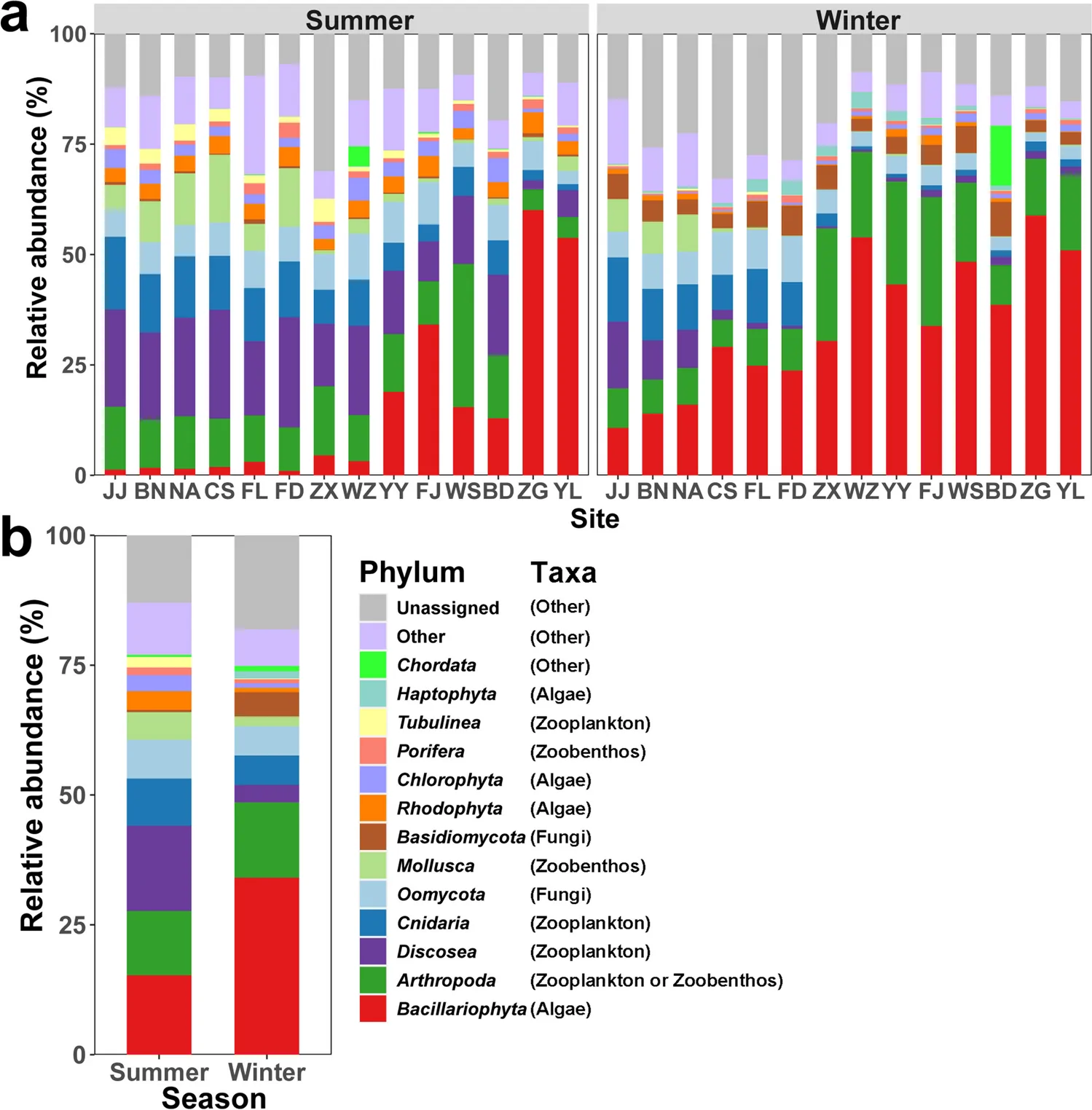
Composition dynamics of meio- and micro-eukaryotic communities in the TGR. **a** Relative abundances of meio- and micro-eukaryotes at each site along the TGR in summer and winter. **b** Overall relative abundances of meio- and micro-eukaryotes across two seasons. Other, includes merged phyla with less than 1% abundance. Unassigned, refers to unidentified ASVs

In the TGR, the distribution of meio- and micro-eukaryotes exhibits distinct spatial dynamics at the phylum level during summer and winter (Fig. 2a). For instance, during summer, there is a significant decline in the average relative abundance of *Arthropoda* (zooplankton or zoobenthos, 13.7% vs. 4.7%), *Discosea* (zooplankton, 18.5% vs. 4.1%), *Cnidaria* (zooplankton, 10.3% vs. 1.8%), and *Mollusca* (zoobenthos, 7.5% vs. 1.5%) near the TGD (Zigui and Yiling). Conversely, during winter, the average relative abundance of *Arthropoda* (zooplankton or zoobenthos, 8.1% vs. 19.3%) is significantly lower in the first six sampling sites (Jiangjin, Banan, Nanan, Changshou, Fuling, and Fengdu) compared to the last eight sampling sites (Zhongxian, Wanzhou, Fengjie, Yunyang, Wushan, Badong, Zigui, and Yiling) within the reservoir. Furthermore, during winter, the average relative abundance of *Discosea* (zooplankton, 8.7% vs. 1.6%), *Cnidaria* (zooplankton, 11.3% vs. 1.4%), *Oomycota* (fungi, 8.0% vs. 3.4%), and *Mollusca* (zoobenthos, 7.7% vs. 0.2%) in the upstream reservoir area is significantly higher than that in the downstream reservoir area. Notably, the relative abundance of *Bacillariophyta* (algae) follows an increasing trend along the river and reaches its peak (50.9 ~ 60.1%) near the TGD (Zigui and Yiling) in both seasons.

Within the TGR, the distribution of meio- and micro-eukaryotes exhibits a clear temporal dynamic pattern at the phylum level (Fig. 2b). Notably, the average relative abundance of *Bacillariophyta* (algae) increased significantly from 15.2 to 34%, and *Basidiomycota* (fungi) from 0.4 to 4.7%, as the seasons transitioned from summer to winter. Conversely, *Discosea* (zooplankton) declined notably from 16.4 to 3.4%, *Cnidaria* (zooplankton) decreased from 9.1 to 5.6%, and *Mollusca* (zoobenthos) decreased from 5.3 to 1.9% during the same period.

### Diversity of meio- and micro-eukaryotes and correlation with environmental variables

**Fig. 3:**
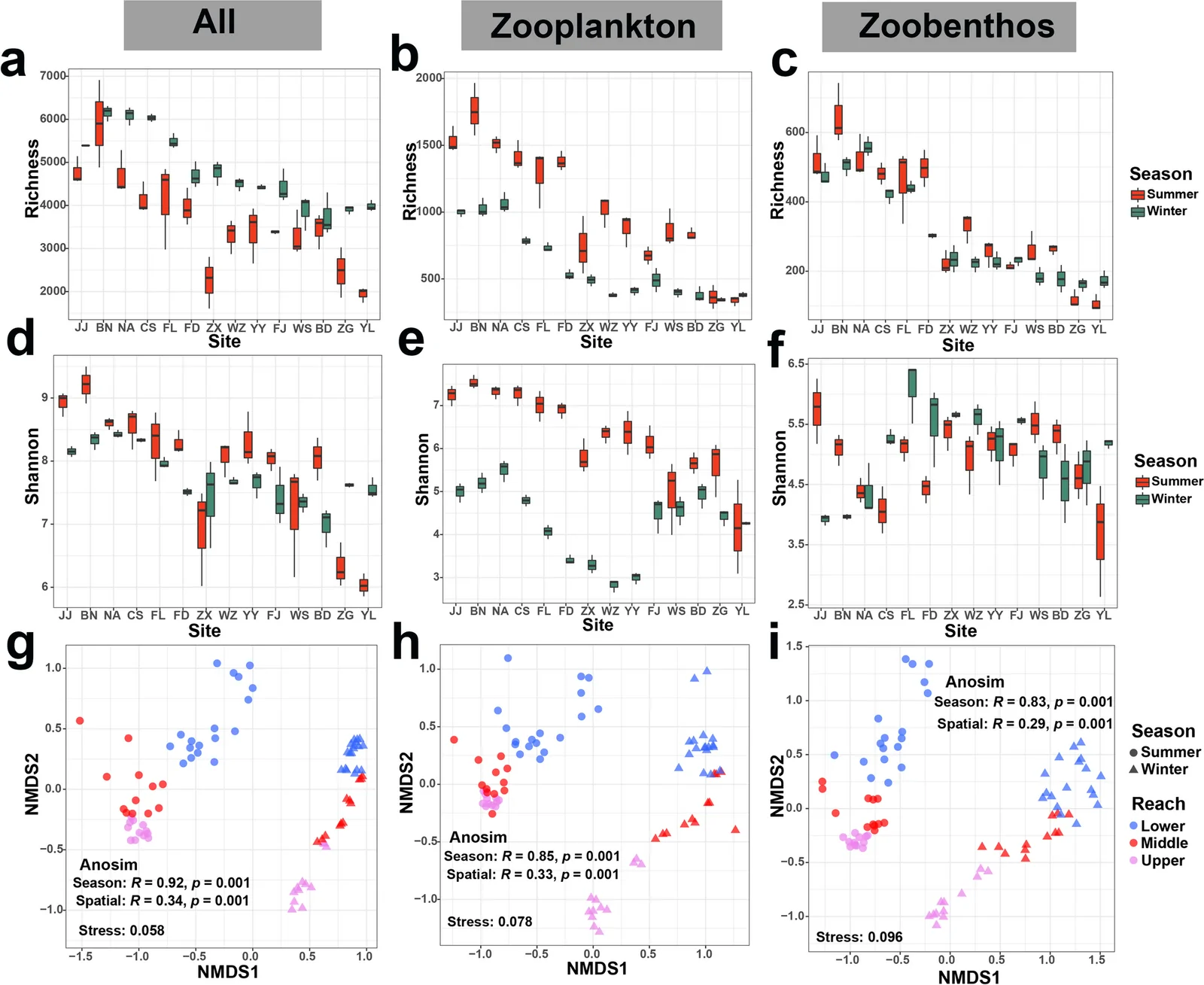
Diversity of meio- and micro-eukaryotes, zooplankton, and zoobenthos communities in the TGR. The ASV richness (**a–c**) and Shannon index (**d–f**) dynamics for these three communities at each site along the TGR in summer and winter. **g–i** Visualize the variation in the composition of these three communities across three reaches and two seasons through nonmetric multidimensional scaling (NMDS) ordination based on Bray-Curtis dissimilarity. The global *R* statistic quantifies the extent to which the variation in the compositions of these three communities can be attributed to either seasonal or spatial (reaches) factors, as determined by ANOSIM analysis

Overall, the richness and Shannon index of the meio- and micro-eukaryotes, along with its subtaxa (zooplankton and zoobenthos), displayed distinct temporal-spatial dynamics within the TGR (Figs. 3a–f, S3, and Table S4). Across both seasons, ASV richness declined for all three taxa along the river. The Shannon index showed a consistent pattern for meio- and micro-eukaryotes in both seasons and for zooplankton during summer. Interestingly, the Shannon index for winter zooplankton initially decreased and then increased, while the zoobenthos displayed the opposite trend. Additionally, the richness of zooplankton and the Shannon index of meio- and micro-eukaryotes and zooplankton during summer were significantly higher (*p* < 0.05) than in winter (Fig. S4). Notably, there was no significant difference (*p* > 0.05) in the richness and Shannon index of zoobenthos between summer and winter (Fig. S4).

Using Bray-Curtis-based NMDS analysis on 84 water samples, we identified remarkable seasonal and spatial variations in the composition of the three communities. Samples collected within the same season or from spatially close locations exhibited clustering (Fig. 3g–i). ANOSIM results confirmed notable compositional differences between the two seasons (*R* = 0.92 for meio- and micro-eukaryotes, *R* = 0.85 for zooplankton, and *R* = 0.83 for zoobenthos; *p* = 0.001). Additionally, these three taxa displayed a distinct spatial gradient distribution (*R* range 0.29–0.34; *p* = 0.001). This pattern was also reflected in the phylogenetic lineage tree for all samples across both seasons (Fig. S5).

**Fig. 4:**
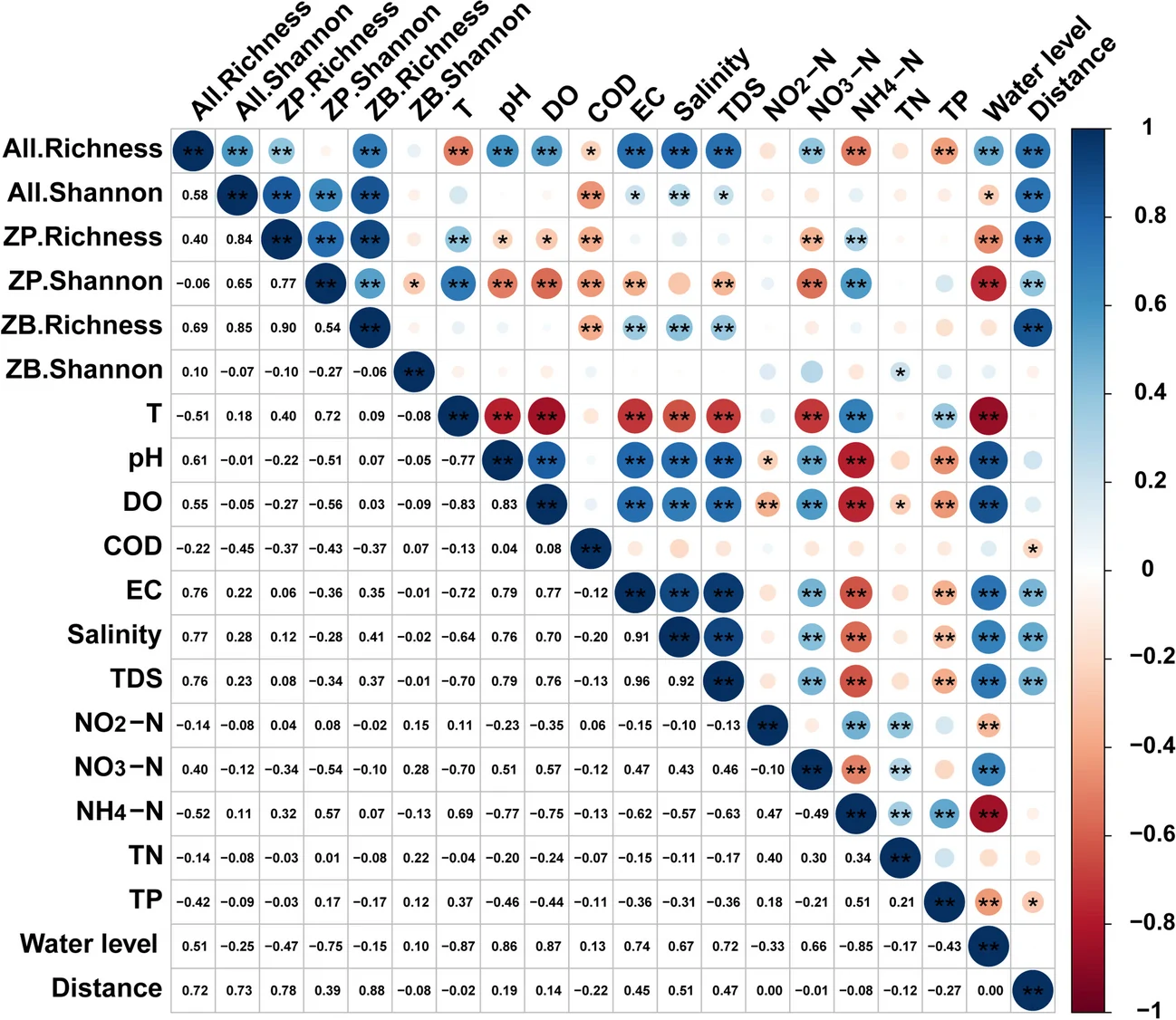
The correlation analysis heatmap illustrates the relationships between the environmental variables and the diversities of meio- and micro-eukaryotes, zooplankton, and zoobenthos. Heatmaps were created using Spearman’s correlation as a basis. Significance levels: *p < 0.05, **p < 0.01. All, all ASVs (meio- and micro-eukaryotic communities); ZP, zooplankton; ZB, zoobenthos; T, water temperature; DO, dissolved oxygen; COD, chemical oxygen demand; EC, electrical conductivity; TDS, total dissolved solids; NO_2_-N, nitrite nitrogen; NO_3_-N, nitrate nitrogen; NH_4_-N, ammonium nitrogen; TN, total nitrogen; TP, total phosphorus; Distance, distance from the dam

The correlation between the α-diversity indices of the three taxa and environmental variables in two seasons was assessed using Spearman’s correlation coefficient (Fig. 4). There was a noteworthy positive correlation among the different α-diversity indices, suggesting a robust interaction among meio- and micro-eukaryotic species. Most environmental variables exhibited a significant correlation with the diversity index, except for NO_2_-N. Interestingly, the distance from the dam and water level showed a strong positive and negative correlation with all α-diversity indices, respectively, implying substantial seasonal and spatial variations.

In particular, the α-diversity index (comprising All.richness, All.shannon, ZP.richness, ZP.shannon, and ZB.richness) and environmental variables (COD, EC, Salinity, TDS, and TP) showed significant correlations with the distance from the dam. This implies that the decline in diversity of meio- and micro-eukaryotes, zooplankton, and zoobenthos along the TGR might be linked to these environmental conditions.

### Fit to the neutral model of community assembly

**Fig. 5:**
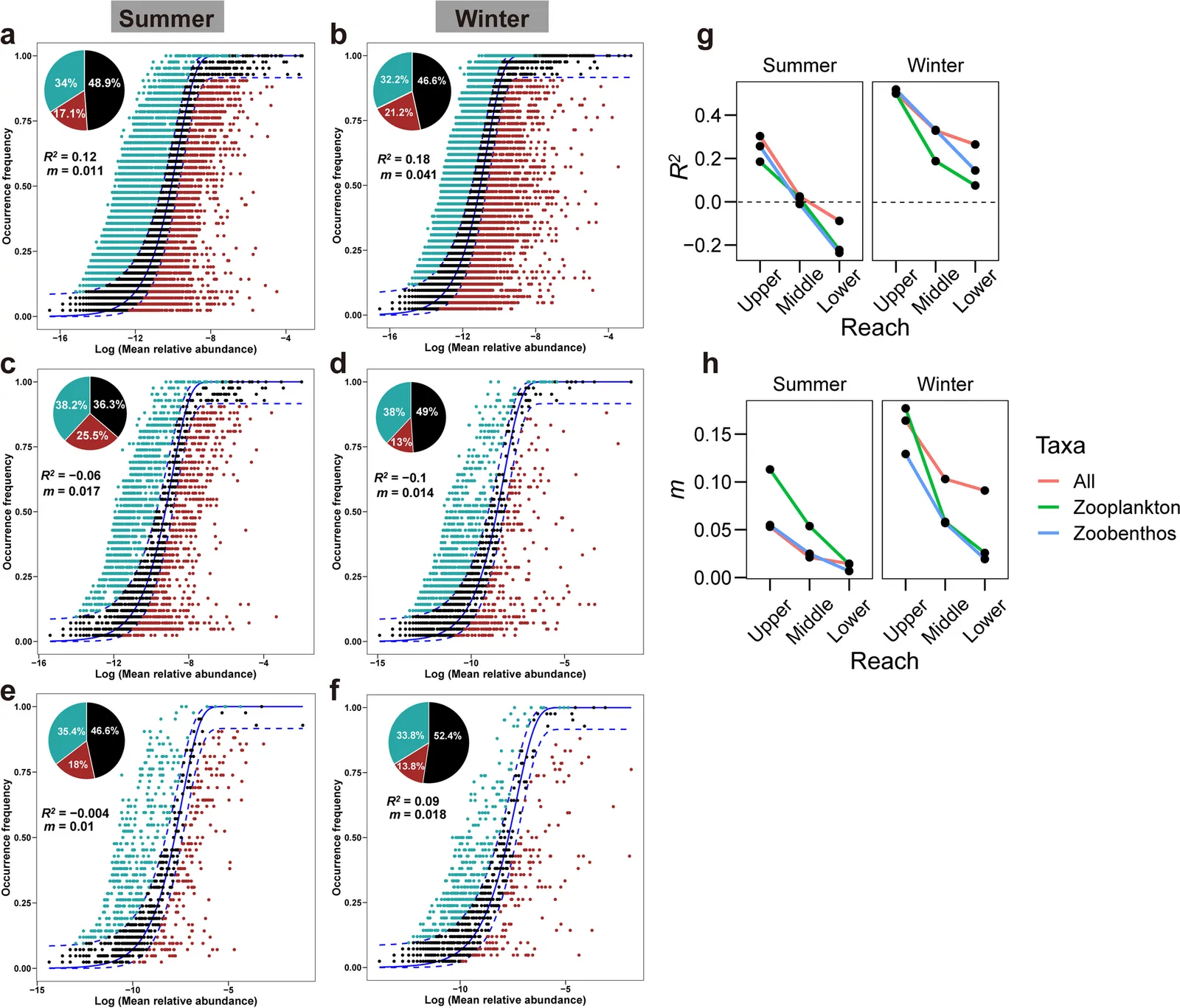
Fit of the neutral community model (NCM) for meio- and micro-eukaryotes, zooplankton, and zoobenthos community assembly. **a**–**f** The fit of the neutral model in summer (**a**, **c**, **e**) and winter (**b**, **d**, **f**) samples across the river for these three communities. The solid blue lines represent the best fit to the NCM, while the dashed blue lines indicate 95% confidence intervals around the model prediction. The occurrences of ASVs above, below, and within the prediction are shown as turquoise, red, and dark dots, respectively. The proportion of ASVs in different groups is also represented in the pie chart. **g**–**h** *R*^*2*^ and *m* values for fitting the neutral model to different reaches in summer and winter. Here, *m* represents the estimated immigration rate. A positive *R*^2^ value indicates a fit to the neutral model, while a negative *R*^2^ signifies a lack of fit to the neutral model

NCM analysis assessed the contribution of stochastic processes on meio- and micro-eukaryotic, zooplankton, and zoobenthos communities (Fig. 5a–f). In both summer and winter, the NCM gauged a weak or negative relationship (as indicated by *R*^2^ values) between ASV occurrence frequency and their relative abundance fluctuations. During both seasons, more than half of the ASVs deviated from the model’s predictions, with their occurrences being either higher or lower than predicted across all three communities. These results suggest a minor role of stochastic processes in determining community assembly in both summer and winter.

At a smaller scale (Fig. 5g), the *R*^2^ values for the NCM of the three communities decreased as we moved from the upper to lower reaches of the reservoir. This points to a growing significance of deterministic processes in community assembly. Likewise, the *m* values followed a decreasing pattern from the upper to lower reaches (Fig. 5h), suggesting that species dispersal for these three taxa was more prominent in the upper reaches (with high flow velocity) compared to the lower reaches (with low flow velocity) of the TGR.

### Seasonal, spatial, and environmental factors for community assembly

**Fig. 6:**
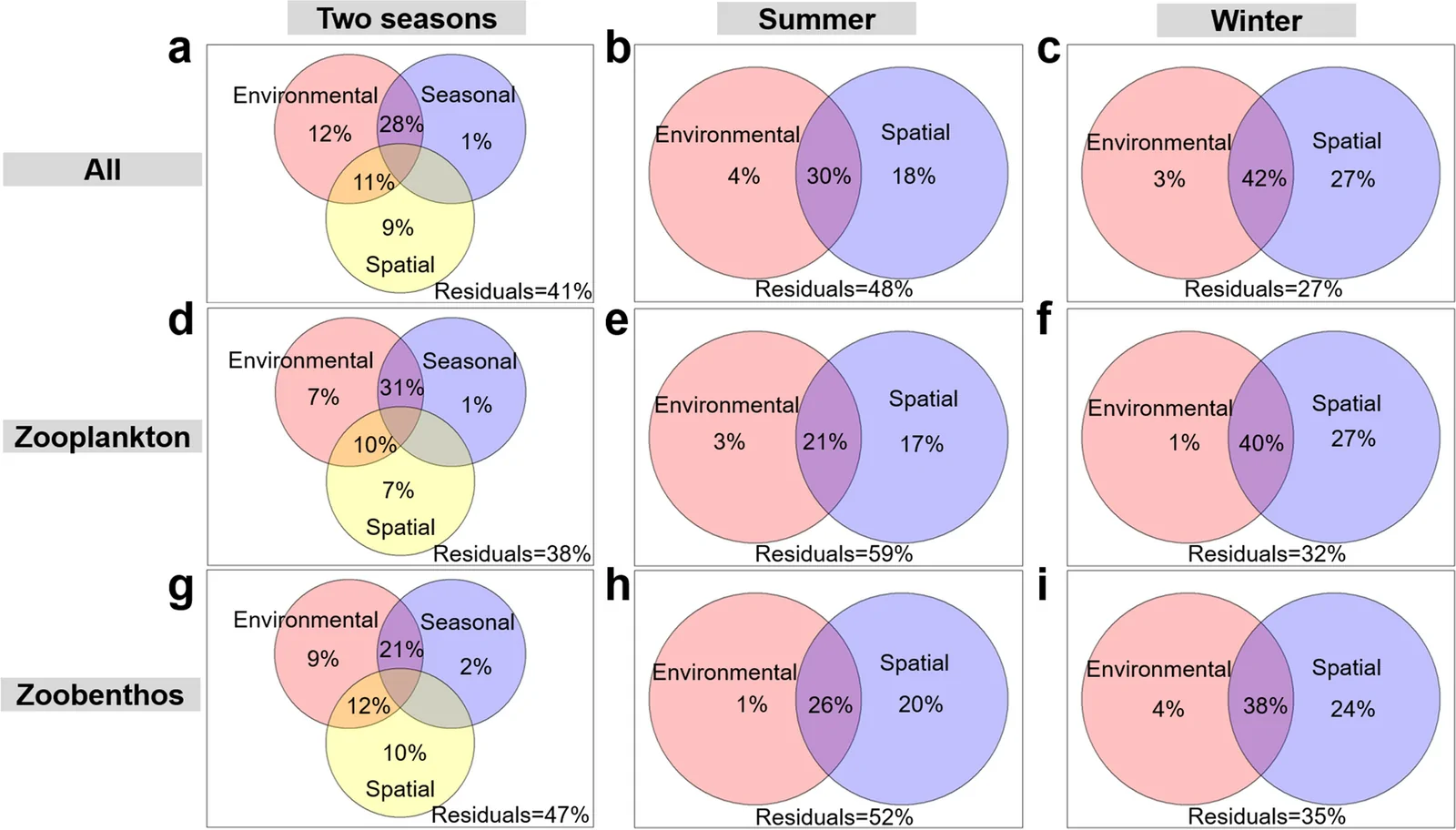
The variation partitioning analysis (VPA) demonstrates the relative contribution of seasonal, spatial, and environmental factors to variations in meio- and micro-eukaryotic, zooplankton, and zoobenthos communities. **a**–**i** The VPA diagram illustrates the proportional impact of seasonal, spatial, and environmental factors on the variability of these three communities in both dual seasons (**a**, **d**, **g**) and a single season (**b**, **c**, **e**, **f**, **h**, **i**). Only variables significantly affecting the communities, as determined by forward selection, are incorporated in the VPA analyses. All indicates all ASVs (meio- and micro-eukaryotic communities)

The results of the variance partitioning analysis (VPA) indicate how the variation within the three communities is influenced by seasonal, environmental, and spatial factors. Specifically, in two seasons, the impact of individual factors on community variation is as follows: Environmental and spatial variables alone exhibit a more substantial influence (ranging from 7 to 12% for environmental and 7 to 10% for spatial) compared to seasonal factors alone (which contribute only 1% for meio- and micro-eukaryotes, and zooplankton; and 2% for zoobenthos); Notably, the largest proportions of variation in the three communities can be attributed to the combined effects of both seasonal and environmental factors (ranging from 21 to 31%), highlighting their significant interplay in shaping the community (Fig. 6a, d, and g).

Irrespective of seasonality, the spatial factor alone exerts a stronger influence on these three communities within a single season compared to the environmental factor alone (Fig. 6b–c, e–f, and h–i). Moreover, as the water level rises (from summer to winter), the proportion of variance explained by spatial factors alone and the combined influence of spatial and environmental factors on these communities increases notably. This is accompanied by a noticeable decrease in the unexplained variance of these communities, underscoring the growing significance of deterministic processes in the community assembly of these three taxa from summer to winter.

**Fig. 7:**
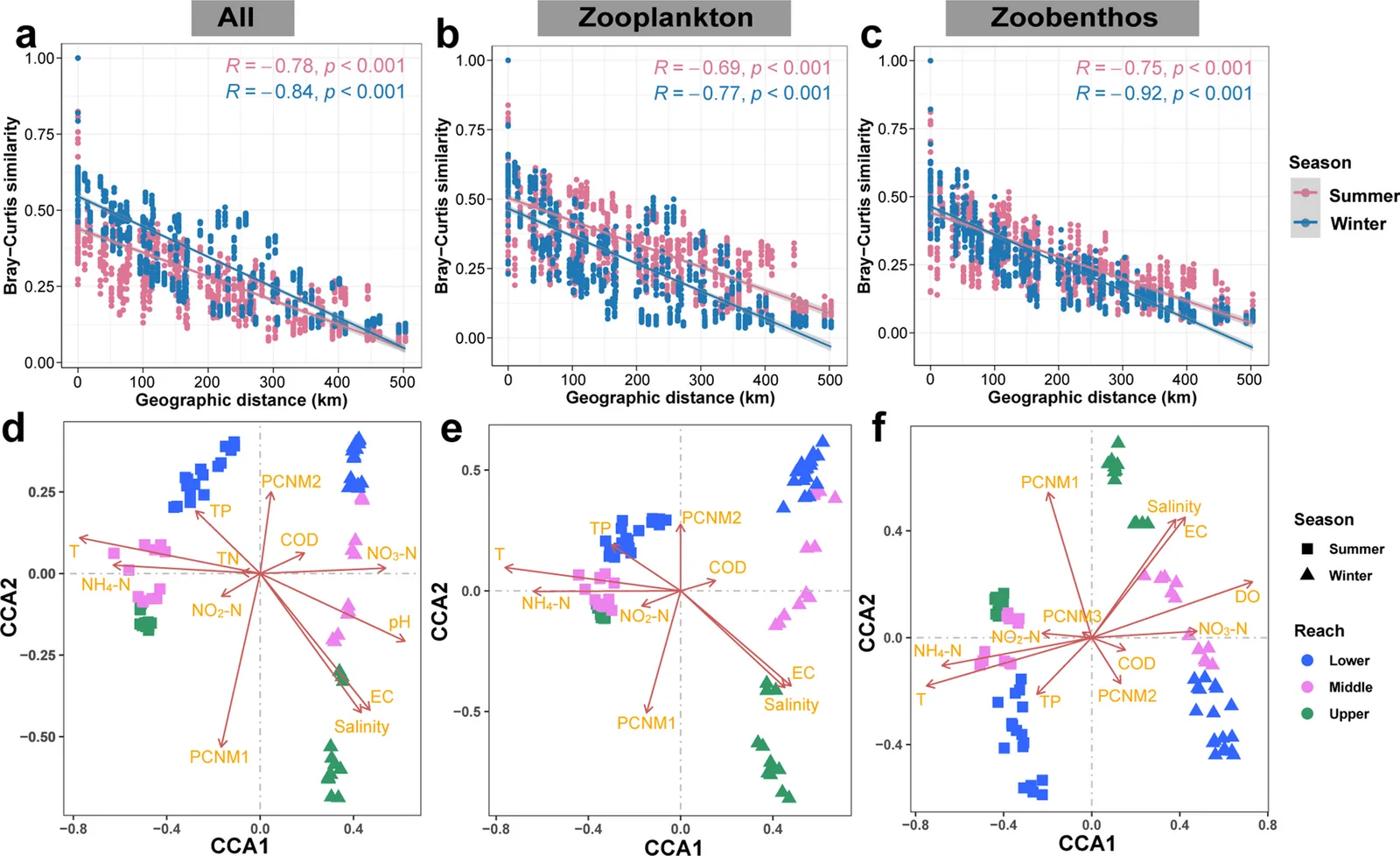
Driving factors of the compositions of meio- and micro-eukaryotic, zooplankton, and zoobenthos communities. **a**–**c** Distance-decay patterns based on the Bray-Curtis’ similarity of these three taxa compositions and cumulative geographic distance in summer and winter. **d–f** Canonical correspondence analysis (CCA) is applied to explain the compositions of these three communities about environmental and spatial variables. The CCA plots only display variables that were significantly identified to influence the communities through forward selection. Arrow lengths indicate the explanatory power for the respective variables. The term “all” refers to all ASVs comprising micro- and meio-eukaryotic communities. PCNMs stand for geographic factors derived from principal coordinate analysis of neighborhood matrices. Specific variables shown in the CCA plots include T (water temperature), DO (dissolved oxygen), COD (chemical oxygen demand), EC (electrical conductivity), NO_2_-N (nitrite nitrogen), NO_3_-N (nitrate nitrogen), NH_4_-N (ammonium nitrogen), TN (total nitrogen), and TP (total phosphorus)

To further explore the influence of spatial factors on community variation, we conducted a distance-decay analysis. The results revealed significant negative correlations between geographical distance and Bray-Curti’s similarity for these three communities during both seasons. Additionally, all three communities exhibited higher turnover rates in winter compared to summer (Fig. 7a–c).

However, the geographical distance alone was insufficient to illustrate the relevant spatial dispersal structures. To assess this, we conducted a CCA analysis to investigate the influence of 12 environmental variables and spatial factors (represented by 10 PCNM vectors indicating quantitative spatial patterns; Table S5, S6) on the three communities. The CCA biplot also revealed the spatiotemporal distribution pattern of these communities. Specifically, the communities sampled in summer and winter were positioned on opposite sides of the second axis (CCA2), while the communities from the three reaches of TGR exhibited repulsion along the first axis (CCA1) (Fig. 7d–f). Through forward selection (Table S7) and CCA analysis, we identified several significant environmental variables (e.g., T, Salinity, NO_2_-N, NH_4_-N, TP, EC, and COD), along with two spatial variables (PCNM1 and PCNM2), that notably influenced the composition of these three taxa.

Furthermore, during the summer seasons, TP was always related to the community at the lower reaches of these three taxa. Notably, the primary environmental variables affecting these three taxa were T, NH_4_-N, salinity, and EC. Meio- and micro-eukaryotes were additionally influenced by nitrate-nitrogen (NO_3_-N) and pH, while zoobenthos were additionally influenced by DO and NO_3_-N. These vital variables had the most pronounced influence on the reservoir’s middle reaches, indicating heightened sensitivity of the transition zone to changes in environmental conditions. Consistently, the communities of three reaches in the TGR all demonstrated significantly narrower mean habitat niche breadths (*Bcom*) in the middle reaches compared to the upper and lower reaches, in both summer and winter (Fig. S6).

### Co-occurrence network analysis of meio- and micro-eukaryotic communities

**Fig. 8:**
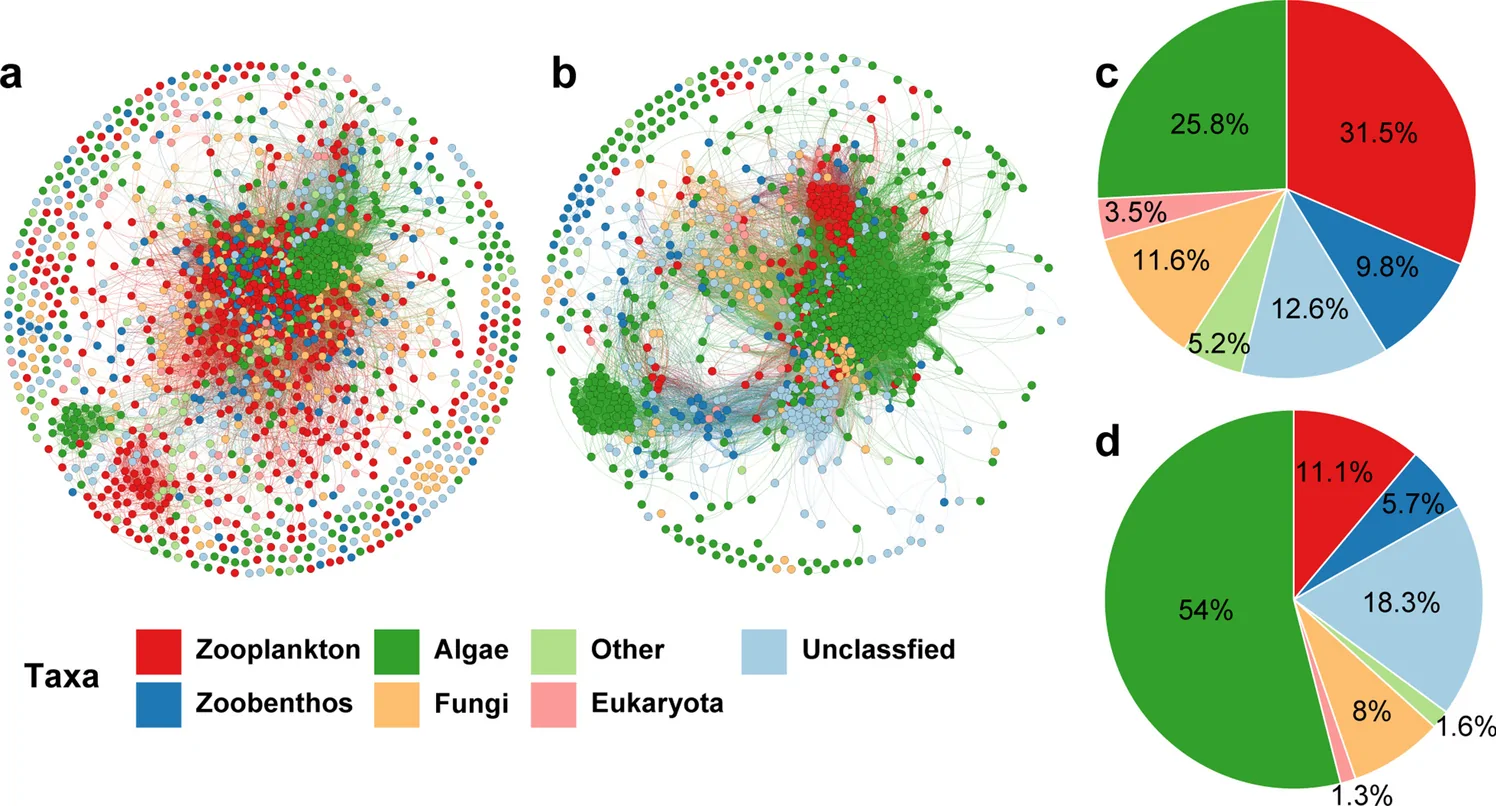
Network analysis of co-occurrence relations in the meio- and micro-eukaryotic communities. Co-occurrence networks were established for meio- and micro-eukaryotic communities during summer (**a**) and winter (**b**), operating at the ASV level. Each dot on the network corresponds to distinct ASVs, with connections indicating robust correlations (Spearman’s |*r*|> 0.7) that are statistically significant (BH-adjusted *p* < 0.01). Additionally, the proportions of taxa within the co-occurrence network were assessed for both summer (**c**) and winter (**d**). Eukaryota, unclassified eukaryotes; Other, other identified species not classified as algae, zooplankton, zoobenthos, and fungi; Unclassified, unidentified ASVs

Following an analysis of driving factors across three communities, over 27% of variations remained unexplained in the VPA (Fig. 6). Consequently, we explored biotic inter-domain interactions at the ASV levels between meio- and micro-eukaryotic communities (Fig. 8a, b, and Table 1). Using the same filtering parameters for ASV abundance tables, we retained 1608 ASVs present in both seasons. Notably, within the meio- and micro-eukaryotes, there were 1403 valid nodes (87.3%) in summer and 1449 (90.1%) in winter, indicating a strong relationship between meio- and micro-eukaryotes. In terms of network metrics, the winter network graph displayed higher edge numbers (200,889 vs. 71,303), average degree (277.27 vs. 101.64), and graph density (0.19 vs. 0.07) compared to summer, suggesting stronger interactions during winter. Notable trends include the dominance of algae (31.47%) and zooplankton (25.81%) in the network graph during summer, whereas only algae (53.97%) held dominance in winter (Fig. 8c, d).

**Table 1:** Topological parameters of networks for meio- and micro-eukaryotic communities in two seasons

Parameters	Summer (*n* = 42)		Winter (*n* = 42)
ASVs	1608		1608
Valid nodes	1403		1449
Proportion (%)	87.3		90.1
Edges	71303		200889
Average degree	101.6		277.3
Average path length	2.9		2.4
Graph diameter	10		9
Graph density	0.072		0.191
Average clustering coefficient	0.54		0.72
Betweenness centralization	0.030		0.014
Degree centralization	0.28		0.33
Modularity	0.27		0.18
Modules	64		36

## Discussion

The TGR plays a crucial role as a typical canal-type reservoir ecosystem for studying community ecology, due to its substantial size and the regular adjustment in water levels. Understanding the ecological processes that shape meio- and micro-eukaryotic communities in various habitats is vital within aquatic ecology. However, there is a lack of comprehension regarding the spatial-temporal patterns and assembly mechanisms of these communities, especially zooplankton and zoobenthos, in different habitats of vulnerable reservoir ecosystems affected by water level changes. In this study, utilizing eDNA-metabarcoding technology based on the COI gene, we systematically explored the biogeographic patterns of the three taxa at the community level to decipher their taxonomic diversity and community assembly mechanisms.

### eDNA sequencing based on COI effectively shows the spatiotemporal distribution of meio- and micro-eukaryotes in the TGR

Within aquatic habitats, a diverse range of eukaryotic organisms thrive, including zooplankton, zoobenthos, algae, fungi, fish, and insects (Adl et al. 2019). The application of eDNA-metabarcoding-based HTS has notably expedited the elucidation of eukaryotic communities (Zou et al. 2021). Previous research predominantly employed the COI gene as a metazoan barcode (Leray et al. 2013). In our study, we successfully employed universal COI gene primers to identify numerous meio- and micro-eukaryotes, such as zooplankton, zoobenthos, algae, and fungi (Table S8). Examination of ASVs in this study showed a substantial presence of algae (31.8%) and fungi (12.7%) (Fig. S1), underscoring the COI gene’s viability as a barcode for comprehensively studying the meio- and micro-eukaryotic community at eDNA level. This proposition gains further support from additional evidence. Firstly, the sequencing depth of ASVs in our study reached 380,000 (Table S1), a significant enhancement compared to microbial studies (Zhou and Ning 2017). Secondly, the reference database we built itself contained part COI gene sequence of algae and fungi, and the primers we use are degenerate. Thirdly, the clustering of three sampling replicates taken at distances of 50–100 m consistently occurred, with similar species composition observed between closely situated sites (Figs. 2, 3, and 7, and S5). For instance, the sampling sites in Zigui (pre-dam) and Yiling (post-dam) are only 13 km apart and exhibited similar species composition. This congruence underscores the reliability of our sequencing outcomes. Fourthly, the COI genes exist in the mitochondria of both fungi and algae, and previous research has also explored COI genes as their barcodes (Gong et al. 2018; Guo et al. 2015; Min and Hickey 2007). Several previous studies (Sawaya et al. 2019; Suter et al. 2021; Wangensteen et al. 2018; Yan et al. 2010) have also utilized HTS to identify numerous fungi and algae. Finally, this study identified zoobenthos (1501 ASVs) inhabiting the waterbed through surface water samples collection (Figs. 2 and S1). This implies that eDNA sequencing of surface water is a feasible method for assessing the communities of the whole water. As a result, COI barcode-based HTS introduces a novel approach to studying meio- and micro-eukaryotic compositions within aquatic environments.

Species composition and distribution change gradually along a gradient from lotic to lentic conditions within the river-reservoir system. Downstream lacustrine environments tend to host lentic-adapted species, whereas upstream riverine and transitional habitats are favored by lotic-adapted species (Yang et al. 2021). This study also reveals distinctive spatiotemporal dynamics in the relative abundance of meio- and micro-eukaryotes within TGR. These dynamics exhibit a complementary pattern, facilitating adaptation to the transitions between lotic and lentic conditions resulting from dam regulation (Fig. 2). In general, there is a gradual increase in algae from upstream to downstream, in contrast to the gradual decrease observed in zooplankton and zoobenthos populations. Additionally, from summer to winter, the fungi and algae increase significantly, while zooplankton and zoobenthos decrease.

### The TGD may affect the meio- and micro-eukaryotic biodiversity in the reservoir

Zooplankton and zoobenthos are large and highly diverse groups within meio- and micro-eukaryotic communities in aquatic ecosystems. The microorganisms, particularly zooplankton and zoobenthos, are highly sensitive to specific local environmental conditions, often serving as environmental indicator species for assessing ecosystem health. Along the TGR, the diversity of these three taxa exhibits a declining trend (Fig. 3), which might be attributed to the environmental impact of TGD impoundment. Reservoirs and dams are thought to have a significant impact on the biogeochemical cycles, hydro-geomorphology, and overall ecological conditions of river ecosystems (Nilsson et al. 2005; Zhang et al. 2020). A study conducted in Xiangxi Bay, a TGR tributary, discovered that the upper reaches displayed the highest diversity of picoeukaryotes, while the middle reaches exhibited the lowest species diversity (Li et al. 2018). The TGD impoundment is likely to alter water chemistry and physical attributes, reducing self-purification capacity, and increasing pollutant retention, ultimately leading to an impact on biodiversity (Tan et al. 2022; Xiang et al. 2021).

We conducted a correlation analysis to explore potential reasons contributing to the decline in biodiversity (Fig. 4). The results indicated the diversity index and several environmental variables (including COD, TP, EC, salinity, and TDS) had a significant correlation with the distance from the dam. This implies that these environmental variables related to the distance from the dam may be contributing to the reduction in diversity (McLusky and Elliott 2007; Zhou et al. 2021). Previous research has demonstrated that salinity gradients can directly impact local communities, leading to a decrease in the variety of benthic macrofaunal species (Grattepanche et al. 2016). Additionally, both COD and TP serve as vital indicators of pollution in aquatic environments, exerting substantial influence on the diversity of aquatic organisms (Tee et al. 2021). Therefore, the TGD is likely to influence the diversity and composition of meio- and micro-eukaryotes. Recognizing this connection between dam-induced environmental changes and biodiversity is crucial when establishing a baseline for long-term biological protection in the TGR.

### The contribution of stochastic processes to community assembly decreases along the reservoir

Examining the variations and assembly processes of the meio- and micro-eukaryotic community along the TGR is vital for preserving biodiversity and stability in this river-reservoir ecosystem. Our study reveals a minor influence of stochastic processes on shaping the three communities (Fig. 5a–f), aligning with previous research (Liu et al. 2022b; Wang et al. 2022). This implies that alternative mechanisms, such as deterministic factors and species interactions, may dominate community assembly.

Notably, when the TGR is segmented into upper, middle, and lower reaches, we observe a gradual decrease in the relative importance (*R*^*2*^) of stochastic processes in these three communities along the river, regardless of the season (Fig. 5g). This can be attributed to several factors: (1) inherent differences such as a gradual reduction in flow velocity exist within the three reaches of the TGR. Additionally, meio- and micro-eukaryotes also exhibit distinct variations in dispersal potential, metabolic activity, and body size (Keeling and Del Campo 2017; Wu et al. 2018). These differences could potentially influence the relative importance of stochastic processes. (2) As the distance span increases from the upper to lower reaches (Fig. 1), the dispersal ability of meio- and micro-eukaryotic communities across the TGR may differ between river sections (Fig. 5h), and the estimated immigration rate (*m*) tends to decrease with greater distance gradients (Chase and Myers 2011). (3) In proximity to the dam, a more pronounced impact of environmental selection on the three taxa may exist (Gao et al. 2021b; Li et al. 2022).

### Deterministic factors and species interactions shape the meio- and micro-eukaryotic communities

Based on the VPA results, we observed that deterministic factors, such as seasonal, environmental, and spatial variables, played a significant role in shaping the meio- and micro-eukaryotic community, along with its two subcommunities (Fig. 6). These organisms, including zooplankton, zoobenthos, and algae, often serve as indicator species, demonstrating sensitivity to environmental shifts. Notably, dam construction triggered alterations in hydrological conditions within the reservoir area, mainly regulated by dam-controlled water storage and discharge. Consequently, the hydrological state becomes closely associated with spatial distance during specific time frames. Through CCA analysis, we determined that environmental variables predominantly drive seasonal community fluctuations, whereas hydrological conditions (represented as spatial distance) primarily influence spatial variances (Fig. 7).

Previous studies have demonstrated that abiotic environmental conditions can directly or indirectly influence meio- and micro-eukaryotic communities in aquatic ecosystems (Gad et al. 2020; Mo et al. 2021; Xue et al. 2018). Within the environmental parameters examined in this study, salinity, T, NH_4_-N, and EC contributed the most to driving the seasonal variations of these three communities in the TGR (Fig. 7d–f). Our findings reinforce that the transition zone is more vulnerable compared to the riverine and lacustrine zones in the river-reservoir system at community levels (Qin et al. 2021). This is due to the pronounced influence of these key environmental parameters on the community structure in the middle reaches of the TGR (Fig. 7d–f). Intriguingly, the calculation of habitat niche breadth for the three reaches in both seasons indicates that the transition zone exhibits the smallest *Bcom*, making it highly susceptible to environmental influences (Fig. S6).

We observed significant and strong negative correlations between geographical distance and Bray-Curtis similarity in these three communities (Fig. 7a–c), which align with findings from numerous previous studies (Chen et al. 2019; Isabwe et al. 2019; Wu and Liu 2018; Zou et al. 2021). This significant and strong distance-decay relationship may arise from species sorting and dispersal capabilities (Hanson et al. 2012). Species sorting, as a mechanism for adapting to local environments, contributes to the formation of distance-decay relationships. Our VPA analysis indicates that the majority of community variation in both summer and winter can be attributed to the combined influence of spatial and environmental factors (Fig. 6). This underscores the contribution of the spatially structured environment to the compositional variability within the three communities. Additionally, given the scale-dependent nature of distance-decay patterns in microorganisms, it becomes essential to consider both historical influences (such as spatial distance and past environmental conditions) and current environmental conditions (Astorga et al. 2012). Historical influences (resulting from past environmental conditions) can lead to spatial variability, and their legacies are retained during the dispersal process (Ge et al. 2008). On a larger scale covering the entire TGR (over 600 km), spatial variables (17–27%) exerted a more substantial influence on the variation in these three communities during single seasons than environmental variables (1–4%). Notably, a robust distance-decay pattern was observed in all three communities across both seasons, implying that distinct meio- and micro-eukaryotic taxa under similar hydrologic conditions (at high and low water levels) exhibited a consistent spatial pattern.

During the impoundment of the TGD from summer to winter, this study found that spatial factors played a significant role in influencing the composition of these three communities. VPA analysis confirmed a significant increase in their explanatory proportion (Fig. 6). Additionally, the distance-decay patterns (Fig. 7a–c) indicated that community dissimilarity increased in winter, underscoring the stronger influence of spatial factors on communities during this season. This effect can be attributed to the uneven habitat of the TGR, characterized by its wide expanse and reduced river connectivity due to dams. In winter, the decreased flow velocity leads to increasing environmental heterogeneity, promoting increased dissimilarity among communities (Isabwe et al. 2018).

The unexplained portion, as determined by the VPA analysis of measured environmental and spatial variables in this study, exceeded 27% (Fig. 6). This phenomenon implies the existence of unmeasured environmental variables and biological factors (like species interactions). These factors likely played a significant role in explaining the variation within the three communities (Lima-Mendez et al. 2015).

Previous studies have indicated that interactions within species and across different domains play a crucial role in shaping meio- and micro-eukaryotic communities in aquatic ecosystems (Berdjeb et al. 2018; Sommer et al. 2012). In this study, we conducted a co-occurrence network analysis at the ASV level among these communities, revealing a strong interconnection between ASVs (Fig. 8). Our species classification identified algae and zooplankton as the primary components in the summer network graph, highlighting a substantial predator-prey interaction between them. Comparable interaction patterns have been observed in marine environments (Calbet and Landry 2004). Moreover, during winter, algae accounted for 54% of the network diagram, underscoring their pivotal role as suppliers of nutrients and energy for other organisms in the aquatic food web. This transformation is crucial for maintaining taxa coexistence during the winter season. Consequently, the robust species-species connection (Fig. 8 and Table 1) and the complementary distribution pattern (Fig. 2) within the TGR underscore the coevolution between algae and other meio- and micro-eukaryotes. This coevolution suggests that algae and other organisms subject each other to selective pressures, fostering rapid swift adaptive responses (Liu et al. 2022b). Historically, antagonistic interactions have been pivotal drivers of evolutionary processes (Mougi and Iwasa 2011).

## Supplementary Information

Below is the link to the electronic supplementary material.

Supplementary file1 (PDF 1035 KB)

Supplementary file2 (XLSX 861 KB)

## Data Availability

The sequencing data can be found on GenBank under BioProject number PRJNA870158, accessible through this live link: https://www.ncbi.nlm.nih.gov/bioproject/PRJNA870158.
