# Supplementary material for: Biogeographic patterns of meio- and micro-eukaryotic communities in dam-induced river-reservoir systems
Source: Appl Microbiol Biotechnol. 2024 Jan 15;108:130. doi: 10.1007/s00253-023-12993-4 (PMC10789839; doi:10.1007/s00253-023-12993-4)
Supplement: Supplementary file 1 [file 253_2023_12993_MOESM1_ESM.pdf]

Supplementary Information

Biogeographic patterns of meio- and micro-eukaryotic communities in dam-induced river-reservoir systems

Huan Hu[1,2], Xing-Yi Wei[1,2], Li Liu[2], Yuan-Bo Wang[1,2], Ling-Kang Bu[2,3], Huang-Jie Jia[2], De-Sheng Pei[3*]

[1] Chongqing Jiaotong University, Chongqing 400074, China

[2] Chongqing Institute of Green and Intelligent Technology, Chongqing School of University of Chinese Academy of Sciences, Chinese Academy of Sciences, Chongqing 400714, China

[3] School of Public Health, Chongqing Medical University, Chongqing 400016, China

*Corresponding author

E-mail: peids@cqmu.edu.cn (D.S.P)

Table S1. Summary of sampling, environmental variables, and sequences in the Three Gorges Reservoir (TGR) in the summer and winter

		Sampling information									Environmental variables												Sequencing information		
SampleID	Site	Location	Longitude	Latitude	Distance from dam	Reach	Water body	Sampling date	Season	Water level	T	pH	DO	COD	EC	Salinity	TDS	NO2-N	NO3-N	NH4-N	TN	TP	Sequencing depth	Bases	Average length
			(E)	(N)	(m)					(m)	(℃)		(mg/L)	(mg/L)	µS/cm	(mg/L)	(mg/L)	(mg/L)	(mg/L)	(mg/L)	(mg/L)	(mg/L)		(bp)	(bp)
S1-1	JJ	Jiangjin	106.2207	29.21483	687946	Upper	Riverine zone	2021/8/20	Summer	146.5	29.3	7.96	7.21	12.86	384	199	268	0.009	1.321	0.164	1.877	0.071	679890	212485821	312.53
S1-2	JJ	Jiangjin	106.2207	29.21483	687946	Upper	Riverine zone	2021/8/20	Summer	146.5	28.4	7.95	7.21	11.43	383	183	268	0.015	1.572	0.191	1.929	0.129	560559	175228414	312.6
S1-3	JJ	Jiangjin	106.2207	29.21483	687946	Upper	Riverine zone	2021/8/20	Summer	146.5	28.3	7.76	7.20	10.00	384	180	268	0.008	1.605	0.138	2.054	0.105	693233	216597918	312.45
S2-1	BN	Banan	106.5426	29.473633	620510	Upper	Riverine zone	2021/8/20	Summer	146.5	28.1	7.99	7.26	10.00	385	187	277	0.024	1.216	0.112	1.617	0.047	674780	210906077	312.56
S2-2	BN	Banan	106.5426	29.473633	620510	Upper	Riverine zone	2021/8/20	Summer	146.5	29.3	7.94	7.37	11.43	382	185	261	0.021	1.321	0.112	1.575	0.028	666077	208188857	312.56
S2-3	BN	Banan	106.5426	29.473633	620510	Upper	Riverine zone	2021/8/20	Summer	146.5	27.6	8.00	7.61	8.57	396	180	272	0.014	1.362	0.112	1.711	0.035	657373	205471637	312.56
S3-1	NA	Nanan	106.5841	29.602583	600876	Upper	Riverine zone	2021/8/20	Summer	146.5	27.1	7.95	7.76	14.29	417	190	287	0.015	1.216	0.224	1.586	0.148	656492	205219110	312.6
S3-2	NA	Nanan	106.5841	29.602583	600876	Upper	Riverine zone	2021/8/20	Summer	146.5	30.2	7.85	7.61	12.86	405	187	282	0.009	1.410	0.191	1.617	0.050	571381	178542439	312.48
S3-3	NA	Nanan	106.5841	29.602583	600876	Upper	Riverine zone	2021/8/20	Summer	146.5	28.9	7.95	7.41	12.86	398	189	277	0.013	1.402	0.257	1.669	0.074	748688	234013993	312.57
S4-1	CS	Changshou	107.0894	29.802133	526643	Upper	Riverine zone	2021/8/20	Summer	146.5	29.1	8.02	7.41	10.00	392	187	277	0.017	1.176	0.270	1.565	0.099	686666	214637353	312.58
S4-2	CS	Changshou	107.0894	29.802133	526643	Upper	Riverine zone	2021/8/20	Summer	146.5	29.7	7.98	7.31	8.57	391	187	287	0.015	1.233	0.283	1.544	0.102	552314	172693570	312.67
S4-3	CS	Changshou	107.0894	29.802133	526643	Upper	Riverine zone	2021/8/20	Summer	146.5	28.1	7.99	7.70	9.29	383	182	262	0.015	1.257	0.336	1.575	0.074	735163	229769110	312.54
S5-1	FL	Fuling	107.5274	29.858788	450995	Middle	Tranzition zone	2021/8/21	Summer	146.5	27.9	7.93	7.70	24.29	377	175	262	0.014	1.135	0.164	1.690	0.178	695580	217675490	312.94
S5-2	FL	Fuling	107.5274	29.858788	450995	Middle	Tranzition zone	2021/8/21	Summer	146.5	28.0	7.90	7.53	27.14	377	176	264	0.015	1.176	0.191	1.659	0.071	718257	224838714	313.03
S5-3	FL	Fuling	107.5274	29.858788	450995	Middle	Tranzition zone	2021/8/21	Summer	146.5	28.7	7.97	7.57	31.43	376	179	266	0.012	1.402	0.191	1.544	0.041	656526	205371330	312.82
S6-1	FD	Fengdu	107.7204	29.871405	431048	Middle	Tranzition zone	2021/8/21	Summer	146.5	29.9	7.91	7.28	18.57	375	182	257	0.012	1.087	0.434	1.617	0.059	548065	171425676	312.78
S6-2	FD	Fengdu	107.7204	29.871405	431048	Middle	Tranzition zone	2021/8/21	Summer	146.5	31.1	7.92	7.33	14.29	383	180	266	0.013	1.014	0.434	1.565	0.065	698655	218479610	312.71
S6-3	FD	Fengdu	107.7204	29.871405	431048	Middle	Tranzition zone	2021/8/21	Summer	146.5	29.6	7.85	7.35	10.00	388	183	272	0.014	1.051	0.638	1.617	0.059	678083	212082627	312.77
S7-1	ZX	Zhongxian	107.9977	30.26792	372779	Middle	Tranzition zone	2021/8/21	Summer	146.5	32.3	7.83	6.65	27.14	377	177	262	0.043	1.095	0.612	1.709	0.071	623787	194055851	311.09
S7-2	ZX	Zhongxian	107.9977	30.26792	372779	Middle	Tranzition zone	2021/8/21	Summer	146.5	32.8	7.86	6.98	22.86	370	168	257	0.025	0.966	0.401	1.709	0.053	703106	219931521	312.8
S7-3	ZX	Zhongxian	107.9977	30.26792	372779	Middle	Tranzition zone	2021/8/21	Summer	146.5	31.8	7.83	6.92	22.86	371	168	253	0.031	1.184	0.454	1.709	0.062	628040	196486793	312.86
S8-1	WZ	Wanzhou	108.3895	30.794857	283706	Middle	Tranzition zone	2021/8/21	Summer	146.5	27.4	7.74	7.74	14.29	380	179	266	0.009	1.475	0.217	1.804	0.068	748199	234165227	312.97
S8-2	WZ	Wanzhou	108.3895	30.794857	283706	Middle	Tranzition zone	2021/8/21	Summer	146.5	26.8	7.50	7.50	17.14	378	181	261	0.014	1.499	0.270	1.752	0.050	660284	206628762	312.94
S8-3	WZ	Wanzhou	108.3895	30.794857	283706	Middle	Tranzition zone	2021/8/21	Summer	146.5	27.2	7.53	7.53	12.86	379	178	264	0.018	1.499	0.362	1.898	0.062	651532	203961050	313.05
S9-1	YY	Yunyang	108.7199	30.920005	241557	Lower	Tranzition zone	2021/8/22	Summer	146.5	27.4	8.01	7.78	12.86	378	178	263	0.017	1.499	0.270	1.607	0.059	721466	226024786	313.29
S9-2	YY	Yunyang	108.7199	30.920005	241557	Lower	Tranzition zone	2021/8/22	Summer	146.5	27.7	7.95	7.63	12.86	370	177	263	0.016	1.491	0.257	1.721	0.059	521635	163432171	313.31
S9-3	YY	Yunyang	108.7199	30.920005	241557	Lower	Tranzition zone	2021/8/22	Summer	146.5	28.9	7.97	7.39	14.29	370	178	261	0.020	1.467	0.322	1.809	0.077	547937	171808256	313.55
S10-1	FJ	Fengjie	109.5051	31.03669	162083	Lower	Lacustrine zone	2021/8/22	Summer	146.5	29.0	7.93	7.01	17.14	370	178	255	0.028	1.435	0.336	1.742	0.105	693191	217367690	313.58
S10-2	FJ	Fengjie	109.5051	31.03669	162083	Lower	Lacustrine zone	2021/8/22	Summer	146.5	30.1	7.94	6.90	14.29	367	174	257	0.025	1.427	0.336	1.981	0.056	500048	156627808	313.23
S10-3	FJ	Fengjie	109.5051	31.03669	162083	Lower	Lacustrine zone	2021/8/22	Summer	146.5	30.1	7.72	6.80	15.71	367	171	256	0.022	1.427	0.336	1.867	0.059	695227	217674541	313.1
S11-1	WS	Wushan	109.9171	31.059443	119292	Lower	Lacustrine zone	2021/8/22	Summer	146.5	27.1	7.84	7.38	18.57	368	173	256	0.013	1.443	0.336	1.791	0.065	732172	228742044	312.42
S11-2	WS	Wushan	109.9171	31.059443	119292	Lower	Lacustrine zone	2021/8/22	Summer	146.5	27.7	7.97	7.23	20.00	365	174	258	0.014	1.459	0.296	1.769	0.068	578789	181033627	312.78
S11-3	WS	Wushan	109.9171	31.059443	119292	Lower	Lacustrine zone	2021/8/22	Summer	146.5	28.2	7.98	7.15	17.14	367	177	254	0.017	1.459	0.309	1.785	0.074	655481	204887835.5	312.6
S12-1	BD	Badong	110.3321	31.047456	75183	Lower	Lacustrine zone	2021/8/23	Summer	146.5	25.3	7.95	7.77	17.14	392	183	273	0.025	1.330	0.467	1.822	0.114	587158	183882277	313.17
S12-2	BD	Badong	110.3321	31.047456	75183	Lower	Lacustrine zone	2021/8/23	Summer	146.5	25.6	7.90	7.66	12.86	391	185	271	0.023	1.354	0.362	1.738	0.151	697390	218986010	314.01
S12-3	BD	Badong	110.3321	31.047456	75183	Lower	Lacustrine zone	2021/8/23	Summer	146.5	25.8	7.95	7.60	10.00	393	185	272	0.027	1.548	0.428	2.003	0.090	655254	205133282	313.06
S13-1	ZG	Zigui	110.9735	30.845309	3685	Lower	Lacustrine zone	2021/8/23	Summer	146.5	26.0	7.75	7.19	12.86	364	177	256	0.030	1.427	0.441	1.898	0.191	644245	201598441	312.92
S13-2	ZG	Zigui	110.9735	30.845309	3685	Lower	Lacustrine zone	2021/8/23	Summer	146.5	26.0	7.75	7.15	15.00	360	172	257	0.030	1.613	0.454	2.097	0.080	566510	177259055	312.9
S13-3	ZG	Zigui	110.9735	30.845309	3685	Lower	Lacustrine zone	2021/8/23	Summer	146.5	25.9	7.93	7.31	17.14	365	180	251	0.031	1.394	0.520	1.945	0.108	721980	225937827	312.94
S14-1	YL	Yiling	111.0823	30.856624	-9035	Lower	Lacustrine zone	2021/8/23	Summer	146.5	26.1	7.95	7.43	24.29	368	173	256	0.031	1.435	0.507	1.973	0.240	538717	168552786	312.88
S14-2	YL	Yiling	111.0823	30.856624	-9035	Lower	Lacustrine zone	2021/8/23	Summer	146.5	26.1	7.65	7.29	22.86	366	172	254	0.030	1.265	0.401	1.696	0.062	604933	189568013	313.37
S14-3	YL	Yiling	111.0823	30.856624	-9035	Lower	Lacustrine zone	2021/8/23	Summer	146.5	26.3	7.93	7.22	18.57	379	175	258	0.032	1.362	0.428	1.822	0.071	705806	221047507	313.18
W1-1	JJ	Jiangjin	106.2207	29.21483	687946	Upper	Riverine zone	2021/12/26	Winter	172	14.8	8.32	9.41	23.33	427	204	297	0.012	1.396	0.057	1.567	0.043	710287	222587945	313.38
W1-2	JJ	Jiangjin	106.2207	29.21483	687946	Upper	Riverine zone	2021/12/26	Winter	172	14.4	8.26	9.31	23.33	421	202	299	0.012	1.383	0.037	1.588	0.046	502047	157331506	313.38
W1-3	JJ	Jiangjin	106.2207	29.21483	687946	Upper	Riverine zone	2021/12/26	Winter	172	14.5	8.27	9.65	23.33	420	199	294	0.012	1.401	0.047	1.722	0.037	603030	189081032	313.55
W2-1	BN	Banan	106.5426	29.473633	620510	Upper	Riverine zone	2021/12/26	Winter	172	14.5	8.33	9.51	15.00	421	203	296	0.017	1.579	0.107	1.804	0.031	696372	218501560	313.77
W2-2	BN	Banan	106.5426	29.473633	620510	Upper	Riverine zone	2021/12/26	Winter	172	14.1	8.21	9.47	15.00	420	200	292	0.017	1.557	0.153	1.753	0.022	749246	234969386	313.61
W2-3	BN	Banan	106.5426	29.473633	620510	Upper	Riverine zone	2021/12/26	Winter	172	14.1	8.25	9.48	15.00	421	199	294	0.017	1.570	0.072	1.814	0.028	580427	181921064	313.43
W3-1	NA	Nanan	106.5841	29.602583	600876	Upper	Riverine zone	2021/12/26	Winter	172	13.8	8.29	9.49	11.67	417	201	291	0.016	1.610	0.098	1.845	0.034	694508	217455925	313.11
W3-2	NA	Nanan	106.5841	29.602583	600876	Upper	Riverine zone	2021/12/26	Winter	172	13.7	8.15	9.42	11.67	415	197	287	0.017	1.583	0.080	1.794	0.040	688670	216086395	313.77
W3-3	NA	Nanan	106.5841	29.602583	600876	Upper	Riverine zone	2021/12/26	Winter	172	14.0	8.23	9.25	11.67	417	195	293	0.016	1.561	0.083	1.742	0.022	569868	178561989	313.34
W4-1	CS	Changshou	107.0894	29.802133	526643	Upper	Riverine zone	2021/12/26	Winter	172	13.6	8.25	9.53	15.00	425	200	300	0.015	1.771	0.118	1.742	0.018	549197	172600493	314.28
W4-2	CS	Changshou	107.0894	29.802133	526643	Upper	Riverine zone	2021/12/26	Winter	172	13.8	8.25	9.58	15.00	422	204	297	0.015	1.539	0.108	1.742	0.020	514742	162013740	314.75
W4-3	CS	Changshou	107.0894	29.802133	526643	Upper	Riverine zone	2021/12/26	Winter	172	14.3	8.25	9.47	15.00	425	199	291	0.015	1.561	0.058	1.732	0.022	648804	204180178	314.7
W5-1	FL	Fuling	107.5274	29.858788	450995	Middle	Tranzition zone	2021/12/26	Winter	172	14.4	8.35	9.23	13.33	422	208	297	0.021	1.543	0.077	1.794	0.049	406088	127442806	313.83
W5-2	FL	Fuling	107.5274	29.858788	450995	Middle	Tranzition zone	2021/12/26	Winter	172	14.1	8.20	9.13	13.33	418	196	295	0.019	1.530	0.053	1.742	0.043	450752	141545393	314.02
W5-3	FL	Fuling	107.5274	29.858788	450995	Middle	Tranzition zone	2021/12/26	Winter	172	14.5	8.17	9.28	13.33	420	207	296	0.022	1.521	0.095	1.722	0.055	470945	147944226	314.14
W6-1	FD	Fengdu	107.7204	29.871405	431048	Middle	Tranzition zone	2021/12/27	Winter	172	14.1	8.22	9.44	16.67	413	198	291	0.025	1.525	0.055	1.691	0.092	500069	156951812	313.86
W6-2	FD	Fengdu	107.7204	29.871405	431048	Middle	Tranzition zone	2021/12/27	Winter	172	14.2	8.25	9.29	16.67	415	196	289	0.022	1.543	0.064	1.680	0.083	512015	160581405	313.63
W6-3	FD	Fengdu	107.7204	29.871405	431048	Middle	Tranzition zone	2021/12/27	Winter	172	14.6	8.19	9.04	16.67	415	196	290	0.025	1.552	0.073	1.722	0.074	536849	168553593	313.97
W7-1	ZX	Zhongxian	107.9977	30.26792	372779	Middle	Tranzition zone	2021/12/27	Winter	172	14.3	8.20	9.41	23.33	409	193	284	0.044	1.517	0.028	1.701	0.022	500991	157318514	314.01
W7-2	ZX	Zhongxian	107.9977	30.26792	372779	Middle	Tranzition zone	2021/12/27	Winter	172	14.2	8.21	9.20	23.33	410	193	294	0.045	1.597	0.015	1.629	0.015	514797	161538484	313.79
W7-3	ZX	Zhongxian	107.9977	30.26792	372779	Middle	Tranzition zone	2021/12/27	Winter	172	14.6	8.17	9.05	23.33	406	200	287	0.046	1.508	0.022	1.629	0.015	740974	232878147	314.29
W8-1	WZ	Wanzhou	108.3895	30.794857	283706	Middle	Tranzition zone	2021/12/27	Winter	172	13.9	7.98	9.57	25.00	401	184	284	0.013	1.543	0.032	1.619	0.062	578005	181193980	313.48
W8-2	WZ	Wanzhou	108.3895	30.794857	283706	Middle	Tranzition zone	2021/12/27	Winter	172	14.6	8.06	9.22	25.00	390	186	274	0.013	1.570	0.067	1.742	0.086	721801	226231486	313.43
W8-3	WZ	Wanzhou	108.3895	30.794857	283706	Middle	Tranzition zone	2021/12/27	Winter	172	14.6	8.07	9.23	25.00	399	186	274	0.013	1.588	0.068	1.639	0.077	652857	204652076	313.47
W9-1	YY	Yunyang	108.7199	30.920005	241557	Lower	Tranzition zone	2021/12/28	Winter	172	14.3	8.13	9.27	16.67	387	182	270	0.005	1.530	0.050	1.608	0.031	709886	222465763	313.38
W9-2	YY	Yunyang	108.7199	30.920005	241557	Lower	Tranzition zone	2021/12/28	Winter	172	14.3	8.14	8.96	16.67	387	185	271	0.005	1.521	0.047	1.546	0.037	676402	211873873	313.24
W9-3	YY	Yunyang	108.7199	30.920005	241557	Lower	Tranzition zone	2021/12/28	Winter	172	14.4	8.11	9.08	16.67	392	180	270	0.004	1.534	0.043	1.608	0.034	580502	181841303	313.25
W10-1	FJ	Fengjie	109.5051	31.03669	162083	Lower	Lacustrine zone	2021/12/28	Winter	172	15.4	8.06	8.92	11.67	399	188	280	0.014	1.588	0.142	1.918	0.197	733467	229727206	313.21
W10-2	FJ	Fengjie	109.5051	31.03669	162083	Lower	Lacustrine zone	2021/12/28	Winter	172	14.8	8.07	9.01	11.67	396	190	277	0.016	1.552	0.118	1.866	0.200	675156	211693535	313.55
W10-3	FJ	Fengjie	109.5051	31.03669	162083	Lower	Lacustrine zone	2021/12/28	Winter	172	14.6	8.07	9.16	11.67	396	187	275	0.014	1.530	0.117	1.649	0.215	748540	234458041	313.22
W11-1	WS	Wushan	109.9171	31.059443	119292	Lower	Lacustrine zone	2021/12/29	Winter	172	16.5	8.18	9.88	16.67	387	183	277	0.009	1.512	0.073	1.598	0.034	684161	214281005	313.2
W11-2	WS	Wushan	109.9171	31.059443	119292	Lower	Lacustrine zone	2021/12/29	Winter	172	15.7	8.20	9.80	16.67	385	186	270	0.008	1.517	0.042	1.629	0.022	504964	158147115	313.18
W11-3	WS	Wushan	109.9171	31.059443	119292	Lower	Lacustrine zone	2021/12/29	Winter	172	15.6	8.17	9.80	16.67	387	183	270	0.006	1.539	0.038	1.598	0.037	649457	203367517	313.13
W12-1	BD	Badong	110.3321	31.047456	75183	Lower	Lacustrine zone	2021/12/28	Winter	172	15.1	8.20	9.23	13.33	396	186	274	0.007	1.521	0.042	1.619	0.012	618766	193905431	313.37
W12-2	BD	Badong	110.3321	31.047456	75183	Lower	Lacustrine zone	2021/12/28	Winter	172	15.2	8.16	9.15	13.33	390	182	268	0.008	1.494	0.013	1.608	0.009	518105	162160549	312.99
W12-3	BD	Badong	110.3321	31.047456	75183	Lower	Lacustrine zone	2021/12/28	Winter	172	15.0	8.15	9.19	13.33	389	183	276	0.009	1.517	0.012	1.629	0.011	552996	173385225	313.54
W13-1	ZG	Zigui	110.9735	30.845309	3685	Lower	Lacustrine zone	2021/12/29	Winter	172	15.6	8.15	9.28	13.33	391	187	275	0.009	1.490	0.070	1.588	0.166	520014	162937863	313.33
W13-2	ZG	Zigui	110.9735	30.845309	3685	Lower	Lacustrine zone	2021/12/29	Winter	172	14.9	8.16	9.28	13.33	393	186	274	0.008	1.503	0.043	1.619	0.142	550879	172605517	313.33
W13-3	ZG	Zigui	110.9735	30.845309	3685	Lower	Lacustrine zone	2021/12/29	Winter	172	15.8	8.24	9.28	13.33	397	185	273	0.009	1.561	0.057	1.536	0.188	386622	121116206	313.27
W14-1	YL	Yiling	111.0823	30.856624	-9035	Lower	Lacustrine zone	2021/12/29	Winter	172	14.0	8.24	9.10	18.33	393	180	275	0.009	1.485	0.042	1.619	0.065	541435	169727217	313.48
W14-2	YL	Yiling	111.0823	30.856624	-9035	Lower	Lacustrine zone	2021/12/29	Winter	172	15.0	8.26	9.14	18.33	395	189	277	0.009	1.490	0.018	1.814	0.049	617936	193596511	313.3
W14-3	YL	Yiling	111.0823	30.856624	-9035	Lower	Lacustrine zone	2021/12/29	Winter	172	15.3	8.21	9.00	18.33	403	184	276	0.009	1.476	0.032	1.742	0.046	591188	185203003	313.27

Notice: T, water temperature; DO, dissolved oxygen; COD, chemical oxygen demand; EC, electrical conductivity; TDS, total dissolved solids; NO_2-N, nitrite nitrogen; NO_3-N, nitrate nitrogen; NH_4-N, ammonium nitrogen; TN, total nitrogen; TP, total phosphorus.

Table S2. Comparison of environmental variables between summer and winter

**Environmental variables**	**Summer (n=42)**		**Winter (n=42)**		***p***
	**Mean**	**SE**	**Mean**	**SE**	
T (℃)	**28.26**	**0.28**	**14.60**	**0.10**	**0.000**
pH	**7.89**	**0.02**	**8.19**	**0.01**	**0.000**
DO (mg/L)	**7.36**	**0.04**	**9.31**	**0.04**	**0.000**
COD (mg/L)	15.92	0.86	16.67	0.66	0.228
EC (μS/cm)	**379.31**	**1.88**	**405.52**	**2.12**	**0.000**
Salinity (mg/L)	**179.62**	**0.97**	**192.19**	**1.26**	**0.000**
TDS (mg/L)	**264.45**	**1.40**	**283.67**	**1.59**	**0.000**
NO_2-N (mg/L)	**0.020**	**0.00**	**0.015**	**0.00**	**0.003**
NO_3-N (mg/L)	**1.345**	**0.03**	**1.533**	**0.01**	**0.000**
NH_4-N (mg/L)	**0.323**	**0.02**	**0.063**	**0.01**	**0.000**
TN (mg/L)	1.754	0.02	1.689	0.01	0.114
TP (mg/L)	**0.084**	**0.01**	**0.060**	**0.01**	**0.000**

Table S3. Annotation results of representative sequences under different similarity thresholds at the species level

Similarity thresholds	97%	95%	90%	80%
Classified ASVs	3394	1985	4346	3768
Remaining unclassified ASVs	14273	12288	7942	4174
Cumulative classified ASVs	3394	5379	9725	13493

Table S4. The ASV richness, and Shannon index of meio- and microeukaryotic, zooplankton, and zoobenthos communities in the 84 samples

Taxa	Meio- and microeukaryotes (All ASVs)		Zooplankton		Zoobenthos	
SampleID	ASV richness	Shannon	ASV richness	Shannon	ASV richness	Shannon
S1-1	4570	8.7	1466	7.0	487	5.2
S1-2	5144	9.1	1645	7.5	592	5.8
S1-3	4612	9.0	1487	7.3	480	6.3
S2-1	4884	8.9	1574	7.4	578	4.8
S2-2	6912	9.5	1967	7.7	743	5.3
S2-3	5902	9.2	1748	7.5	613	5.2
S3-1	4430	8.5	1520	7.1	492	4.4
S3-2	5286	8.7	1566	7.4	596	4.2
S3-3	4387	8.6	1442	7.4	486	4.6
S4-1	4556	8.8	1539	7.5	513	4.0
S4-2	3900	8.2	1333	7.0	481	3.7
S4-3	3945	8.7	1366	7.4	450	4.5
S5-1	2978	7.7	1028	6.6	337	4.9
S5-2	4843	8.4	1403	7.0	532	5.3
S5-3	4596	8.8	1417	7.3	514	5.2
S6-1	3559	8.2	1315	7.0	443	4.5
S6-2	4410	8.5	1458	7.1	550	4.6
S6-3	3885	8.2	1365	6.7	498	4.2
S7-1	2803	7.5	971	5.5	261	5.6
S7-2	1607	6.0	541	5.7	196	5.1
S7-3	2320	7.2	709	6.2	209	5.5
S8-1	3418	8.2	1084	6.4	353	5.1
S8-2	2870	7.7	883	6.5	280	4.3
S8-3	3641	8.2	1094	6.1	359	5.3
S9-1	2653	8.0	737	5.9	210	4.9
S9-2	3928	8.1	959	6.4	282	5.3
S9-3	3613	8.8	942	6.9	275	5.5
S10-1	3338	8.1	674	6.5	206	4.8
S10-2	3417	7.8	608	5.8	209	5.2
S10-3	3396	8.2	742	6.0	227	5.2
S11-1	2931	6.2	802	4.0	235	5.5
S11-2	3045	7.8	767	5.6	234	5.2
S11-3	3902	7.7	1027	5.3	316	5.9
S12-1	2982	7.7	804	5.4	247	5.0
S12-2	3592	8.4	808	5.9	270	5.4
S12-3	3772	8.1	885	5.7	275	5.6
S13-1	3027	6.7	454	6.1	148	5.0
S13-2	1860	6.0	277	4.9	103	4.6
S13-3	2497	6.2	361	5.9	104	4.3
S14-1	1741	5.9	296	4.1	134	2.6
S14-2	2067	6.0	354	3.1	92	4.5
S14-3	2014	6.2	364	5.3	94	3.9
W1-1	5383	8.2	1016	5.2	512	4.0
W1-2	5403	8.1	964	4.7	456	4.0
W1-3	5391	8.2	1012	5.0	459	3.8
W2-1	5951	8.2	970	5.0	474	3.9
W2-2	6201	8.4	1001	5.2	514	4.0
W2-3	6308	8.5	1107	5.4	529	4.0
W3-1	5857	8.4	1005	5.6	554	4.9
W3-2	6145	8.4	1036	5.2	533	4.1
W3-3	6271	8.5	1150	5.7	589	4.1

W4-1	6125	8.4	816	4.9	432	5.2
W4-2	6032	8.3	753	4.7	435	5.4
W4-3	5944	8.3	782	4.8	394	5.2
W5-1	5343	7.9	717	4.1	425	6.4
W5-2	5678	8.1	774	4.2	461	6.4
W5-3	5433	7.9	713	3.9	436	5.5
W6-1	4430	7.6	498	3.5	295	5.8
W6-2	5023	7.4	572	3.4	309	4.8
W6-3	4624	7.5	516	3.3	304	6.0
W7-1	4867	7.6	494	3.5	233	5.7
W7-2	4456	6.6	533	3.1	197	5.6
W7-3	5013	8.0	445	3.3	275	5.7
W8-1	4330	7.6	372	2.9	197	5.3
W8-2	4627	7.6	399	2.7	242	5.7
W8-3	4549	7.7	367	2.9	227	5.8
W9-1	4489	7.8	439	3.1	257	4.5
W9-2	4379	7.7	419	3.0	205	5.3
W9-3	4402	7.4	376	2.8	220	5.6
W10-1	4268	7.0	581	4.0	215	5.6
W10-2	4128	7.3	401	4.7	238	5.5
W10-3	4856	7.9	489	4.8	239	5.6
W11-1	4079	7.4	361	4.6	178	5.2
W11-2	3414	7.2	430	4.2	159	4.2
W11-3	4148	7.5	404	4.9	212	5.0
W12-1	3374	7.1	351	4.6	139	3.9
W12-2	4303	7.2	445	5.2	218	4.6
W12-3	3547	6.6	338	5.0	177	5.2
W13-1	3973	7.7	362	4.5	179	5.2
W13-2	3952	7.6	340	4.2	141	4.9
W13-3	3793	7.6	332	4.5	166	4.2
W14-1	3943	7.7	377	4.3	152	5.1
W14-2	3879	7.5	362	4.3	167	5.2
W14-3	4126	7.4	402	4.2	202	5.2

Table S5. PCNM vector based on the longitude and latitude coordinates of 84 sampling sites for two seasons

SampleID	PCNM1	PCNM2	PCNM3	PCNM4	PCNM5	PCNM6	PCNM7	PCNM8	PCNM9	PCNM10
S1-1	0.0714	-0.1530	0.0530	0.0696	0.1594	-0.1939	0.1637	0.0041	0.0133	-0.0172
S1-2	0.0714	-0.1530	0.0530	0.0696	0.1594	-0.1939	0.1637	0.0041	0.0133	-0.0172
S1-3	0.0714	-0.1530	0.0530	0.0696	0.1594	-0.1939	0.1637	0.0041	0.0133	-0.0172
S2-1	0.1139	-0.1617	0.0289	0.0221	0.0050	0.0486	-0.0575	0.0215	-0.2814	0.0850
S2-2	0.1139	-0.1617	0.0289	0.0221	0.0050	0.0486	-0.0575	0.0215	-0.2814	0.0850
S2-3	0.1139	-0.1617	0.0289	0.0221	0.0050	0.0486	-0.0575	0.0215	-0.2814	0.0850
S3-1	0.1142	-0.1612	0.0282	0.0209	0.0008	0.0564	-0.1023	-0.0254	0.2714	-0.0679
S3-2	0.1142	-0.1612	0.0282	0.0209	0.0008	0.0564	-0.1023	-0.0254	0.2714	-0.0679
S3-3	0.1142	-0.1612	0.0282	0.0209	0.0008	0.0564	-0.1023	-0.0254	0.2714	-0.0679
S4-1	0.1511	-0.0227	-0.0654	-0.0904	-0.1725	0.1465	-0.0049	0.0005	-0.0034	0.0002
S4-2	0.1511	-0.0227	-0.0654	-0.0904	-0.1725	0.1465	-0.0049	0.0005	-0.0034	0.0002
S4-3	0.1511	-0.0227	-0.0654	-0.0904	-0.1725	0.1465	-0.0049	0.0005	-0.0034	0.0002
S5-1	0.1041	0.1364	-0.0962	-0.0688	-0.0177	-0.0661	0.1050	-0.2786	-0.0048	0.0374
S5-2	0.1041	0.1364	-0.0962	-0.0688	-0.0177	-0.0661	0.1050	-0.2786	-0.0048	0.0374
S5-3	0.1041	0.1364	-0.0962	-0.0688	-0.0177	-0.0661	0.1050	-0.2786	-0.0048	0.0374
S6-1	0.1035	0.1375	-0.0957	-0.0670	-0.0117	-0.0718	0.0575	0.2890	0.0143	-0.0540
S6-2	0.1035	0.1375	-0.0957	-0.0670	-0.0117	-0.0718	0.0575	0.2890	0.0143	-0.0540
S6-3	0.1035	0.1375	-0.0957	-0.0670	-0.0117	-0.0718	0.0575	0.2890	0.0143	-0.0540
S7-1	0.0551	0.1769	-0.0210	0.0868	0.1790	-0.0215	-0.1595	-0.0107	-0.0067	0.0175
S7-2	0.0551	0.1769	-0.0210	0.0868	0.1790	-0.0215	-0.1595	-0.0107	-0.0067	0.0175
S7-3	0.0551	0.1769	-0.0210	0.0868	0.1790	-0.0215	-0.1595	-0.0107	-0.0067	0.0175

S8-1	-0.0195	0.1092	0.1305	0.1971	0.0375	0.1760	-0.0064	-0.0035	-0.0031	-0.0003
S8-2	-0.0195	0.1092	0.1305	0.1971	0.0375	0.1760	-0.0064	-0.0035	-0.0031	-0.0003
S8-3	-0.0195	0.1092	0.1305	0.1971	0.0375	0.1760	-0.0064	-0.0035	-0.0031	-0.0003
S9-1	-0.0574	0.0606	0.2005	0.0658	-0.1769	-0.0243	0.1587	0.0144	0.0092	-0.0160
S9-2	-0.0574	0.0606	0.2005	0.0658	-0.1769	-0.0243	0.1587	0.0144	0.0092	-0.0160
S9-3	-0.0574	0.0606	0.2005	0.0658	-0.1769	-0.0243	0.1587	0.0144	0.0092	-0.0160
S10-1	-0.0852	0.0205	0.1701	-0.1662	-0.0645	-0.1481	-0.1539	-0.0110	-0.0069	0.0179
S10-2	-0.0852	0.0205	0.1701	-0.1662	-0.0645	-0.1481	-0.1539	-0.0110	-0.0069	0.0179
S10-3	-0.0852	0.0205	0.1701	-0.1662	-0.0645	-0.1481	-0.1539	-0.0110	-0.0069	0.0179
S11-1	-0.1166	-0.0128	0.0533	-0.2183	0.1547	0.1085	-0.0040	-0.0019	-0.0021	-0.0024
S11-2	-0.1166	-0.0128	0.0533	-0.2183	0.1547	0.1085	-0.0040	-0.0019	-0.0021	-0.0024
S11-3	-0.1166	-0.0128	0.0533	-0.2183	0.1547	0.1085	-0.0040	-0.0019	-0.0021	-0.0024
S12-1	-0.1657	-0.0455	-0.1130	-0.0330	0.0698	0.1301	0.1571	0.0136	0.0087	-0.0149
S12-2	-0.1657	-0.0455	-0.1130	-0.0330	0.0698	0.1301	0.1571	0.0136	0.0087	-0.0149
S12-3	-0.1657	-0.0455	-0.1130	-0.0330	0.0698	0.1301	0.1571	0.0136	0.0087	-0.0149
S13-1	-0.1348	-0.0422	-0.1369	0.0905	-0.0810	-0.0686	-0.0558	0.0368	0.0765	0.2803
S13-2	-0.1348	-0.0422	-0.1369	0.0905	-0.0810	-0.0686	-0.0558	0.0368	0.0765	0.2803
S13-3	-0.1348	-0.0422	-0.1369	0.0905	-0.0810	-0.0686	-0.0558	0.0368	0.0765	0.2803
S14-1	-0.1341	-0.0420	-0.1363	0.0908	-0.0819	-0.0717	-0.0978	-0.0491	-0.0851	-0.2656
S14-2	-0.1341	-0.0420	-0.1363	0.0908	-0.0819	-0.0717	-0.0978	-0.0491	-0.0851	-0.2656
S14-3	-0.1341	-0.0420	-0.1363	0.0908	-0.0819	-0.0717	-0.0978	-0.0491	-0.0851	-0.2656
W1-1	0.0714	-0.1530	0.0530	0.0696	0.1594	-0.1939	0.1637	0.0041	0.0133	-0.0172
W1-2	0.0714	-0.1530	0.0530	0.0696	0.1594	-0.1939	0.1637	0.0041	0.0133	-0.0172

W1-3	0.0714	-0.1530	0.0530	0.0696	0.1594	-0.1939	0.1637	0.0041	0.0133	-0.0172
W2-1	0.1139	-0.1617	0.0289	0.0221	0.0050	0.0486	-0.0575	0.0215	-0.2814	0.0850
W2-2	0.1139	-0.1617	0.0289	0.0221	0.0050	0.0486	-0.0575	0.0215	-0.2814	0.0850
W2-3	0.1139	-0.1617	0.0289	0.0221	0.0050	0.0486	-0.0575	0.0215	-0.2814	0.0850
W3-1	0.1142	-0.1612	0.0282	0.0209	0.0008	0.0564	-0.1023	-0.0254	0.2714	-0.0679
W3-2	0.1142	-0.1612	0.0282	0.0209	0.0008	0.0564	-0.1023	-0.0254	0.2714	-0.0679
W3-3	0.1142	-0.1612	0.0282	0.0209	0.0008	0.0564	-0.1023	-0.0254	0.2714	-0.0679
W4-1	0.1511	-0.0227	-0.0654	-0.0904	-0.1725	0.1465	-0.0049	0.0005	-0.0034	0.0002
W4-2	0.1511	-0.0227	-0.0654	-0.0904	-0.1725	0.1465	-0.0049	0.0005	-0.0034	0.0002
W4-3	0.1511	-0.0227	-0.0654	-0.0904	-0.1725	0.1465	-0.0049	0.0005	-0.0034	0.0002
W5-1	0.1041	0.1364	-0.0962	-0.0688	-0.0177	-0.0661	0.1050	-0.2786	-0.0048	0.0374
W5-2	0.1041	0.1364	-0.0962	-0.0688	-0.0177	-0.0661	0.1050	-0.2786	-0.0048	0.0374
W5-3	0.1041	0.1364	-0.0962	-0.0688	-0.0177	-0.0661	0.1050	-0.2786	-0.0048	0.0374
W6-1	0.1035	0.1375	-0.0957	-0.0670	-0.0117	-0.0718	0.0575	0.2890	0.0143	-0.0540
W6-2	0.1035	0.1375	-0.0957	-0.0670	-0.0117	-0.0718	0.0575	0.2890	0.0143	-0.0540
W6-3	0.1035	0.1375	-0.0957	-0.0670	-0.0117	-0.0718	0.0575	0.2890	0.0143	-0.0540
W7-1	0.0551	0.1769	-0.0210	0.0868	0.1790	-0.0215	-0.1595	-0.0107	-0.0067	0.0175
W7-2	0.0551	0.1769	-0.0210	0.0868	0.1790	-0.0215	-0.1595	-0.0107	-0.0067	0.0175
W7-3	0.0551	0.1769	-0.0210	0.0868	0.1790	-0.0215	-0.1595	-0.0107	-0.0067	0.0175
W8-1	-0.0195	0.1092	0.1305	0.1971	0.0375	0.1760	-0.0064	-0.0035	-0.0031	-0.0003
W8-2	-0.0195	0.1092	0.1305	0.1971	0.0375	0.1760	-0.0064	-0.0035	-0.0031	-0.0003
W8-3	-0.0195	0.1092	0.1305	0.1971	0.0375	0.1760	-0.0064	-0.0035	-0.0031	-0.0003
W9-1	-0.0574	0.0606	0.2005	0.0658	-0.1769	-0.0243	0.1587	0.0144	0.0092	-0.0160

W9-2	-0.0574	0.0606	0.2005	0.0658	-0.1769	-0.0243	0.1587	0.0144	0.0092	-0.0160
W9-3	-0.0574	0.0606	0.2005	0.0658	-0.1769	-0.0243	0.1587	0.0144	0.0092	-0.0160
W10-1	-0.0852	0.0205	0.1701	-0.1662	-0.0645	-0.1481	-0.1539	-0.0110	-0.0069	0.0179
W10-2	-0.0852	0.0205	0.1701	-0.1662	-0.0645	-0.1481	-0.1539	-0.0110	-0.0069	0.0179
W10-3	-0.0852	0.0205	0.1701	-0.1662	-0.0645	-0.1481	-0.1539	-0.0110	-0.0069	0.0179
W11-1	-0.1166	-0.0128	0.0533	-0.2183	0.1547	0.1085	-0.0040	-0.0019	-0.0021	-0.0024
W11-2	-0.1166	-0.0128	0.0533	-0.2183	0.1547	0.1085	-0.0040	-0.0019	-0.0021	-0.0024
W11-3	-0.1166	-0.0128	0.0533	-0.2183	0.1547	0.1085	-0.0040	-0.0019	-0.0021	-0.0024
W12-1	-0.1657	-0.0455	-0.1130	-0.0330	0.0698	0.1301	0.1571	0.0136	0.0087	-0.0149
W12-2	-0.1657	-0.0455	-0.1130	-0.0330	0.0698	0.1301	0.1571	0.0136	0.0087	-0.0149
W12-3	-0.1657	-0.0455	-0.1130	-0.0330	0.0698	0.1301	0.1571	0.0136	0.0087	-0.0149
W13-1	-0.1348	-0.0422	-0.1369	0.0905	-0.0810	-0.0686	-0.0558	0.0368	0.0765	0.2803
W13-2	-0.1348	-0.0422	-0.1369	0.0905	-0.0810	-0.0686	-0.0558	0.0368	0.0765	0.2803
W13-3	-0.1348	-0.0422	-0.1369	0.0905	-0.0810	-0.0686	-0.0558	0.0368	0.0765	0.2803
W14-1	-0.1341	-0.0420	-0.1363	0.0908	-0.0819	-0.0717	-0.0978	-0.0491	-0.0851	-0.2656
W14-2	-0.1341	-0.0420	-0.1363	0.0908	-0.0819	-0.0717	-0.0978	-0.0491	-0.0851	-0.2656
W14-3	-0.1341	-0.0420	-0.1363	0.0908	-0.0819	-0.0717	-0.0978	-0.0491	-0.0851	-0.2656

Notice: PCNM, Principal Coordinates of Neighborhood Matrices.

Table S6. PCNM vector based on the longitude and latitude coordinates of 42 sampling sites for a single season

SampleID	PCNM1	PCNM2	PCNM3	PCNM4	PCNM5	PCNM6	PCNM7	PCNM8	PCNM9	PCNM10
1-1	0.1010	-0.2164	0.0749	0.0984	0.2255	0.2743	0.2315	0.0059	0.0188	-0.0243
1-2	0.1010	-0.2164	0.0749	0.0984	0.2255	0.2743	0.2315	0.0059	0.0188	-0.0243
1-3	0.1010	-0.2164	0.0749	0.0984	0.2255	0.2743	0.2315	0.0059	0.0188	-0.0243
2-1	0.1611	-0.2287	0.0408	0.0312	0.0070	-0.0687	-0.0814	0.0304	-0.3980	0.1202
2-2	0.1611	-0.2287	0.0408	0.0312	0.0070	-0.0687	-0.0814	0.0304	-0.3980	0.1202
2-3	0.1611	-0.2287	0.0408	0.0312	0.0070	-0.0687	-0.0814	0.0304	-0.3980	0.1202
3-1	0.1616	-0.2279	0.0399	0.0295	0.0011	-0.0797	-0.1447	-0.0359	0.3838	-0.0961
3-2	0.1616	-0.2279	0.0399	0.0295	0.0011	-0.0797	-0.1447	-0.0359	0.3838	-0.0961
3-3	0.1616	-0.2279	0.0399	0.0295	0.0011	-0.0797	-0.1447	-0.0359	0.3838	-0.0961
4-1	0.2136	-0.0321	-0.0925	-0.1279	-0.2440	-0.2072	-0.0069	0.0008	-0.0048	0.0003
4-2	0.2136	-0.0321	-0.0925	-0.1279	-0.2440	-0.2072	-0.0069	0.0008	-0.0048	0.0003
4-3	0.2136	-0.0321	-0.0925	-0.1279	-0.2440	-0.2072	-0.0069	0.0008	-0.0048	0.0003
5-1	0.1473	0.1930	-0.1360	-0.0972	-0.0250	0.0935	0.1485	-0.3939	-0.0068	0.0529
5-2	0.1473	0.1930	-0.1360	-0.0972	-0.0250	0.0935	0.1485	-0.3939	-0.0068	0.0529
5-3	0.1473	0.1930	-0.1360	-0.0972	-0.0250	0.0935	0.1485	-0.3939	-0.0068	0.0529
6-1	0.1463	0.1945	-0.1354	-0.0947	-0.0166	0.1016	0.0813	0.4088	0.0202	-0.0763
6-2	0.1463	0.1945	-0.1354	-0.0947	-0.0166	0.1016	0.0813	0.4088	0.0202	-0.0763
6-3	0.1463	0.1945	-0.1354	-0.0947	-0.0166	0.1016	0.0813	0.4088	0.0202	-0.0763
7-1	0.0779	0.2502	-0.0298	0.1228	0.2532	0.0305	-0.2255	-0.0151	-0.0095	0.0247
7-2	0.0779	0.2502	-0.0298	0.1228	0.2532	0.0305	-0.2255	-0.0151	-0.0095	0.0247
7-3	0.0779	0.2502	-0.0298	0.1228	0.2532	0.0305	-0.2255	-0.0151	-0.0095	0.0247
8-1	-0.0275	0.1544	0.1846	0.2788	0.0531	-0.2489	-0.0091	-0.0049	-0.0043	-0.0004

8-2	-0.0275	0.1544	0.1846	0.2788	0.0531	-0.2489	-0.0091	-0.0049	-0.0043	-0.0004
8-3	-0.0275	0.1544	0.1846	0.2788	0.0531	-0.2489	-0.0091	-0.0049	-0.0043	-0.0004
9-1	-0.0812	0.0856	0.2835	0.0931	-0.2502	0.0343	0.2244	0.0204	0.0131	-0.0226
9-2	-0.0812	0.0856	0.2835	0.0931	-0.2502	0.0343	0.2244	0.0204	0.0131	-0.0226
9-3	-0.0812	0.0856	0.2835	0.0931	-0.2502	0.0343	0.2244	0.0204	0.0131	-0.0226
10-1	-0.1205	0.0290	0.2406	-0.2351	-0.0913	0.2094	-0.2176	-0.0155	-0.0098	0.0253
10-2	-0.1205	0.0290	0.2406	-0.2351	-0.0913	0.2094	-0.2176	-0.0155	-0.0098	0.0253
10-3	-0.1205	0.0290	0.2406	-0.2351	-0.0913	0.2094	-0.2176	-0.0155	-0.0098	0.0253
11-1	-0.1649	-0.0181	0.0754	-0.3087	0.2188	-0.1535	-0.0056	-0.0028	-0.0029	-0.0034
11-2	-0.1649	-0.0181	0.0754	-0.3087	0.2188	-0.1535	-0.0056	-0.0028	-0.0029	-0.0034
11-3	-0.1649	-0.0181	0.0754	-0.3087	0.2188	-0.1535	-0.0056	-0.0028	-0.0029	-0.0034
12-1	-0.2344	-0.0644	-0.1598	-0.0467	0.0988	-0.1840	0.2222	0.0193	0.0124	-0.0211
12-2	-0.2344	-0.0644	-0.1598	-0.0467	0.0988	-0.1840	0.2222	0.0193	0.0124	-0.0211
12-3	-0.2344	-0.0644	-0.1598	-0.0467	0.0988	-0.1840	0.2222	0.0193	0.0124	-0.0211
13-1	-0.1906	-0.0597	-0.1936	0.1280	-0.1145	0.0971	-0.0790	0.0521	0.1082	0.3964
13-2	-0.1906	-0.0597	-0.1936	0.1280	-0.1145	0.0971	-0.0790	0.0521	0.1082	0.3964
13-3	-0.1906	-0.0597	-0.1936	0.1280	-0.1145	0.0971	-0.0790	0.0521	0.1082	0.3964
14-1	-0.1896	-0.0594	-0.1927	0.1284	-0.1158	0.1014	-0.1384	-0.0694	-0.1204	-0.3756
14-2	-0.1896	-0.0594	-0.1927	0.1284	-0.1158	0.1014	-0.1384	-0.0694	-0.1204	-0.3756
14-3	-0.1896	-0.0594	-0.1927	0.1284	-0.1158	0.1014	-0.1384	-0.0694	-0.1204	-0.3756

Notice: PCNM, Principal Coordinates of Neighborhood Matrices.

Table S7. Forward selection results of environment and spatial (PCNM vector) variable for meio- and microeukaryotic zooplankton, and zoobenthos communities

Taxa	Environment variable			PCNM vector		
	Two seasons	Summer	Winter	Two seasons	Summer	Winter
Meio- and microeukaryotes	T, EC, NO2-N, NH4-N, NO3-N, TP, Salanity, COD, pH, TN	NO2-N, NO3-N, EC, T, NH4-N, DO, pH	EC, NO2-N, pH, COD, TP	PCNM (No. 1, 2)	PCNM (No. 1, 2, 3, 4, 5, 6, 7, 10)	PCNM (No. 1, 2, 3, 4, 5, 6, 7)
Zooplankton	T, EC, NH4-N, NO2-N, TP, Salinity, COD	NO2-N, T, EC, NH4-N, NO3-N	EC, NO2-N, DO, COD	PCNM (No. 1, 2)	PCNM (No. 1, 2, 3, 4, 5, 6, 7, 10)	PCNM (No. 1, 2, 3, 4, 5, 6, 7)
Zoobenthos	T, EC, NO2-N, NH4-N, TP, NO3-N, Salinity, COD, DO	NO2-N, T, EC, DO	EC, NO2-N, pH, Salinity, DO, COD	PCNM (No. 1, 2, 3)	PCNM (No. 1, 2, 3, 4, 5, 6, 7, 10)	PCNM (No. 1, 2, 3, 4, 5, 6, 7, 8)

Notice: T, water temperature; DO, dissolved oxygen; COD, chemical oxygen demand; EC, electrical conductivity; TDS, total dissolved solids; NO_2-N, nitrite nitrogen; NO_3-N, nitrate nitrogen; NH_4-N, ammonium nitrogen; TN, total nitrogen; TP, total phosphorus. PCNM, Principal Coordinates of Neighborhood Matrices.

Table S8. Species identification in this study.

Please see the Excel file **Table S8**.

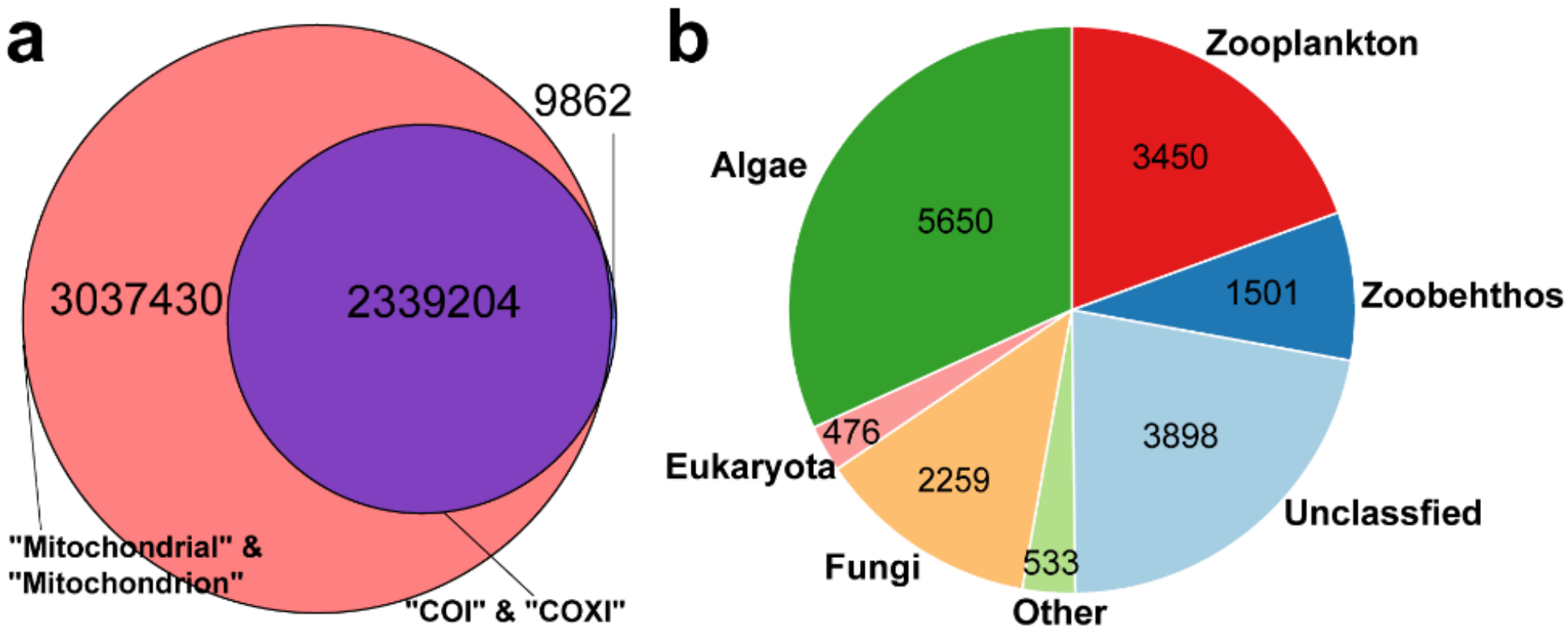

Fig. S1. Composition of the Mito-COI reference database and ASVs. **(a)** Composition of Mito-COI reference database based on four keywords searched in the NCBI. The number indicates the sequence number. **(b)** Composition of ASVs for different categories of taxa. The number indicates the ASV number of each taxon. Eukaryota, unclassified eukaryotes; Other, other identified species not classified as algae, zooplankton, zoobenthos, and fungi; Unclassified, unidentified ASVs.

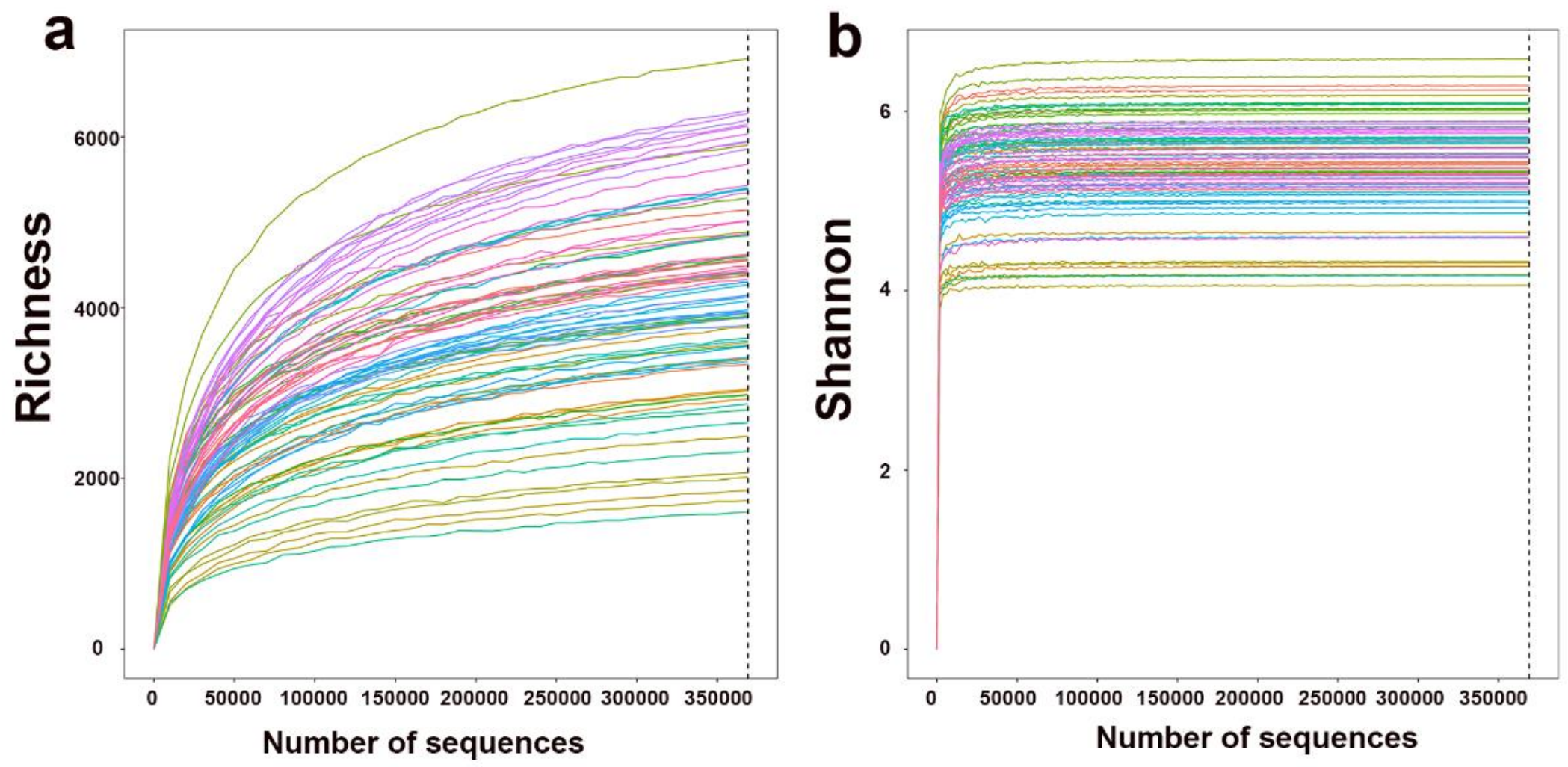

Fig. S2. Rarefaction curves of various samples. **(a)** Rarefaction curves of ASV richness for 84 samples. **(b)** Rarefaction curves of the Shannon index for 84 samples.

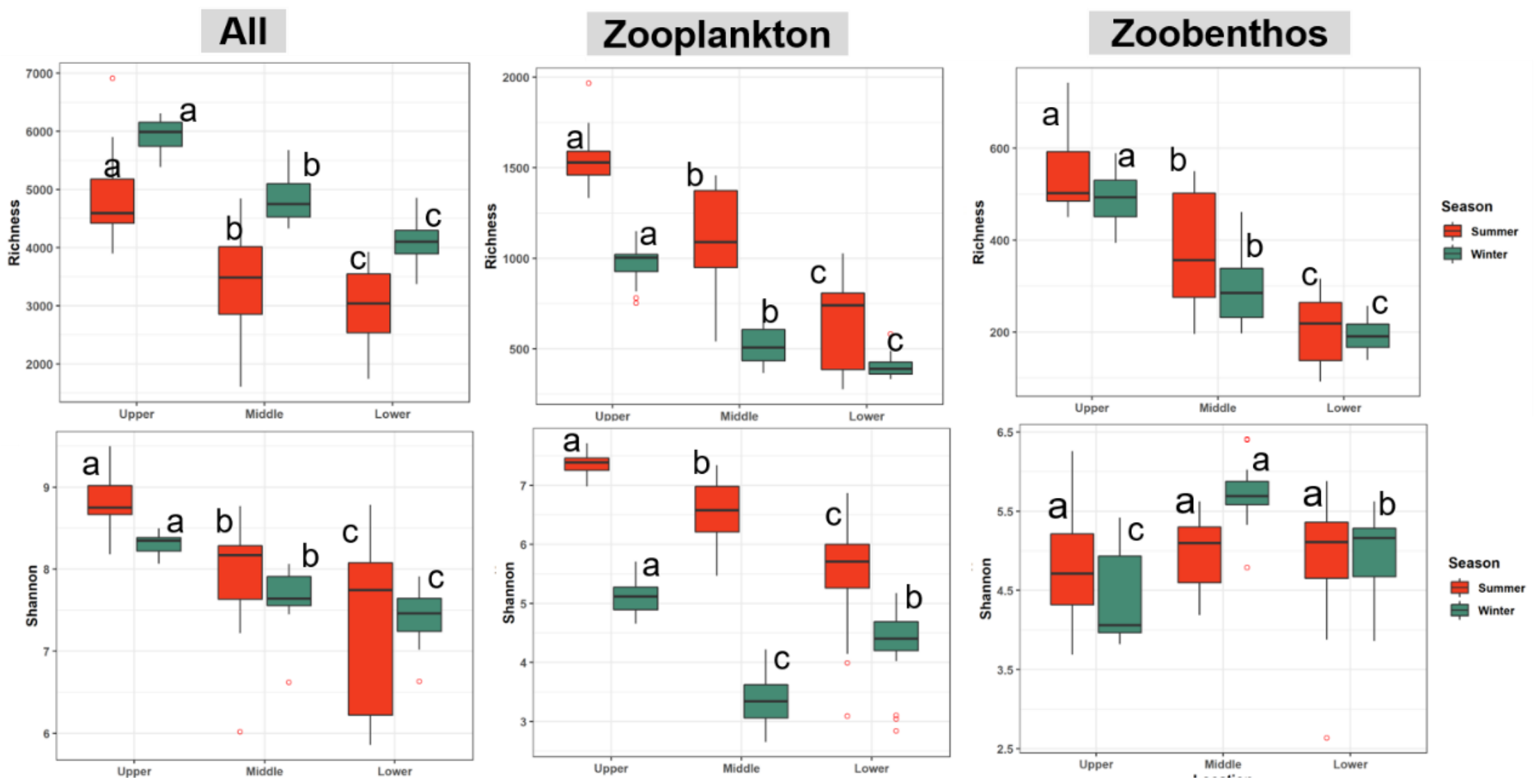

Fig. S3. Comparison of diversity indices for meio- and microeukaryotic, zooplankton, and zoobenthos communities among the three reaches in the summer and winter. Different letters above boxes indicate significant differences at the $p < 0.05$ level using analysis of one-way variance (ANOVA). All, all ASVs (meio- and micro-eukaryotic communities).

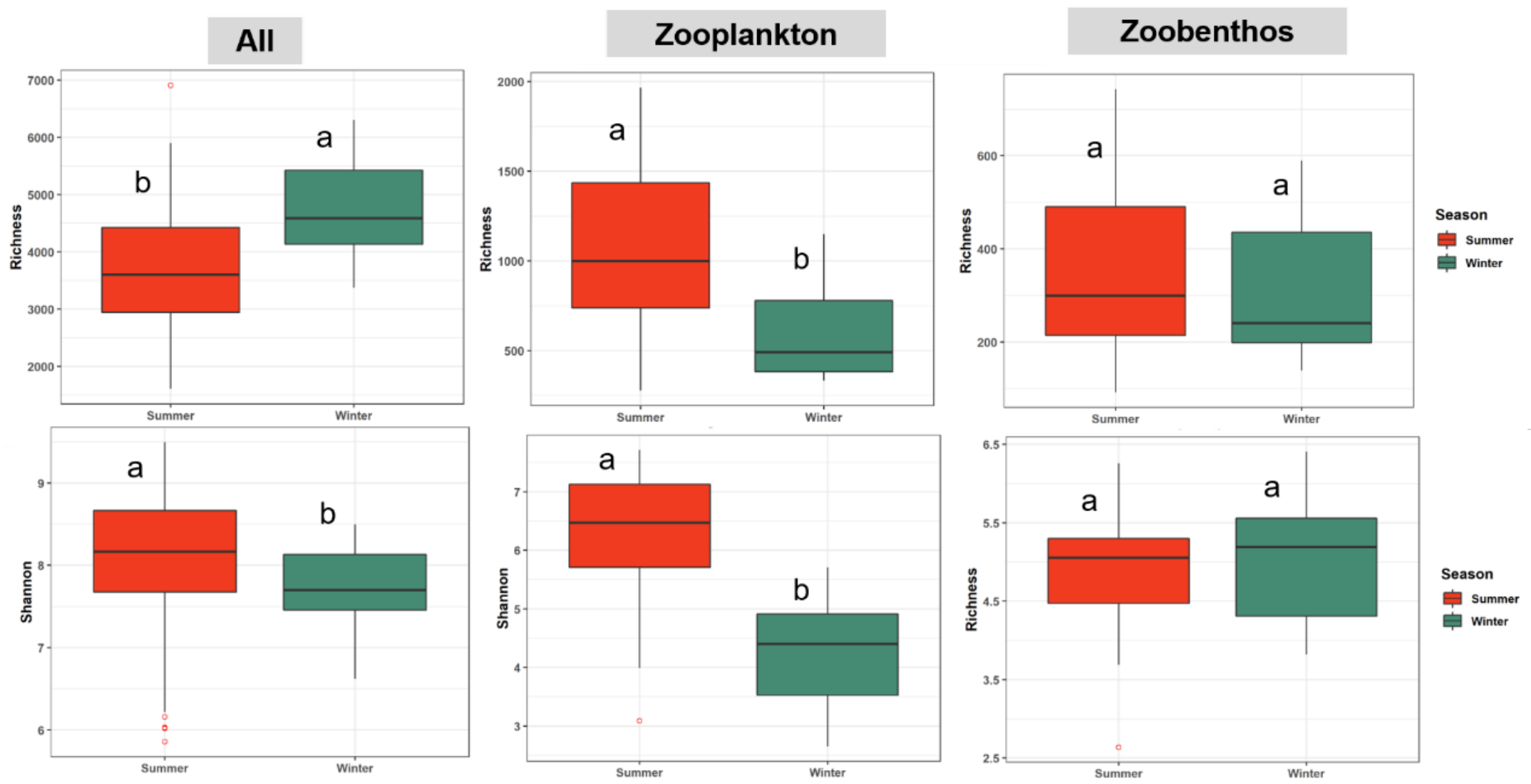

Fig. S4. Comparison of diversity indices for meio- and microeukaryotic, zooplankton, and zoobenthos communities between summer and winter. Different letters above boxes indicate significant differences at the $p < 0.05$ level using the Mann-Whitney *U* test. All, all ASVs (meio- and microeukaryotic community).

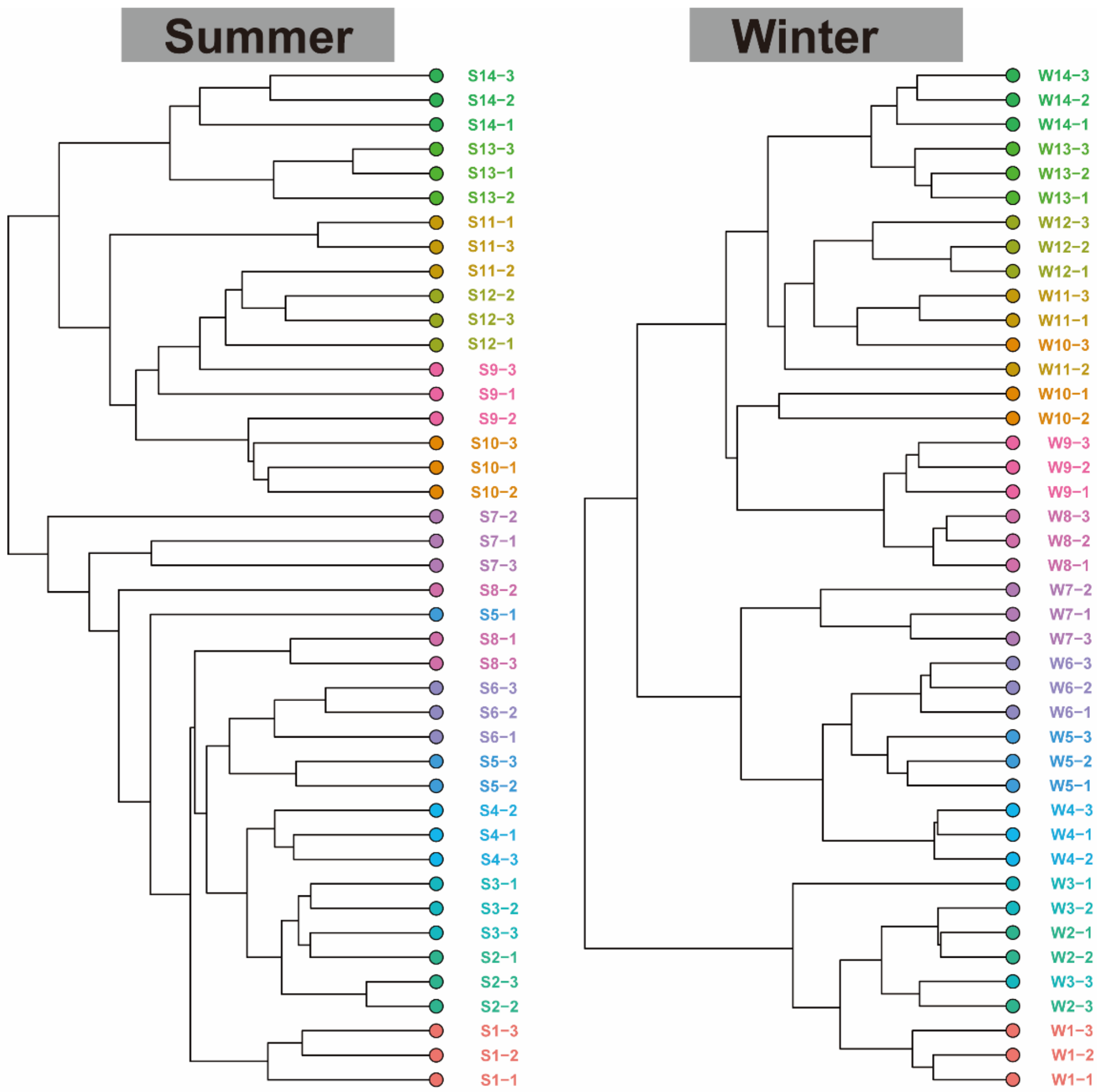

Fig. S5. The phylogenetic tree is based on the Bray-Curtis' similarity of meio- and microeukaryotes among 42 samples in summer and winter, respectively. Sample names are colored according to season (S and W) and sample sites (1-14) of sampling in three replicates (1-3) and details are provided in **Supplementary Table S1**.

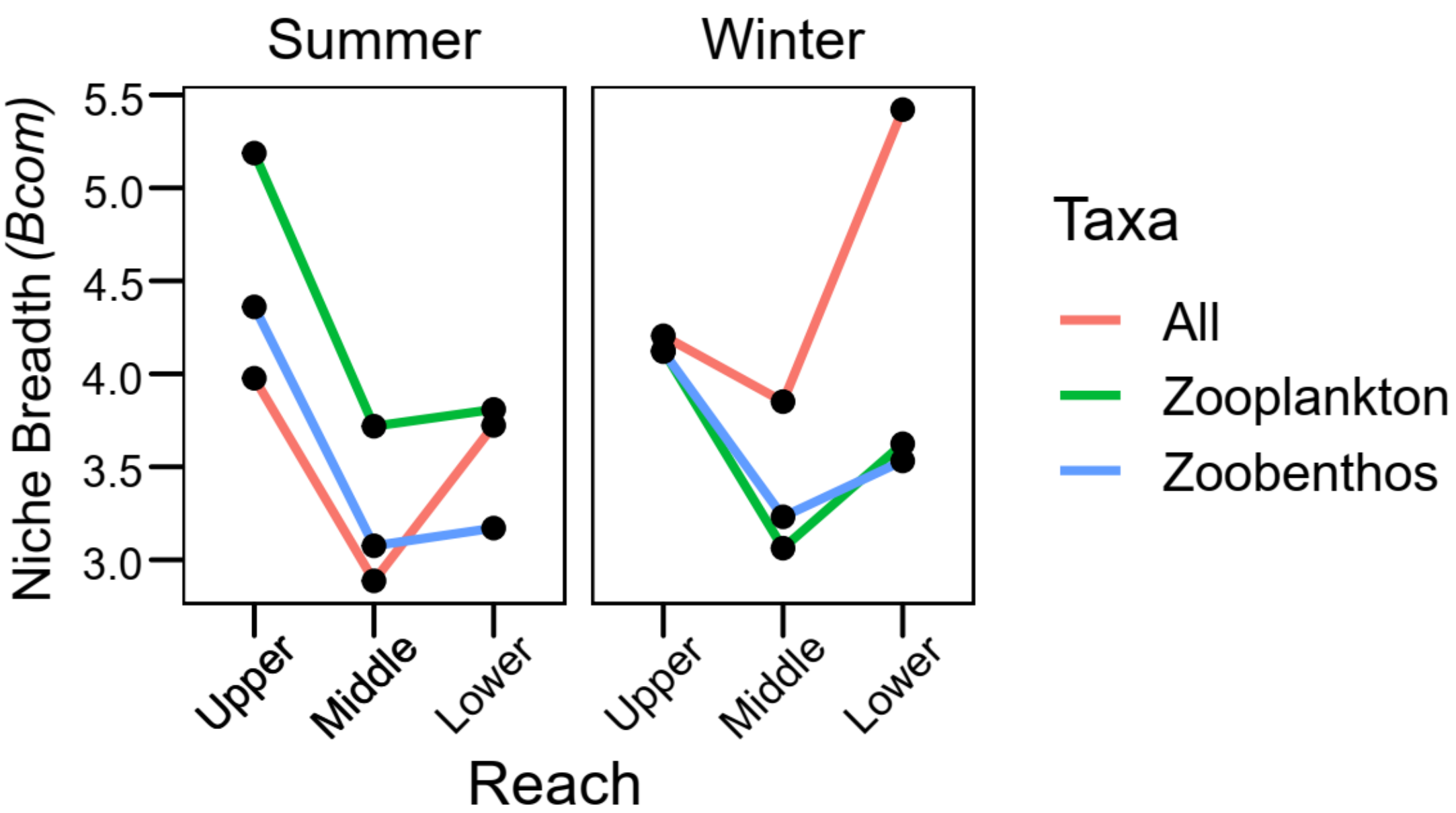

Fig. S6. Mean habitat niche breadth (*Bcom*) for meio- and microeukaryotes, zooplankton, and zoobenthos at three reaches of TGR in summer and winter. All, all ASVs (meio- and microeukaryotic community).
