# Supplementary material for: Biogeographic patterns of meio- and micro-eukaryotic communities in dam-induced river-reservoir systems
Source: Appl Microbiol Biotechnol. 2024 Jan 15;108(1):130. doi: 10.1007/s00253-023-12993-4 (PMC10789839; doi:10.1007/s00253-023-12993-4)
Supplement: Supplementary file 1 — Supplementary file1 (PDF 1035 KB) [file 253_2023_12993_MOESM1_ESM.pdf]

# Supplementary Information

## Biogeographic patterns of meio- and micro-eukaryotic communities in dam-induced river-reservoir systems

Huan Hu[1,2], Xing-Yi Wei[1,2], Li Liu[2], Yuan-Bo Wang[1,2], Ling-Kang Bu[2,3], Huang-Jie Jia[2], De-Sheng Pei[3*]

[1] Chongqing Jiaotong University, Chongqing 400074, China

[2] Chongqing Institute of Green and Intelligent Technology, Chongqing School of University of Chinese Academy of Sciences, Chinese Academy of Sciences, Chongqing 400714, China

[3] School of Public Health, Chongqing Medical University, Chongqing 400016, China

*Corresponding author

E-mail: peids@cqmu.edu.cn (D.S.P)

## Table S1. Summary of sampling, environmental variables, and sequences in the Three Gorges Reservoir (TGR) in the summer and winter

| | Sampling information | | | | | | | | | | Environmental variables | | | | | | | | | | | | Sequencing information | | |
|---|---|---|---|---|---|---|---|---|---|---|---|---|---|---|---|---|---|---|---|---|---|---|---|---|---|
| SampleID | Site | Location | Longitude | Latitude | Distance from dam | Reach | Water body | Sampling date | Season | Water level | T | pH | DO | COD | EC | Salinity | TDS | NO2-N | NO3-N | NH4-N | TN | TP | Sequencing depth | Bases | Average length |
| | | | (E) | (N) | (m) | | | | | (m) | (℃) | | (mg/L) | (mg/L) | μS/cm | (mg/L) | (mg/L) | (mg/L) | (mg/L) | (mg/L) | (mg/L) | (mg/L) | | (bp) | (bp) |
| S1-1 | JJ | Jiangjin | 106.2207 | 29.21483 | 687946 | Upper | Riverine zone | 2021/8/20 | Summer | 146.5 | 29.3 | 7.96 | 7.21 | 12.86 | 384 | 199 | 268 | 0.009 | 1.321 | 0.164 | 1.877 | 0.071 | 679890 | 212485821 | 312.53 |
| S1-2 | JJ | Jiangjin | 106.2207 | 29.21483 | 687946 | Upper | Riverine zone | 2021/8/20 | Summer | 146.5 | 28.4 | 7.95 | 7.21 | 11.43 | 383 | 183 | 268 | 0.015 | 1.572 | 0.191 | 1.929 | 0.129 | 560559 | 175228414 | 312.6 |
| S1-3 | JJ | Jiangjin | 106.2207 | 29.21483 | 687946 | Upper | Riverine zone | 2021/8/20 | Summer | 146.5 | 28.3 | 7.76 | 7.20 | 10.00 | 384 | 180 | 268 | 0.008 | 1.605 | 0.138 | 2.054 | 0.105 | 693233 | 216597918 | 312.45 |
| S2-1 | BN | Banan | 106.5426 | 29.473633 | 620510 | Upper | Riverine zone | 2021/8/20 | Summer | 146.5 | 28.1 | 7.99 | 7.26 | 10.00 | 385 | 187 | 277 | 0.024 | 1.216 | 0.112 | 1.617 | 0.047 | 674780 | 210906077 | 312.56 |
| S2-2 | BN | Banan | 106.5426 | 29.473633 | 620510 | Upper | Riverine zone | 2021/8/20 | Summer | 146.5 | 29.3 | 7.94 | 7.37 | 11.43 | 382 | 185 | 261 | 0.021 | 1.321 | 0.112 | 1.575 | 0.028 | 666077 | 208188857 | 312.56 |
| S2-3 | BN | Banan | 106.5426 | 29.473633 | 620510 | Upper | Riverine zone | 2021/8/20 | Summer | 146.5 | 27.6 | 8.00 | 7.61 | 8.57 | 396 | 180 | 272 | 0.014 | 1.362 | 0.112 | 1.711 | 0.035 | 657373 | 205471637 | 312.56 |
| S3-1 | NA | Nanan | 106.5841 | 29.602583 | 600876 | Upper | Riverine zone | 2021/8/20 | Summer | 146.5 | 27.1 | 7.95 | 7.76 | 14.29 | 417 | 190 | 287 | 0.015 | 1.216 | 0.224 | 1.586 | 0.148 | 656492 | 205219110 | 312.6 |
| S3-2 | NA | Nanan | 106.5841 | 29.602583 | 600876 | Upper | Riverine zone | 2021/8/20 | Summer | 146.5 | 30.2 | 7.85 | 7.61 | 12.86 | 405 | 187 | 282 | 0.009 | 1.410 | 0.191 | 1.617 | 0.050 | 571381 | 178542439 | 312.48 |
| S3-3 | NA | Nanan | 106.5841 | 29.602583 | 600876 | Upper | Riverine zone | 2021/8/20 | Summer | 146.5 | 28.9 | 7.95 | 7.41 | 12.86 | 398 | 189 | 277 | 0.013 | 1.402 | 0.257 | 1.669 | 0.074 | 748688 | 234013993 | 312.57 |
| S4-1 | CS | Changshou | 107.0894 | 29.802133 | 526643 | Upper | Riverine zone | 2021/8/20 | Summer | 146.5 | 29.1 | 8.02 | 7.41 | 10.00 | 392 | 187 | 277 | 0.017 | 1.176 | 0.270 | 1.565 | 0.099 | 686666 | 214637353 | 312.58 |
| S4-2 | CS | Changshou | 107.0894 | 29.802133 | 526643 | Upper | Riverine zone | 2021/8/20 | Summer | 146.5 | 29.7 | 7.98 | 7.31 | 8.57 | 391 | 187 | 287 | 0.015 | 1.233 | 0.283 | 1.544 | 0.102 | 552314 | 172693570 | 312.67 |
| S4-3 | CS | Changshou | 107.0894 | 29.802133 | 526643 | Upper | Riverine zone | 2021/8/20 | Summer | 146.5 | 28.1 | 7.99 | 7.70 | 9.29 | 383 | 182 | 262 | 0.015 | 1.257 | 0.336 | 1.575 | 0.074 | 735163 | 229769110 | 312.54 |
| S5-1 | FL | Fuling | 107.5274 | 29.858788 | 450995 | Middle | Tranzition zone | 2021/8/21 | Summer | 146.5 | 27.9 | 7.93 | 7.70 | 24.29 | 377 | 175 | 262 | 0.014 | 1.135 | 0.164 | 1.690 | 0.178 | 695580 | 217675490 | 312.94 |
| S5-2 | FL | Fuling | 107.5274 | 29.858788 | 450995 | Middle | Tranzition zone | 2021/8/21 | Summer | 146.5 | 28.0 | 7.90 | 7.53 | 27.14 | 377 | 176 | 264 | 0.015 | 1.176 | 0.191 | 1.659 | 0.071 | 718257 | 224838714 | 313.03 |
| S5-3 | FL | Fuling | 107.5274 | 29.858788 | 450995 | Middle | Tranzition zone | 2021/8/21 | Summer | 146.5 | 28.7 | 7.97 | 7.57 | 31.43 | 376 | 179 | 266 | 0.012 | 1.402 | 0.191 | 1.544 | 0.041 | 656526 | 205371330 | 312.82 |
| S6-1 | FD | Fengdu | 107.7204 | 29.871405 | 431048 | Middle | Tranzition zone | 2021/8/21 | Summer | 146.5 | 29.9 | 7.91 | 7.28 | 18.57 | 375 | 182 | 257 | 0.012 | 1.087 | 0.434 | 1.617 | 0.059 | 548065 | 171425676 | 312.78 |
| S6-2 | FD | Fengdu | 107.7204 | 29.871405 | 431048 | Middle | Tranzition zone | 2021/8/21 | Summer | 146.5 | 31.1 | 7.92 | 7.33 | 14.29 | 383 | 180 | 266 | 0.013 | 1.014 | 0.434 | 1.565 | 0.065 | 698655 | 218479610 | 312.71 |
| S6-3 | FD | Fengdu | 107.7204 | 29.871405 | 431048 | Middle | Tranzition zone | 2021/8/21 | Summer | 146.5 | 29.6 | 7.85 | 7.35 | 10.00 | 388 | 183 | 272 | 0.014 | 1.051 | 0.638 | 1.617 | 0.059 | 678083 | 212082627 | 312.77 |
| S7-1 | ZX | Zhongxian | 107.9977 | 30.26792 | 372779 | Middle | Tranzition zone | 2021/8/21 | Summer | 146.5 | 32.3 | 7.83 | 6.65 | 27.14 | 377 | 177 | 262 | 0.043 | 1.095 | 0.612 | 1.709 | 0.071 | 623787 | 194055851 | 311.09 |
| S7-2 | ZX | Zhongxian | 107.9977 | 30.26792 | 372779 | Middle | Tranzition zone | 2021/8/21 | Summer | 146.5 | 32.8 | 7.86 | 6.98 | 22.86 | 370 | 168 | 257 | 0.025 | 0.966 | 0.401 | 1.709 | 0.053 | 703106 | 219931521 | 312.8 |
| S7-3 | ZX | Zhongxian | 107.9977 | 30.26792 | 372779 | Middle | Tranzition zone | 2021/8/21 | Summer | 146.5 | 31.8 | 7.83 | 6.92 | 22.86 | 371 | 168 | 253 | 0.031 | 1.184 | 0.454 | 1.709 | 0.062 | 628040 | 196486793 | 312.86 |
| S8-1 | WZ | Wanzhou | 108.3895 | 30.794857 | 283706 | Middle | Tranzition zone | 2021/8/21 | Summer | 146.5 | 27.4 | 7.74 | 7.74 | 14.29 | 380 | 179 | 266 | 0.009 | 1.475 | 0.217 | 1.804 | 0.068 | 748199 | 234165227 | 312.97 |
| S8-2 | WZ | Wanzhou | 108.3895 | 30.794857 | 283706 | Middle | Tranzition zone | 2021/8/21 | Summer | 146.5 | 26.8 | 7.50 | 7.50 | 17.14 | 378 | 181 | 261 | 0.014 | 1.499 | 0.270 | 1.752 | 0.050 | 660284 | 206628762 | 312.94 |
| S8-3 | WZ | Wanzhou | 108.3895 | 30.794857 | 283706 | Middle | Tranzition zone | 2021/8/21 | Summer | 146.5 | 27.2 | 7.53 | 7.53 | 12.86 | 379 | 178 | 264 | 0.018 | 1.499 | 0.362 | 1.898 | 0.062 | 651532 | 203961050 | 313.05 |
| S9-1 | YY | Yunyang | 108.7199 | 30.920005 | 241557 | Lower | Tranzition zone | 2021/8/22 | Summer | 146.5 | 27.4 | 8.01 | 7.78 | 12.86 | 378 | 178 | 263 | 0.017 | 1.499 | 0.270 | 1.607 | 0.059 | 721466 | 226024786 | 313.29 |
| S9-2 | YY | Yunyang | 108.7199 | 30.920005 | 241557 | Lower | Tranzition zone | 2021/8/22 | Summer | 146.5 | 27.7 | 7.95 | 7.63 | 12.86 | 370 | 177 | 263 | 0.016 | 1.491 | 0.257 | 1.721 | 0.059 | 521635 | 163432171 | 313.31 |
| S9-3 | YY | Yunyang | 108.7199 | 30.920005 | 241557 | Lower | Tranzition zone | 2021/8/22 | Summer | 146.5 | 28.9 | 7.97 | 7.39 | 14.29 | 370 | 178 | 261 | 0.020 | 1.467 | 0.322 | 1.809 | 0.077 | 547937 | 171808256 | 313.55 |
| S10-1 | FJ | Fengjie | 109.5051 | 31.03669 | 162083 | Lower | Lacustrine zone | 2021/8/22 | Summer | 146.5 | 29.0 | 7.93 | 7.01 | 17.14 | 370 | 178 | 255 | 0.028 | 1.435 | 0.336 | 1.742 | 0.105 | 693191 | 217367690 | 313.58 |
| S10-2 | FJ | Fengjie | 109.5051 | 31.03669 | 162083 | Lower | Lacustrine zone | 2021/8/22 | Summer | 146.5 | 30.1 | 7.94 | 6.90 | 14.29 | 367 | 174 | 257 | 0.025 | 1.427 | 0.336 | 1.981 | 0.056 | 500048 | 156627808 | 313.23 |
| S10-3 | FJ | Fengjie | 109.5051 | 31.03669 | 162083 | Lower | Lacustrine zone | 2021/8/22 | Summer | 146.5 | 30.1 | 7.72 | 6.80 | 15.71 | 367 | 171 | 256 | 0.022 | 1.427 | 0.336 | 1.867 | 0.059 | 695227 | 217674541 | 313.1 |
| S11-1 | WS | Wushan | 109.9171 | 31.059443 | 119292 | Lower | Lacustrine zone | 2021/8/22 | Summer | 146.5 | 27.1 | 7.84 | 7.38 | 18.57 | 368 | 173 | 256 | 0.013 | 1.443 | 0.336 | 1.791 | 0.065 | 732172 | 228742044 | 312.42 |
| S11-2 | WS | Wushan | 109.9171 | 31.059443 | 119292 | Lower | Lacustrine zone | 2021/8/22 | Summer | 146.5 | 27.7 | 7.97 | 7.23 | 20.00 | 365 | 174 | 258 | 0.014 | 1.459 | 0.296 | 1.769 | 0.068 | 578789 | 181033627 | 312.78 |
| S11-3 | WS | Wushan | 109.9171 | 31.059443 | 119292 | Lower | Lacustrine zone | 2021/8/22 | Summer | 146.5 | 28.2 | 7.98 | 7.15 | 17.14 | 367 | 177 | 254 | 0.017 | 1.459 | 0.309 | 1.785 | 0.074 | 655481 | 204887835.5 | 312.6 |
| S12-1 | BD | Badong | 110.3321 | 31.047456 | 75183 | Lower | Lacustrine zone | 2021/8/23 | Summer | 146.5 | 25.3 | 7.95 | 7.77 | 17.14 | 392 | 183 | 273 | 0.025 | 1.330 | 0.467 | 1.822 | 0.114 | 587158 | 183882277 | 313.17 |
| S12-2 | BD | Badong | 110.3321 | 31.047456 | 75183 | Lower | Lacustrine zone | 2021/8/23 | Summer | 146.5 | 25.6 | 7.90 | 7.66 | 12.86 | 391 | 185 | 271 | 0.023 | 1.354 | 0.362 | 1.738 | 0.151 | 697390 | 218986010 | 314.01 |
| S12-3 | BD | Badong | 110.3321 | 31.047456 | 75183 | Lower | Lacustrine zone | 2021/8/23 | Summer | 146.5 | 25.8 | 7.95 | 7.60 | 10.00 | 393 | 185 | 272 | 0.027 | 1.548 | 0.428 | 2.003 | 0.090 | 655254 | 205133282 | 313.06 |
| S13-1 | ZG | Zigui | 110.9735 | 30.845309 | 3685 | Lower | Lacustrine zone | 2021/8/23 | Summer | 146.5 | 26.0 | 7.75 | 7.19 | 12.86 | 364 | 177 | 256 | 0.030 | 1.427 | 0.441 | 1.898 | 0.191 | 644245 | 201598441 | 312.92 |
| S13-2 | ZG | Zigui | 110.9735 | 30.845309 | 3685 | Lower | Lacustrine zone | 2021/8/23 | Summer | 146.5 | 26.0 | 7.75 | 7.15 | 15.00 | 360 | 172 | 257 | 0.030 | 1.613 | 0.454 | 2.097 | 0.080 | 566510 | 177259055 | 312.9 |
| S13-3 | ZG | Zigui | 110.9735 | 30.845309 | 3685 | Lower | Lacustrine zone | 2021/8/23 | Summer | 146.5 | 25.9 | 7.93 | 7.31 | 17.14 | 365 | 180 | 251 | 0.031 | 1.394 | 0.520 | 1.945 | 0.108 | 721980 | 225937827 | 312.94 |
| S14-1 | YL | Yiling | 111.0823 | 30.856624 | -9035 | Lower | Lacustrine zone | 2021/8/23 | Summer | 146.5 | 26.1 | 7.95 | 7.43 | 24.29 | 368 | 173 | 256 | 0.031 | 1.435 | 0.507 | 1.973 | 0.240 | 538717 | 168552786 | 312.88 |
| S14-2 | YL | Yiling | 111.0823 | 30.856624 | -9035 | Lower | Lacustrine zone | 2021/8/23 | Summer | 146.5 | 26.1 | 7.65 | 7.29 | 22.86 | 366 | 172 | 254 | 0.030 | 1.265 | 0.401 | 1.696 | 0.062 | 604933 | 189568013 | 313.37 |
| S14-3 | YL | Yiling | 111.0823 | 30.856624 | -9035 | Lower | Lacustrine zone | 2021/8/23 | Summer | 146.5 | 26.3 | 7.93 | 7.22 | 18.57 | 379 | 175 | 258 | 0.032 | 1.362 | 0.428 | 1.822 | 0.071 | 705806 | 221047507 | 313.18 |
| W1-1 | JJ | Jiangjin | 106.2207 | 29.21483 | 687946 | Upper | Riverine zone | 2021/12/26 | Winter | 172 | 14.8 | 8.32 | 9.41 | 23.33 | 427 | 204 | 297 | 0.012 | 1.396 | 0.057 | 1.567 | 0.043 | 710287 | 222587945 | 313.38 |
| W1-2 | JJ | Jiangjin | 106.2207 | 29.21483 | 687946 | Upper | Riverine zone | 2021/12/26 | Winter | 172 | 14.4 | 8.26 | 9.31 | 23.33 | 421 | 202 | 299 | 0.012 | 1.383 | 0.037 | 1.588 | 0.046 | 502047 | 157331506 | 313.38 |
| W1-3 | JJ | Jiangjin | 106.2207 | 29.21483 | 687946 | Upper | Riverine zone | 2021/12/26 | Winter | 172 | 14.5 | 8.27 | 9.65 | 23.33 | 420 | 199 | 294 | 0.012 | 1.401 | 0.047 | 1.722 | 0.037 | 603030 | 189081032 | 313.55 |
| W2-1 | BN | Banan | 106.5426 | 29.473633 | 620510 | Upper | Riverine zone | 2021/12/26 | Winter | 172 | 14.5 | 8.33 | 9.51 | 15.00 | 421 | 203 | 296 | 0.017 | 1.579 | 0.107 | 1.804 | 0.031 | 696372 | 218501560 | 313.77 |
| W2-2 | BN | Banan | 106.5426 | 29.473633 | 620510 | Upper | Riverine zone | 2021/12/26 | Winter | 172 | 14.1 | 8.21 | 9.47 | 15.00 | 420 | 200 | 292 | 0.017 | 1.557 | 0.153 | 1.753 | 0.022 | 749246 | 234969386 | 313.61 |
| W2-3 | BN | Banan | 106.5426 | 29.473633 | 620510 | Upper | Riverine zone | 2021/12/26 | Winter | 172 | 14.1 | 8.25 | 9.48 | 15.00 | 421 | 199 | 294 | 0.017 | 1.570 | 0.072 | 1.814 | 0.028 | 580427 | 181921064 | 313.43 |
| W3-1 | NA | Nanan | 106.5841 | 29.602583 | 600876 | Upper | Riverine zone | 2021/12/26 | Winter | 172 | 13.8 | 8.29 | 9.49 | 11.67 | 417 | 201 | 291 | 0.016 | 1.610 | 0.098 | 1.845 | 0.034 | 694508 | 217455925 | 313.11 |
| W3-2 | NA | Nanan | 106.5841 | 29.602583 | 600876 | Upper | Riverine zone | 2021/12/26 | Winter | 172 | 13.7 | 8.15 | 9.42 | 11.67 | 415 | 197 | 287 | 0.017 | 1.583 | 0.080 | 1.794 | 0.040 | 688670 | 216086395 | 313.77 |
| W3-3 | NA | Nanan | 106.5841 | 29.602583 | 600876 | Upper | Riverine zone | 2021/12/26 | Winter | 172 | 14.0 | 8.23 | 9.25 | 11.67 | 417 | 195 | 293 | 0.016 | 1.561 | 0.083 | 1.742 | 0.022 | 569868 | 178561989 | 313.34 |
| W4-1 | CS | Changshou | 107.0894 | 29.802133 | 526643 | Upper | Riverine zone | 2021/12/26 | Winter | 172 | 13.6 | 8.25 | 9.53 | 15.00 | 425 | 200 | 300 | 0.015 | 1.771 | 0.118 | 1.742 | 0.018 | 549197 | 172600493 | 314.28 |
| W4-2 | CS | Changshou | 107.0894 | 29.802133 | 526643 | Upper | Riverine zone | 2021/12/26 | Winter | 172 | 13.8 | 8.25 | 9.58 | 15.00 | 422 | 204 | 297 | 0.015 | 1.539 | 0.108 | 1.742 | 0.020 | 514742 | 162013740 | 314.75 |
| W4-3 | CS | Changshou | 107.0894 | 29.802133 | 526643 | Upper | Riverine zone | 2021/12/26 | Winter | 172 | 14.3 | 8.25 | 9.47 | 15.00 | 425 | 199 | 291 | 0.015 | 1.561 | 0.058 | 1.732 | 0.022 | 648804 | 204180178 | 314.7 |
| W5-1 | FL | Fuling | 107.5274 | 29.858788 | 450995 | Middle | Tranzition zone | 2021/12/26 | Winter | 172 | 14.4 | 8.35 | 9.23 | 13.33 | 422 | 208 | 297 | 0.021 | 1.543 | 0.077 | 1.794 | 0.049 | 406088 | 127442806 | 313.83 |
| W5-2 | FL | Fuling | 107.5274 | 29.858788 | 450995 | Middle | Tranzition zone | 2021/12/26 | Winter | 172 | 14.1 | 8.20 | 9.13 | 13.33 | 418 | 196 | 295 | 0.019 | 1.530 | 0.053 | 1.742 | 0.043 | 450752 | 141545393 | 314.02 |
| W5-3 | FL | Fuling | 107.5274 | 29.858788 | 450995 | Middle | Tranzition zone | 2021/12/26 | Winter | 172 | 14.5 | 8.17 | 9.28 | 13.33 | 420 | 207 | 296 | 0.022 | 1.521 | 0.095 | 1.722 | 0.055 | 470945 | 147944226 | 314.14 |
| W6-1 | FD | Fengdu | 107.7204 | 29.871405 | 431048 | Middle | Tranzition zone | 2021/12/27 | Winter | 172 | 14.1 | 8.22 | 9.44 | 16.67 | 413 | 198 | 291 | 0.025 | 1.525 | 0.055 | 1.691 | 0.092 | 500069 | 156951812 | 313.86 |
| W6-2 | FD | Fengdu | 107.7204 | 29.871405 | 431048 | Middle | Tranzition zone | 2021/12/27 | Winter | 172 | 14.2 | 8.25 | 9.29 | 16.67 | 415 | 196 | 289 | 0.022 | 1.543 | 0.064 | 1.680 | 0.083 | 512015 | 160581405 | 313.63 |
| W6-3 | FD | Fengdu | 107.7204 | 29.871405 | 431048 | Middle | Tranzition zone | 2021/12/27 | Winter | 172 | 14.6 | 8.19 | 9.04 | 16.67 | 415 | 196 | 290 | 0.025 | 1.552 | 0.073 | 1.722 | 0.074 | 536849 | 168553593 | 313.97 |
| W7-1 | ZX | Zhongxian | 107.9977 | 30.26792 | 372779 | Middle | Tranzition zone | 2021/12/27 | Winter | 172 | 14.3 | 8.20 | 9.41 | 23.33 | 409 | 193 | 284 | 0.044 | 1.517 | 0.028 | 1.701 | 0.022 | 500991 | 157318514 | 314.01 |
| W7-2 | ZX | Zhongxian | 107.9977 | 30.26792 | 372779 | Middle | Tranzition zone | 2021/12/27 | Winter | 172 | 14.2 | 8.21 | 9.20 | 23.33 | 410 | 193 | 294 | 0.045 | 1.597 | 0.015 | 1.629 | 0.015 | 514797 | 161538484 | 313.79 |
| W7-3 | ZX | Zhongxian | 107.9977 | 30.26792 | 372779 | Middle | Tranzition zone | 2021/12/27 | Winter | 172 | 14.6 | 8.17 | 9.05 | 23.33 | 406 | 200 | 287 | 0.046 | 1.508 | 0.022 | 1.629 | 0.015 | 740974 | 232878147 | 314.29 |
| W8-1 | WZ | Wanzhou | 108.3895 | 30.794857 | 283706 | Middle | Tranzition zone | 2021/12/27 | Winter | 172 | 13.9 | 7.98 | 9.57 | 25.00 | 401 | 184 | 284 | 0.013 | 1.543 | 0.032 | 1.619 | 0.062 | 578005 | 181193980 | 313.48 |
| W8-2 | WZ | Wanzhou | 108.3895 | 30.794857 | 283706 | Middle | Tranzition zone | 2021/12/27 | Winter | 172 | 14.6 | 8.06 | 9.22 | 25.00 | 390 | 186 | 274 | 0.013 | 1.570 | 0.067 | 1.742 | 0.086 | 721801 | 226231486 | 313.43 |
| W8-3 | WZ | Wanzhou | 108.3895 | 30.794857 | 283706 | Middle | Tranzition zone | 2021/12/27 | Winter | 172 | 14.6 | 8.07 | 9.23 | 25.00 | 399 | 186 | 274 | 0.013 | 1.588 | 0.068 | 1.639 | 0.077 | 652857 | 204652076 | 313.47 |
| W9-1 | YY | Yunyang | 108.7199 | 30.920005 | 241557 | Lower | Tranzition zone | 2021/12/28 | Winter | 172 | 14.3 | 8.13 | 9.27 | 16.67 | 387 | 182 | 270 | 0.005 | 1.530 | 0.050 | 1.608 | 0.031 | 709886 | 222465763 | 313.38 |
| W9-2 | YY | Yunyang | 108.7199 | 30.920005 | 241557 | Lower | Tranzition zone | 2021/12/28 | Winter | 172 | 14.3 | 8.14 | 8.96 | 16.67 | 387 | 185 | 271 | 0.005 | 1.521 | 0.047 | 1.546 | 0.037 | 676402 | 211873873 | 313.24 |
| W9-3 | YY | Yunyang | 108.7199 | 30.920005 | 241557 | Lower | Tranzition zone | 2021/12/28 | Winter | 172 | 14.4 | 8.11 | 9.08 | 16.67 | 392 | 180 | 270 | 0.004 | 1.534 | 0.043 | 1.608 | 0.034 | 580502 | 181841303 | 313.25 |
| W10-1 | FJ | Fengjie | 109.5051 | 31.03669 | 162083 | Lower | Lacustrine zone | 2021/12/28 | Winter | 172 | 15.4 | 8.06 | 8.92 | 11.67 | 399 | 188 | 280 | 0.014 | 1.588 | 0.142 | 1.918 | 0.197 | 733467 | 229727206 | 313.21 |
| W10-2 | FJ | Fengjie | 109.5051 | 31.03669 | 162083 | Lower | Lacustrine zone | 2021/12/28 | Winter | 172 | 14.8 | 8.07 | 9.01 | 11.67 | 396 | 190 | 277 | 0.016 | 1.552 | 0.118 | 1.866 | 0.200 | 675156 | 211693535 | 313.55 |
| W10-3 | FJ | Fengjie | 109.5051 | 31.03669 | 162083 | Lower | Lacustrine zone | 2021/12/28 | Winter | 172 | 14.6 | 8.07 | 9.16 | 11.67 | 396 | 187 | 275 | 0.014 | 1.530 | 0.117 | 1.649 | 0.215 | 748540 | 234458041 | 313.22 |
| W11-1 | WS | Wushan | 109.9171 | 31.059443 | 119292 | Lower | Lacustrine zone | 2021/12/29 | Winter | 172 | 16.5 | 8.18 | 9.88 | 16.67 | 387 | 183 | 277 | 0.009 | 1.512 | 0.073 | 1.598 | 0.034 | 684161 | 214281005 | 313.2 |
| W11-2 | WS | Wushan | 109.9171 | 31.059443 | 119292 | Lower | Lacustrine zone | 2021/12/29 | Winter | 172 | 15.7 | 8.20 | 9.80 | 16.67 | 385 | 186 | 270 | 0.008 | 1.517 | 0.042 | 1.629 | 0.022 | 504964 | 158147115 | 313.18 |
| W11-3 | WS | Wushan | 109.9171 | 31.059443 | 119292 | Lower | Lacustrine zone | 2021/12/29 | Winter | 172 | 15.6 | 8.17 | 9.80 | 16.67 | 387 | 183 | 270 | 0.006 | 1.539 | 0.038 | 1.598 | 0.037 | 649457 | 203367517 | 313.13 |
| W12-1 | BD | Badong | 110.3321 | 31.047456 | 75183 | Lower | Lacustrine zone | 2021/12/28 | Winter | 172 | 15.1 | 8.20 | 9.23 | 13.33 | 396 | 186 | 274 | 0.007 | 1.521 | 0.042 | 1.619 | 0.012 | 618766 | 193905431 | 313.37 |
| W12-2 | BD | Badong | 110.3321 | 31.047456 | 75183 | Lower | Lacustrine zone | 2021/12/28 | Winter | 172 | 15.2 | 8.16 | 9.15 | 13.33 | 390 | 182 | 268 | 0.008 | 1.494 | 0.013 | 1.608 | 0.009 | 518105 | 162160549 | 312.99 |
| W12-3 | BD | Badong | 110.3321 | 31.047456 | 75183 | Lower | Lacustrine zone | 2021/12/28 | Winter | 172 | 15.0 | 8.15 | 9.19 | 13.33 | 389 | 183 | 276 | 0.009 | 1.517 | 0.012 | 1.629 | 0.011 | 552996 | 173385225 | 313.54 |
| W13-1 | ZG | Zigui | 110.9735 | 30.845309 | 3685 | Lower | Lacustrine zone | 2021/12/29 | Winter | 172 | 15.6 | 8.15 | 9.28 | 13.33 | 391 | 187 | 275 | 0.009 | 1.490 | 0.070 | 1.588 | 0.166 | 520014 | 162937863 | 313.33 |
| W13-2 | ZG | Zigui | 110.9735 | 30.845309 | 3685 | Lower | Lacustrine zone | 2021/12/29 | Winter | 172 | 14.9 | 8.16 | 9.28 | 13.33 | 393 | 186 | 274 | 0.008 | 1.503 | 0.043 | 1.619 | 0.142 | 550879 | 172605517 | 313.33 |
| W13-3 | ZG | Zigui | 110.9735 | 30.845309 | 3685 | Lower | Lacustrine zone | 2021/12/29 | Winter | 172 | 15.8 | 8.24 | 9.28 | 13.33 | 397 | 185 | 273 | 0.009 | 1.561 | 0.057 | 1.536 | 0.188 | 386622 | 121116206 | 313.27 |
| W14-1 | YL | Yiling | 111.0823 | 30.856624 | -9035 | Lower | Lacustrine zone | 2021/12/29 | Winter | 172 | 14.0 | 8.24 | 9.10 | 18.33 | 393 | 180 | 275 | 0.009 | 1.485 | 0.042 | 1.619 | 0.065 | 541435 | 169727217 | 313.48 |
| W14-2 | YL | Yiling | 111.0823 | 30.856624 | -9035 | Lower | Lacustrine zone | 2021/12/29 | Winter | 172 | 15.0 | 8.26 | 9.14 | 18.33 | 395 | 189 | 277 | 0.009 | 1.490 | 0.018 | 1.814 | 0.049 | 617936 | 193596511 | 313.3 |
| W14-3 | YL | Yiling | 111.0823 | 30.856624 | -9035 | Lower | Lacustrine zone | 2021/12/29 | Winter | 172 | 15.3 | 8.21 | 9.00 | 18.33 | 403 | 184 | 276 | 0.009 | 1.476 | 0.032 | 1.742 | 0.046 | 591188 | 185203003 | 313.27 |

**Notice:** T, water temperature; DO, dissolved oxygen; COD, chemical oxygen demand; EC, electrical conductivity; TDS, total dissolved solids; $NO_2$-N, nitrite nitrogen; $NO_3$-N, nitrate nitrogen; $NH_4$-N, ammonium nitrogen; TN, total nitrogen; TP, total phosphorus.

**Table S2. Comparison of environmental variables between summer and winter**

| Environmental variables | Summer (n=42) | | Winter (n=42) | | *p* |
|---|---|---|---|---|---|
| | Mean | SE | Mean | SE | |
| T (℃) | **28.26** | **0.28** | **14.60** | **0.10** | **0.000** |
| pH | **7.89** | **0.02** | **8.19** | **0.01** | **0.000** |
| DO (mg/L) | **7.36** | **0.04** | **9.31** | **0.04** | **0.000** |
| COD (mg/L) | 15.92 | 0.86 | 16.67 | 0.66 | 0.228 |
| EC (μS/cm) | **379.31** | **1.88** | **405.52** | **2.12** | **0.000** |
| Salinity (mg/L) | **179.62** | **0.97** | **192.19** | **1.26** | **0.000** |
| TDS (mg/L) | **264.45** | **1.40** | **283.67** | **1.59** | **0.000** |
| $NO_2$-N (mg/L) | **0.020** | **0.00** | **0.015** | **0.00** | **0.003** |
| $NO_3$-N (mg/L) | **1.345** | **0.03** | **1.533** | **0.01** | **0.000** |
| $NH_4$-N (mg/L) | **0.323** | **0.02** | **0.063** | **0.01** | **0.000** |
| TN (mg/L) | 1.754 | 0.02 | 1.689 | 0.01 | 0.114 |
| TP (mg/L) | **0.084** | **0.01** | **0.060** | **0.01** | **0.000** |

**Table S3. Annotation results of representative sequences under different similarity thresholds at the species level**

| Similarity thresholds | 97% | 95% | 90% | 80% |
|---|---|---|---|---|
| Classified ASVs | 3394 | 1985 | 4346 | 3768 |
| Remaining unclassified ASVs | 14273 | 12288 | 7942 | 4174 |
| Cumulative classified ASVs | 3394 | 5379 | 9725 | 13493 |

**Table S4. The ASV richness, and Shannon index of meio- and microeukaryotic, zooplankton, and zoobenthos communities in the 84 samples**

| Taxa | Meio- and microeukaryotes (All ASVs) | | Zooplankton | | Zoobenthos | |
|---|---|---|---|---|---|---|
| SampleID | ASV richness | Shannon | ASV richness | Shannon | ASV richness | Shannon |
| S1-1 | 4570 | 8.7 | 1466 | 7.0 | 487 | 5.2 |
| S1-2 | 5144 | 9.1 | 1645 | 7.5 | 592 | 5.8 |
| S1-3 | 4612 | 9.0 | 1487 | 7.3 | 480 | 6.3 |
| S2-1 | 4884 | 8.9 | 1574 | 7.4 | 578 | 4.8 |
| S2-2 | 6912 | 9.5 | 1967 | 7.7 | 743 | 5.3 |
| S2-3 | 5902 | 9.2 | 1748 | 7.5 | 613 | 5.2 |
| S3-1 | 4430 | 8.5 | 1520 | 7.1 | 492 | 4.4 |
| S3-2 | 5286 | 8.7 | 1566 | 7.4 | 596 | 4.2 |
| S3-3 | 4387 | 8.6 | 1442 | 7.4 | 486 | 4.6 |
| S4-1 | 4556 | 8.8 | 1539 | 7.5 | 513 | 4.0 |
| S4-2 | 3900 | 8.2 | 1333 | 7.0 | 481 | 3.7 |
| S4-3 | 3945 | 8.7 | 1366 | 7.4 | 450 | 4.5 |
| S5-1 | 2978 | 7.7 | 1028 | 6.6 | 337 | 4.9 |
| S5-2 | 4843 | 8.4 | 1403 | 7.0 | 532 | 5.3 |
| S5-3 | 4596 | 8.8 | 1417 | 7.3 | 514 | 5.2 |
| S6-1 | 3559 | 8.2 | 1315 | 7.0 | 443 | 4.5 |
| S6-2 | 4410 | 8.5 | 1458 | 7.1 | 550 | 4.6 |
| S6-3 | 3885 | 8.2 | 1365 | 6.7 | 498 | 4.2 |
| S7-1 | 2803 | 7.5 | 971 | 5.5 | 261 | 5.6 |
| S7-2 | 1607 | 6.0 | 541 | 5.7 | 196 | 5.1 |
| S7-3 | 2320 | 7.2 | 709 | 6.2 | 209 | 5.5 |
| S8-1 | 3418 | 8.2 | 1084 | 6.4 | 353 | 5.1 |
| S8-2 | 2870 | 7.7 | 883 | 6.5 | 280 | 4.3 |
| S8-3 | 3641 | 8.2 | 1094 | 6.1 | 359 | 5.3 |
| S9-1 | 2653 | 8.0 | 737 | 5.9 | 210 | 4.9 |
| S9-2 | 3928 | 8.1 | 959 | 6.4 | 282 | 5.3 |
| S9-3 | 3613 | 8.8 | 942 | 6.9 | 275 | 5.5 |
| S10-1 | 3338 | 8.1 | 674 | 6.5 | 206 | 4.8 |
| S10-2 | 3417 | 7.8 | 608 | 5.8 | 209 | 5.2 |
| S10-3 | 3396 | 8.2 | 742 | 6.0 | 227 | 5.2 |
| S11-1 | 2931 | 6.2 | 802 | 4.0 | 235 | 5.5 |
| S11-2 | 3045 | 7.8 | 767 | 5.6 | 234 | 5.2 |
| S11-3 | 3902 | 7.7 | 1027 | 5.3 | 316 | 5.9 |
| S12-1 | 2982 | 7.7 | 804 | 5.4 | 247 | 5.0 |
| S12-2 | 3592 | 8.4 | 808 | 5.9 | 270 | 5.4 |
| S12-3 | 3772 | 8.1 | 885 | 5.7 | 275 | 5.6 |
| S13-1 | 3027 | 6.7 | 454 | 6.1 | 148 | 5.0 |
| S13-2 | 1860 | 6.0 | 277 | 4.9 | 103 | 4.6 |
| S13-3 | 2497 | 6.2 | 361 | 5.9 | 104 | 4.3 |
| S14-1 | 1741 | 5.9 | 296 | 4.1 | 134 | 2.6 |
| S14-2 | 2067 | 6.0 | 354 | 3.1 | 92 | 4.5 |
| S14-3 | 2014 | 6.2 | 364 | 5.3 | 94 | 3.9 |
| W1-1 | 5383 | 8.2 | 1016 | 5.2 | 512 | 4.0 |
| W1-2 | 5403 | 8.1 | 964 | 4.7 | 456 | 4.0 |
| W1-3 | 5391 | 8.2 | 1012 | 5.0 | 459 | 3.8 |
| W2-1 | 5951 | 8.2 | 970 | 5.0 | 474 | 3.9 |
| W2-2 | 6201 | 8.4 | 1001 | 5.2 | 514 | 4.0 |
| W2-3 | 6308 | 8.5 | 1107 | 5.4 | 529 | 4.0 |
| W3-1 | 5857 | 8.4 | 1005 | 5.6 | 554 | 4.9 |
| W3-2 | 6145 | 8.4 | 1036 | 5.2 | 533 | 4.1 |
| W3-3 | 6271 | 8.5 | 1150 | 5.7 | 589 | 4.1 |

| | | | | | | |
|---|---|---|---|---|---|---|
| W4-1 | 6125 | 8.4 | 816 | 4.9 | 432 | 5.2 |
| W4-2 | 6032 | 8.3 | 753 | 4.7 | 435 | 5.4 |
| W4-3 | 5944 | 8.3 | 782 | 4.8 | 394 | 5.2 |
| W5-1 | 5343 | 7.9 | 717 | 4.1 | 425 | 6.4 |
| W5-2 | 5678 | 8.1 | 774 | 4.2 | 461 | 6.4 |
| W5-3 | 5433 | 7.9 | 713 | 3.9 | 436 | 5.5 |
| W6-1 | 4430 | 7.6 | 498 | 3.5 | 295 | 5.8 |
| W6-2 | 5023 | 7.4 | 572 | 3.4 | 309 | 4.8 |
| W6-3 | 4624 | 7.5 | 516 | 3.3 | 304 | 6.0 |
| W7-1 | 4867 | 7.6 | 494 | 3.5 | 233 | 5.7 |
| W7-2 | 4456 | 6.6 | 533 | 3.1 | 197 | 5.6 |
| W7-3 | 5013 | 8.0 | 445 | 3.3 | 275 | 5.7 |
| W8-1 | 4330 | 7.6 | 372 | 2.9 | 197 | 5.3 |
| W8-2 | 4627 | 7.6 | 399 | 2.7 | 242 | 5.7 |
| W8-3 | 4549 | 7.7 | 367 | 2.9 | 227 | 5.8 |
| W9-1 | 4489 | 7.8 | 439 | 3.1 | 257 | 4.5 |
| W9-2 | 4379 | 7.7 | 419 | 3.0 | 205 | 5.3 |
| W9-3 | 4402 | 7.4 | 376 | 2.8 | 220 | 5.6 |
| W10-1 | 4268 | 7.0 | 581 | 4.0 | 215 | 5.6 |
| W10-2 | 4128 | 7.3 | 401 | 4.7 | 238 | 5.5 |
| W10-3 | 4856 | 7.9 | 489 | 4.8 | 239 | 5.6 |
| W11-1 | 4079 | 7.4 | 361 | 4.6 | 178 | 5.2 |
| W11-2 | 3414 | 7.2 | 430 | 4.2 | 159 | 4.2 |
| W11-3 | 4148 | 7.5 | 404 | 4.9 | 212 | 5.0 |
| W12-1 | 3374 | 7.1 | 351 | 4.6 | 139 | 3.9 |
| W12-2 | 4303 | 7.2 | 445 | 5.2 | 218 | 4.6 |
| W12-3 | 3547 | 6.6 | 338 | 5.0 | 177 | 5.2 |
| W13-1 | 3973 | 7.7 | 362 | 4.5 | 179 | 5.2 |
| W13-2 | 3952 | 7.6 | 340 | 4.2 | 141 | 4.9 |
| W13-3 | 3793 | 7.6 | 332 | 4.5 | 166 | 4.2 |
| W14-1 | 3943 | 7.7 | 377 | 4.3 | 152 | 5.1 |
| W14-2 | 3879 | 7.5 | 362 | 4.3 | 167 | 5.2 |
| W14-3 | 4126 | 7.4 | 402 | 4.2 | 202 | 5.2 |

**Table S5. PCNM vector based on the longitude and latitude coordinates of 84 sampling sites for two seasons**

| SampleID | PCNM1 | PCNM2 | PCNM3 | PCNM4 | PCNM5 | PCNM6 | PCNM7 | PCNM8 | PCNM9 | PCNM10 |
|---|---|---|---|---|---|---|---|---|---|---|
| S1-1 | 0.0714 | -0.1530 | 0.0530 | 0.0696 | 0.1594 | -0.1939 | 0.1637 | 0.0041 | 0.0133 | -0.0172 |
| S1-2 | 0.0714 | -0.1530 | 0.0530 | 0.0696 | 0.1594 | -0.1939 | 0.1637 | 0.0041 | 0.0133 | -0.0172 |
| S1-3 | 0.0714 | -0.1530 | 0.0530 | 0.0696 | 0.1594 | -0.1939 | 0.1637 | 0.0041 | 0.0133 | -0.0172 |
| S2-1 | 0.1139 | -0.1617 | 0.0289 | 0.0221 | 0.0050 | 0.0486 | -0.0575 | 0.0215 | -0.2814 | 0.0850 |
| S2-2 | 0.1139 | -0.1617 | 0.0289 | 0.0221 | 0.0050 | 0.0486 | -0.0575 | 0.0215 | -0.2814 | 0.0850 |
| S2-3 | 0.1139 | -0.1617 | 0.0289 | 0.0221 | 0.0050 | 0.0486 | -0.0575 | 0.0215 | -0.2814 | 0.0850 |
| S3-1 | 0.1142 | -0.1612 | 0.0282 | 0.0209 | 0.0008 | 0.0564 | -0.1023 | -0.0254 | 0.2714 | -0.0679 |
| S3-2 | 0.1142 | -0.1612 | 0.0282 | 0.0209 | 0.0008 | 0.0564 | -0.1023 | -0.0254 | 0.2714 | -0.0679 |
| S3-3 | 0.1142 | -0.1612 | 0.0282 | 0.0209 | 0.0008 | 0.0564 | -0.1023 | -0.0254 | 0.2714 | -0.0679 |
| S4-1 | 0.1511 | -0.0227 | -0.0654 | -0.0904 | -0.1725 | 0.1465 | -0.0049 | 0.0005 | -0.0034 | 0.0002 |
| S4-2 | 0.1511 | -0.0227 | -0.0654 | -0.0904 | -0.1725 | 0.1465 | -0.0049 | 0.0005 | -0.0034 | 0.0002 |
| S4-3 | 0.1511 | -0.0227 | -0.0654 | -0.0904 | -0.1725 | 0.1465 | -0.0049 | 0.0005 | -0.0034 | 0.0002 |
| S5-1 | 0.1041 | 0.1364 | -0.0962 | -0.0688 | -0.0177 | -0.0661 | 0.1050 | -0.2786 | -0.0048 | 0.0374 |
| S5-2 | 0.1041 | 0.1364 | -0.0962 | -0.0688 | -0.0177 | -0.0661 | 0.1050 | -0.2786 | -0.0048 | 0.0374 |
| S5-3 | 0.1041 | 0.1364 | -0.0962 | -0.0688 | -0.0177 | -0.0661 | 0.1050 | -0.2786 | -0.0048 | 0.0374 |
| S6-1 | 0.1035 | 0.1375 | -0.0957 | -0.0670 | -0.0117 | -0.0718 | 0.0575 | 0.2890 | 0.0143 | -0.0540 |
| S6-2 | 0.1035 | 0.1375 | -0.0957 | -0.0670 | -0.0117 | -0.0718 | 0.0575 | 0.2890 | 0.0143 | -0.0540 |
| S6-3 | 0.1035 | 0.1375 | -0.0957 | -0.0670 | -0.0117 | -0.0718 | 0.0575 | 0.2890 | 0.0143 | -0.0540 |
| S7-1 | 0.0551 | 0.1769 | -0.0210 | 0.0868 | 0.1790 | -0.0215 | -0.1595 | -0.0107 | -0.0067 | 0.0175 |
| S7-2 | 0.0551 | 0.1769 | -0.0210 | 0.0868 | 0.1790 | -0.0215 | -0.1595 | -0.0107 | -0.0067 | 0.0175 |
| S7-3 | 0.0551 | 0.1769 | -0.0210 | 0.0868 | 0.1790 | -0.0215 | -0.1595 | -0.0107 | -0.0067 | 0.0175 |

| | | | | | | | | | | |
|---|---|---|---|---|---|---|---|---|---|---|
| S8-1 | -0.0195 | 0.1092 | 0.1305 | 0.1971 | 0.0375 | 0.1760 | -0.0064 | -0.0035 | -0.0031 | -0.0003 |
| S8-2 | -0.0195 | 0.1092 | 0.1305 | 0.1971 | 0.0375 | 0.1760 | -0.0064 | -0.0035 | -0.0031 | -0.0003 |
| S8-3 | -0.0195 | 0.1092 | 0.1305 | 0.1971 | 0.0375 | 0.1760 | -0.0064 | -0.0035 | -0.0031 | -0.0003 |
| S9-1 | -0.0574 | 0.0606 | 0.2005 | 0.0658 | -0.1769 | -0.0243 | 0.1587 | 0.0144 | 0.0092 | -0.0160 |
| S9-2 | -0.0574 | 0.0606 | 0.2005 | 0.0658 | -0.1769 | -0.0243 | 0.1587 | 0.0144 | 0.0092 | -0.0160 |
| S9-3 | -0.0574 | 0.0606 | 0.2005 | 0.0658 | -0.1769 | -0.0243 | 0.1587 | 0.0144 | 0.0092 | -0.0160 |
| S10-1 | -0.0852 | 0.0205 | 0.1701 | -0.1662 | -0.0645 | -0.1481 | -0.1539 | -0.0110 | -0.0069 | 0.0179 |
| S10-2 | -0.0852 | 0.0205 | 0.1701 | -0.1662 | -0.0645 | -0.1481 | -0.1539 | -0.0110 | -0.0069 | 0.0179 |
| S10-3 | -0.0852 | 0.0205 | 0.1701 | -0.1662 | -0.0645 | -0.1481 | -0.1539 | -0.0110 | -0.0069 | 0.0179 |
| S11-1 | -0.1166 | -0.0128 | 0.0533 | -0.2183 | 0.1547 | 0.1085 | -0.0040 | -0.0019 | -0.0021 | -0.0024 |
| S11-2 | -0.1166 | -0.0128 | 0.0533 | -0.2183 | 0.1547 | 0.1085 | -0.0040 | -0.0019 | -0.0021 | -0.0024 |
| S11-3 | -0.1166 | -0.0128 | 0.0533 | -0.2183 | 0.1547 | 0.1085 | -0.0040 | -0.0019 | -0.0021 | -0.0024 |
| S12-1 | -0.1657 | -0.0455 | -0.1130 | -0.0330 | 0.0698 | 0.1301 | 0.1571 | 0.0136 | 0.0087 | -0.0149 |
| S12-2 | -0.1657 | -0.0455 | -0.1130 | -0.0330 | 0.0698 | 0.1301 | 0.1571 | 0.0136 | 0.0087 | -0.0149 |
| S12-3 | -0.1657 | -0.0455 | -0.1130 | -0.0330 | 0.0698 | 0.1301 | 0.1571 | 0.0136 | 0.0087 | -0.0149 |
| S13-1 | -0.1348 | -0.0422 | -0.1369 | 0.0905 | -0.0810 | -0.0686 | -0.0558 | 0.0368 | 0.0765 | 0.2803 |
| S13-2 | -0.1348 | -0.0422 | -0.1369 | 0.0905 | -0.0810 | -0.0686 | -0.0558 | 0.0368 | 0.0765 | 0.2803 |
| S13-3 | -0.1348 | -0.0422 | -0.1369 | 0.0905 | -0.0810 | -0.0686 | -0.0558 | 0.0368 | 0.0765 | 0.2803 |
| S14-1 | -0.1341 | -0.0420 | -0.1363 | 0.0908 | -0.0819 | -0.0717 | -0.0978 | -0.0491 | -0.0851 | -0.2656 |
| S14-2 | -0.1341 | -0.0420 | -0.1363 | 0.0908 | -0.0819 | -0.0717 | -0.0978 | -0.0491 | -0.0851 | -0.2656 |
| S14-3 | -0.1341 | -0.0420 | -0.1363 | 0.0908 | -0.0819 | -0.0717 | -0.0978 | -0.0491 | -0.0851 | -0.2656 |
| W1-1 | 0.0714 | -0.1530 | 0.0530 | 0.0696 | 0.1594 | -0.1939 | 0.1637 | 0.0041 | 0.0133 | -0.0172 |
| W1-2 | 0.0714 | -0.1530 | 0.0530 | 0.0696 | 0.1594 | -0.1939 | 0.1637 | 0.0041 | 0.0133 | -0.0172 |

| | | | | | | | | | | |
|---|---|---|---|---|---|---|---|---|---|---|
| W1-3 | 0.0714 | -0.1530 | 0.0530 | 0.0696 | 0.1594 | -0.1939 | 0.1637 | 0.0041 | 0.0133 | -0.0172 |
| W2-1 | 0.1139 | -0.1617 | 0.0289 | 0.0221 | 0.0050 | 0.0486 | -0.0575 | 0.0215 | -0.2814 | 0.0850 |
| W2-2 | 0.1139 | -0.1617 | 0.0289 | 0.0221 | 0.0050 | 0.0486 | -0.0575 | 0.0215 | -0.2814 | 0.0850 |
| W2-3 | 0.1139 | -0.1617 | 0.0289 | 0.0221 | 0.0050 | 0.0486 | -0.0575 | 0.0215 | -0.2814 | 0.0850 |
| W3-1 | 0.1142 | -0.1612 | 0.0282 | 0.0209 | 0.0008 | 0.0564 | -0.1023 | -0.0254 | 0.2714 | -0.0679 |
| W3-2 | 0.1142 | -0.1612 | 0.0282 | 0.0209 | 0.0008 | 0.0564 | -0.1023 | -0.0254 | 0.2714 | -0.0679 |
| W3-3 | 0.1142 | -0.1612 | 0.0282 | 0.0209 | 0.0008 | 0.0564 | -0.1023 | -0.0254 | 0.2714 | -0.0679 |
| W4-1 | 0.1511 | -0.0227 | -0.0654 | -0.0904 | -0.1725 | 0.1465 | -0.0049 | 0.0005 | -0.0034 | 0.0002 |
| W4-2 | 0.1511 | -0.0227 | -0.0654 | -0.0904 | -0.1725 | 0.1465 | -0.0049 | 0.0005 | -0.0034 | 0.0002 |
| W4-3 | 0.1511 | -0.0227 | -0.0654 | -0.0904 | -0.1725 | 0.1465 | -0.0049 | 0.0005 | -0.0034 | 0.0002 |
| W5-1 | 0.1041 | 0.1364 | -0.0962 | -0.0688 | -0.0177 | -0.0661 | 0.1050 | -0.2786 | -0.0048 | 0.0374 |
| W5-2 | 0.1041 | 0.1364 | -0.0962 | -0.0688 | -0.0177 | -0.0661 | 0.1050 | -0.2786 | -0.0048 | 0.0374 |
| W5-3 | 0.1041 | 0.1364 | -0.0962 | -0.0688 | -0.0177 | -0.0661 | 0.1050 | -0.2786 | -0.0048 | 0.0374 |
| W6-1 | 0.1035 | 0.1375 | -0.0957 | -0.0670 | -0.0117 | -0.0718 | 0.0575 | 0.2890 | 0.0143 | -0.0540 |
| W6-2 | 0.1035 | 0.1375 | -0.0957 | -0.0670 | -0.0117 | -0.0718 | 0.0575 | 0.2890 | 0.0143 | -0.0540 |
| W6-3 | 0.1035 | 0.1375 | -0.0957 | -0.0670 | -0.0117 | -0.0718 | 0.0575 | 0.2890 | 0.0143 | -0.0540 |
| W7-1 | 0.0551 | 0.1769 | -0.0210 | 0.0868 | 0.1790 | -0.0215 | -0.1595 | -0.0107 | -0.0067 | 0.0175 |
| W7-2 | 0.0551 | 0.1769 | -0.0210 | 0.0868 | 0.1790 | -0.0215 | -0.1595 | -0.0107 | -0.0067 | 0.0175 |
| W7-3 | 0.0551 | 0.1769 | -0.0210 | 0.0868 | 0.1790 | -0.0215 | -0.1595 | -0.0107 | -0.0067 | 0.0175 |
| W8-1 | -0.0195 | 0.1092 | 0.1305 | 0.1971 | 0.0375 | 0.1760 | -0.0064 | -0.0035 | -0.0031 | -0.0003 |
| W8-2 | -0.0195 | 0.1092 | 0.1305 | 0.1971 | 0.0375 | 0.1760 | -0.0064 | -0.0035 | -0.0031 | -0.0003 |
| W8-3 | -0.0195 | 0.1092 | 0.1305 | 0.1971 | 0.0375 | 0.1760 | -0.0064 | -0.0035 | -0.0031 | -0.0003 |
| W9-1 | -0.0574 | 0.0606 | 0.2005 | 0.0658 | -0.1769 | -0.0243 | 0.1587 | 0.0144 | 0.0092 | -0.0160 |

| | | | | | | | | | | |
|---|---|---|---|---|---|---|---|---|---|---|
| W9-2 | -0.0574 | 0.0606 | 0.2005 | 0.0658 | -0.1769 | -0.0243 | 0.1587 | 0.0144 | 0.0092 | -0.0160 |
| W9-3 | -0.0574 | 0.0606 | 0.2005 | 0.0658 | -0.1769 | -0.0243 | 0.1587 | 0.0144 | 0.0092 | -0.0160 |
| W10-1 | -0.0852 | 0.0205 | 0.1701 | -0.1662 | -0.0645 | -0.1481 | -0.1539 | -0.0110 | -0.0069 | 0.0179 |
| W10-2 | -0.0852 | 0.0205 | 0.1701 | -0.1662 | -0.0645 | -0.1481 | -0.1539 | -0.0110 | -0.0069 | 0.0179 |
| W10-3 | -0.0852 | 0.0205 | 0.1701 | -0.1662 | -0.0645 | -0.1481 | -0.1539 | -0.0110 | -0.0069 | 0.0179 |
| W11-1 | -0.1166 | -0.0128 | 0.0533 | -0.2183 | 0.1547 | 0.1085 | -0.0040 | -0.0019 | -0.0021 | -0.0024 |
| W11-2 | -0.1166 | -0.0128 | 0.0533 | -0.2183 | 0.1547 | 0.1085 | -0.0040 | -0.0019 | -0.0021 | -0.0024 |
| W11-3 | -0.1166 | -0.0128 | 0.0533 | -0.2183 | 0.1547 | 0.1085 | -0.0040 | -0.0019 | -0.0021 | -0.0024 |
| W12-1 | -0.1657 | -0.0455 | -0.1130 | -0.0330 | 0.0698 | 0.1301 | 0.1571 | 0.0136 | 0.0087 | -0.0149 |
| W12-2 | -0.1657 | -0.0455 | -0.1130 | -0.0330 | 0.0698 | 0.1301 | 0.1571 | 0.0136 | 0.0087 | -0.0149 |
| W12-3 | -0.1657 | -0.0455 | -0.1130 | -0.0330 | 0.0698 | 0.1301 | 0.1571 | 0.0136 | 0.0087 | -0.0149 |
| W13-1 | -0.1348 | -0.0422 | -0.1369 | 0.0905 | -0.0810 | -0.0686 | -0.0558 | 0.0368 | 0.0765 | 0.2803 |
| W13-2 | -0.1348 | -0.0422 | -0.1369 | 0.0905 | -0.0810 | -0.0686 | -0.0558 | 0.0368 | 0.0765 | 0.2803 |
| W13-3 | -0.1348 | -0.0422 | -0.1369 | 0.0905 | -0.0810 | -0.0686 | -0.0558 | 0.0368 | 0.0765 | 0.2803 |
| W14-1 | -0.1341 | -0.0420 | -0.1363 | 0.0908 | -0.0819 | -0.0717 | -0.0978 | -0.0491 | -0.0851 | -0.2656 |
| W14-2 | -0.1341 | -0.0420 | -0.1363 | 0.0908 | -0.0819 | -0.0717 | -0.0978 | -0.0491 | -0.0851 | -0.2656 |
| W14-3 | -0.1341 | -0.0420 | -0.1363 | 0.0908 | -0.0819 | -0.0717 | -0.0978 | -0.0491 | -0.0851 | -0.2656 |

Notice: PCNM, Principal Coordinates of Neighborhood Matrices.

**Table S6. PCNM vector based on the longitude and latitude coordinates of 42 sampling sites for a single season**

| SampleID | PCNM1 | PCNM2 | PCNM3 | PCNM4 | PCNM5 | PCNM6 | PCNM7 | PCNM8 | PCNM9 | PCNM10 |
|---|---|---|---|---|---|---|---|---|---|---|
| 1-1 | 0.1010 | -0.2164 | 0.0749 | 0.0984 | 0.2255 | 0.2743 | 0.2315 | 0.0059 | 0.0188 | -0.0243 |
| 1-2 | 0.1010 | -0.2164 | 0.0749 | 0.0984 | 0.2255 | 0.2743 | 0.2315 | 0.0059 | 0.0188 | -0.0243 |
| 1-3 | 0.1010 | -0.2164 | 0.0749 | 0.0984 | 0.2255 | 0.2743 | 0.2315 | 0.0059 | 0.0188 | -0.0243 |
| 2-1 | 0.1611 | -0.2287 | 0.0408 | 0.0312 | 0.0070 | -0.0687 | -0.0814 | 0.0304 | -0.3980 | 0.1202 |
| 2-2 | 0.1611 | -0.2287 | 0.0408 | 0.0312 | 0.0070 | -0.0687 | -0.0814 | 0.0304 | -0.3980 | 0.1202 |
| 2-3 | 0.1611 | -0.2287 | 0.0408 | 0.0312 | 0.0070 | -0.0687 | -0.0814 | 0.0304 | -0.3980 | 0.1202 |
| 3-1 | 0.1616 | -0.2279 | 0.0399 | 0.0295 | 0.0011 | -0.0797 | -0.1447 | -0.0359 | 0.3838 | -0.0961 |
| 3-2 | 0.1616 | -0.2279 | 0.0399 | 0.0295 | 0.0011 | -0.0797 | -0.1447 | -0.0359 | 0.3838 | -0.0961 |
| 3-3 | 0.1616 | -0.2279 | 0.0399 | 0.0295 | 0.0011 | -0.0797 | -0.1447 | -0.0359 | 0.3838 | -0.0961 |
| 4-1 | 0.2136 | -0.0321 | -0.0925 | -0.1279 | -0.2440 | -0.2072 | -0.0069 | 0.0008 | -0.0048 | 0.0003 |
| 4-2 | 0.2136 | -0.0321 | -0.0925 | -0.1279 | -0.2440 | -0.2072 | -0.0069 | 0.0008 | -0.0048 | 0.0003 |
| 4-3 | 0.2136 | -0.0321 | -0.0925 | -0.1279 | -0.2440 | -0.2072 | -0.0069 | 0.0008 | -0.0048 | 0.0003 |
| 5-1 | 0.1473 | 0.1930 | -0.1360 | -0.0972 | -0.0250 | 0.0935 | 0.1485 | -0.3939 | -0.0068 | 0.0529 |
| 5-2 | 0.1473 | 0.1930 | -0.1360 | -0.0972 | -0.0250 | 0.0935 | 0.1485 | -0.3939 | -0.0068 | 0.0529 |
| 5-3 | 0.1473 | 0.1930 | -0.1360 | -0.0972 | -0.0250 | 0.0935 | 0.1485 | -0.3939 | -0.0068 | 0.0529 |
| 6-1 | 0.1463 | 0.1945 | -0.1354 | -0.0947 | -0.0166 | 0.1016 | 0.0813 | 0.4088 | 0.0202 | -0.0763 |
| 6-2 | 0.1463 | 0.1945 | -0.1354 | -0.0947 | -0.0166 | 0.1016 | 0.0813 | 0.4088 | 0.0202 | -0.0763 |
| 6-3 | 0.1463 | 0.1945 | -0.1354 | -0.0947 | -0.0166 | 0.1016 | 0.0813 | 0.4088 | 0.0202 | -0.0763 |
| 7-1 | 0.0779 | 0.2502 | -0.0298 | 0.1228 | 0.2532 | 0.0305 | -0.2255 | -0.0151 | -0.0095 | 0.0247 |
| 7-2 | 0.0779 | 0.2502 | -0.0298 | 0.1228 | 0.2532 | 0.0305 | -0.2255 | -0.0151 | -0.0095 | 0.0247 |
| 7-3 | 0.0779 | 0.2502 | -0.0298 | 0.1228 | 0.2532 | 0.0305 | -0.2255 | -0.0151 | -0.0095 | 0.0247 |
| 8-1 | -0.0275 | 0.1544 | 0.1846 | 0.2788 | 0.0531 | -0.2489 | -0.0091 | -0.0049 | -0.0043 | -0.0004 |

| | | | | | | | | | | |
|---|---|---|---|---|---|---|---|---|---|---|
| 8-2 | -0.0275 | 0.1544 | 0.1846 | 0.2788 | 0.0531 | -0.2489 | -0.0091 | -0.0049 | -0.0043 | -0.0004 |
| 8-3 | -0.0275 | 0.1544 | 0.1846 | 0.2788 | 0.0531 | -0.2489 | -0.0091 | -0.0049 | -0.0043 | -0.0004 |
| 9-1 | -0.0812 | 0.0856 | 0.2835 | 0.0931 | -0.2502 | 0.0343 | 0.2244 | 0.0204 | 0.0131 | -0.0226 |
| 9-2 | -0.0812 | 0.0856 | 0.2835 | 0.0931 | -0.2502 | 0.0343 | 0.2244 | 0.0204 | 0.0131 | -0.0226 |
| 9-3 | -0.0812 | 0.0856 | 0.2835 | 0.0931 | -0.2502 | 0.0343 | 0.2244 | 0.0204 | 0.0131 | -0.0226 |
| 10-1 | -0.1205 | 0.0290 | 0.2406 | -0.2351 | -0.0913 | 0.2094 | -0.2176 | -0.0155 | -0.0098 | 0.0253 |
| 10-2 | -0.1205 | 0.0290 | 0.2406 | -0.2351 | -0.0913 | 0.2094 | -0.2176 | -0.0155 | -0.0098 | 0.0253 |
| 10-3 | -0.1205 | 0.0290 | 0.2406 | -0.2351 | -0.0913 | 0.2094 | -0.2176 | -0.0155 | -0.0098 | 0.0253 |
| 11-1 | -0.1649 | -0.0181 | 0.0754 | -0.3087 | 0.2188 | -0.1535 | -0.0056 | -0.0028 | -0.0029 | -0.0034 |
| 11-2 | -0.1649 | -0.0181 | 0.0754 | -0.3087 | 0.2188 | -0.1535 | -0.0056 | -0.0028 | -0.0029 | -0.0034 |
| 11-3 | -0.1649 | -0.0181 | 0.0754 | -0.3087 | 0.2188 | -0.1535 | -0.0056 | -0.0028 | -0.0029 | -0.0034 |
| 12-1 | -0.2344 | -0.0644 | -0.1598 | -0.0467 | 0.0988 | -0.1840 | 0.2222 | 0.0193 | 0.0124 | -0.0211 |
| 12-2 | -0.2344 | -0.0644 | -0.1598 | -0.0467 | 0.0988 | -0.1840 | 0.2222 | 0.0193 | 0.0124 | -0.0211 |
| 12-3 | -0.2344 | -0.0644 | -0.1598 | -0.0467 | 0.0988 | -0.1840 | 0.2222 | 0.0193 | 0.0124 | -0.0211 |
| 13-1 | -0.1906 | -0.0597 | -0.1936 | 0.1280 | -0.1145 | 0.0971 | -0.0790 | 0.0521 | 0.1082 | 0.3964 |
| 13-2 | -0.1906 | -0.0597 | -0.1936 | 0.1280 | -0.1145 | 0.0971 | -0.0790 | 0.0521 | 0.1082 | 0.3964 |
| 13-3 | -0.1906 | -0.0597 | -0.1936 | 0.1280 | -0.1145 | 0.0971 | -0.0790 | 0.0521 | 0.1082 | 0.3964 |
| 14-1 | -0.1896 | -0.0594 | -0.1927 | 0.1284 | -0.1158 | 0.1014 | -0.1384 | -0.0694 | -0.1204 | -0.3756 |
| 14-2 | -0.1896 | -0.0594 | -0.1927 | 0.1284 | -0.1158 | 0.1014 | -0.1384 | -0.0694 | -0.1204 | -0.3756 |
| 14-3 | -0.1896 | -0.0594 | -0.1927 | 0.1284 | -0.1158 | 0.1014 | -0.1384 | -0.0694 | -0.1204 | -0.3756 |

Notice: PCNM, Principal Coordinates of Neighborhood Matrices.

**Table S7. Forward selection results of environment and spatial (PCNM vector) variable for meio- and microeukaryotic zooplankton, and zoobenthos communities**

| Taxa | Environment variable | | | PCNM vector | | |
|---|---|---|---|---|---|---|
| | Two seasons | Summer | Winter | Two seasons | Summer | Winter |
| Meio- and microeukaryotes | T, EC, NO2-N, NH4-N, NO3-N, TP, Salanity, COD, pH, TN | NO2-N, NO3-N, EC, T, NH4-N, DO, pH | EC, NO2-N, pH, COD, TP | PCNM (No. 1, 2) | PCNM (No. 1, 2, 3, 4, 5, 6, 7, 10) | PCNM (No. 1, 2, 3, 4, 5, 6, 7) |
| Zooplankton | T, EC, NH4-N, NO2-N, TP, Salinity, COD | NO2-N, T, EC, NH4-N, NO3-N | EC, NO2-N, DO, COD | PCNM (No. 1, 2) | PCNM (No. 1, 2, 3, 4, 5, 6, 7, 10) | PCNM (No. 1, 2, 3, 4, 5, 6, 7) |
| Zoobenthos | T, EC, NO2-N, NH4-N, TP, NO3-N, Salinity, COD, DO | NO2-N, T, EC, DO | EC, NO2-N, pH, Salinity, DO, COD | PCNM (No. 1, 2, 3) | PCNM (No. 1, 2, 3, 4, 5, 6, 7, 10) | PCNM (No. 1, 2, 3, 4, 5, 6, 7, 8) |

Notice: T, water temperature; DO, dissolved oxygen; COD, chemical oxygen demand; EC, electrical conductivity; TDS, total dissolved solids; $NO_2$-N, nitrite nitrogen; $NO_3$-N, nitrate nitrogen; $NH_4$-N, ammonium nitrogen; TN, total nitrogen; TP, total phosphorus. PCNM, Principal Coordinates of Neighborhood Matrices.

**Table S8. Species identification in this study.**

Please see the Excel file **Table S8**.

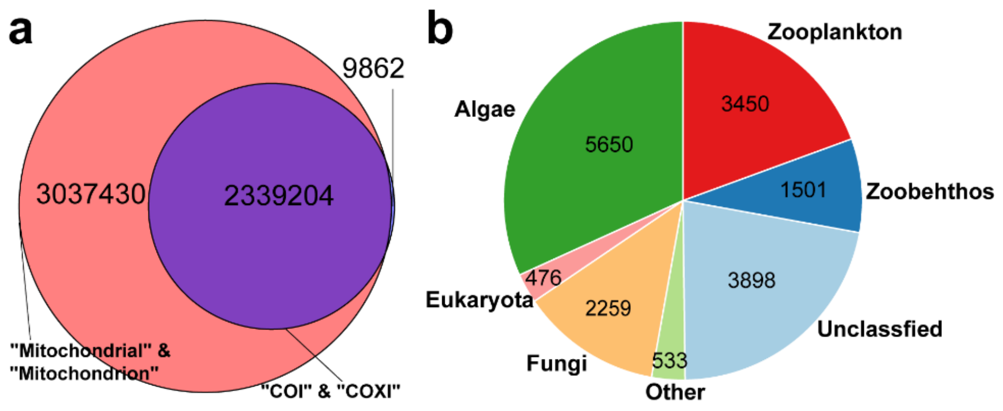


**Fig. S1.** Composition of the Mito-COI reference database and ASVs. **(a)** Composition of Mito-COI reference database based on four keywords searched in the NCBI. The number indicates the sequence number. **(b)** Composition of ASVs for different categories of taxa. The number indicates the ASV number of each taxon. Eukaryota, unclassified eukaryotes; Other, other identified species not classified as algae, zooplankton, zoobenthos, and fungi; Unclassified, unidentified ASVs.

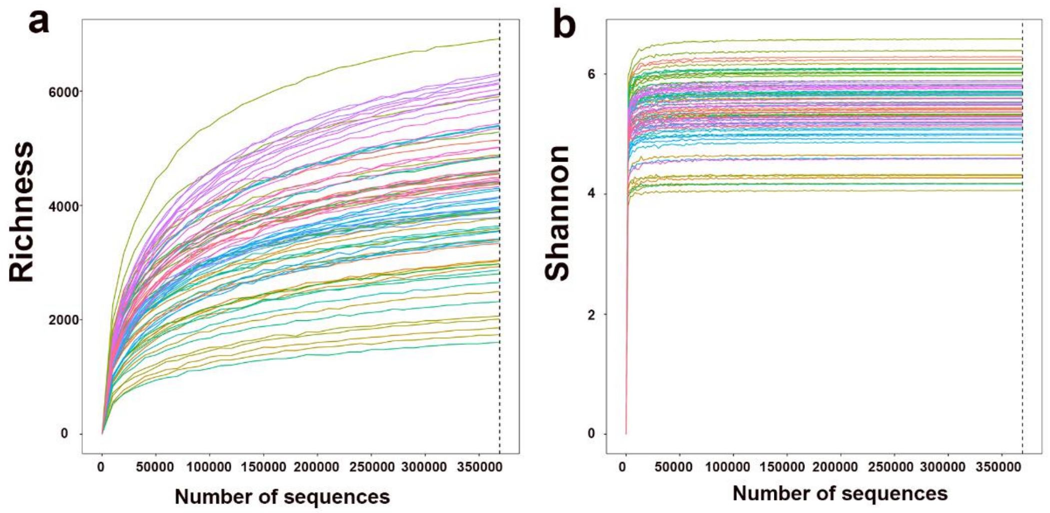


**Fig. S2.** Rarefaction curves of various samples. **(a)** Rarefaction curves of ASV richness for 84 samples. **(b)** Rarefaction curves of the Shannon index for 84 samples.

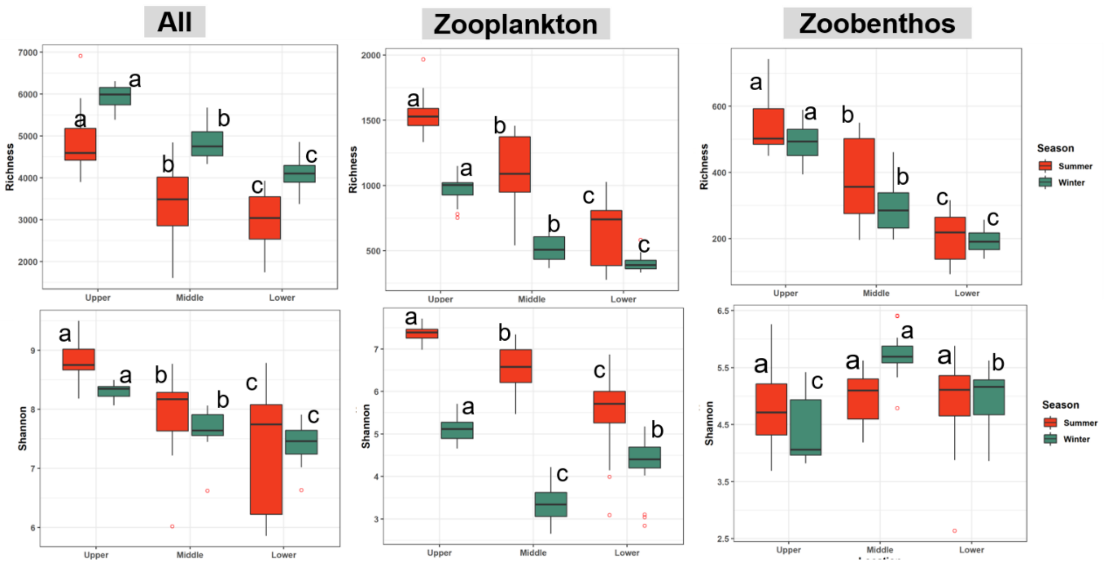


**Fig. S3.** Comparison of diversity indices for meio- and microeukaryotic, zooplankton, and zoobenthos communities among the three reaches in the summer and winter. Different letters above boxes indicate significant differences at the $p < 0.05$ level using analysis of one-way variance (ANOVA). All, all ASVs (meio- and micro-eukaryotic communities).

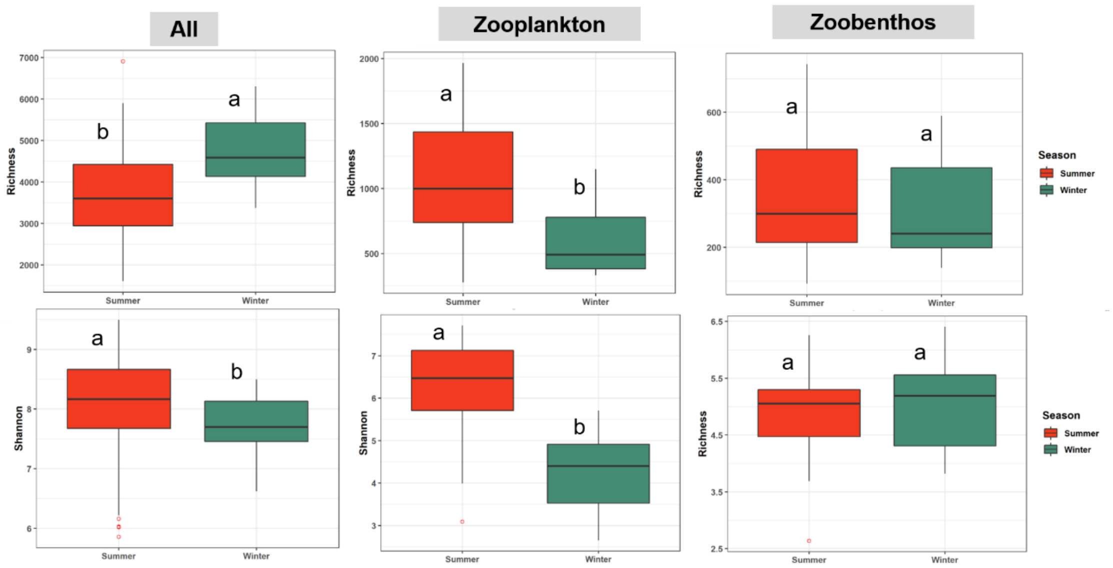


**Fig. S4.** Comparison of diversity indices for meio- and microeukaryotic, zooplankton, and zoobenthos communities between summer and winter. Different letters above boxes indicate significant differences at the $p < 0.05$ level using the Mann-Whitney *U* test. All, all ASVs (meio- and microeukaryotic community).

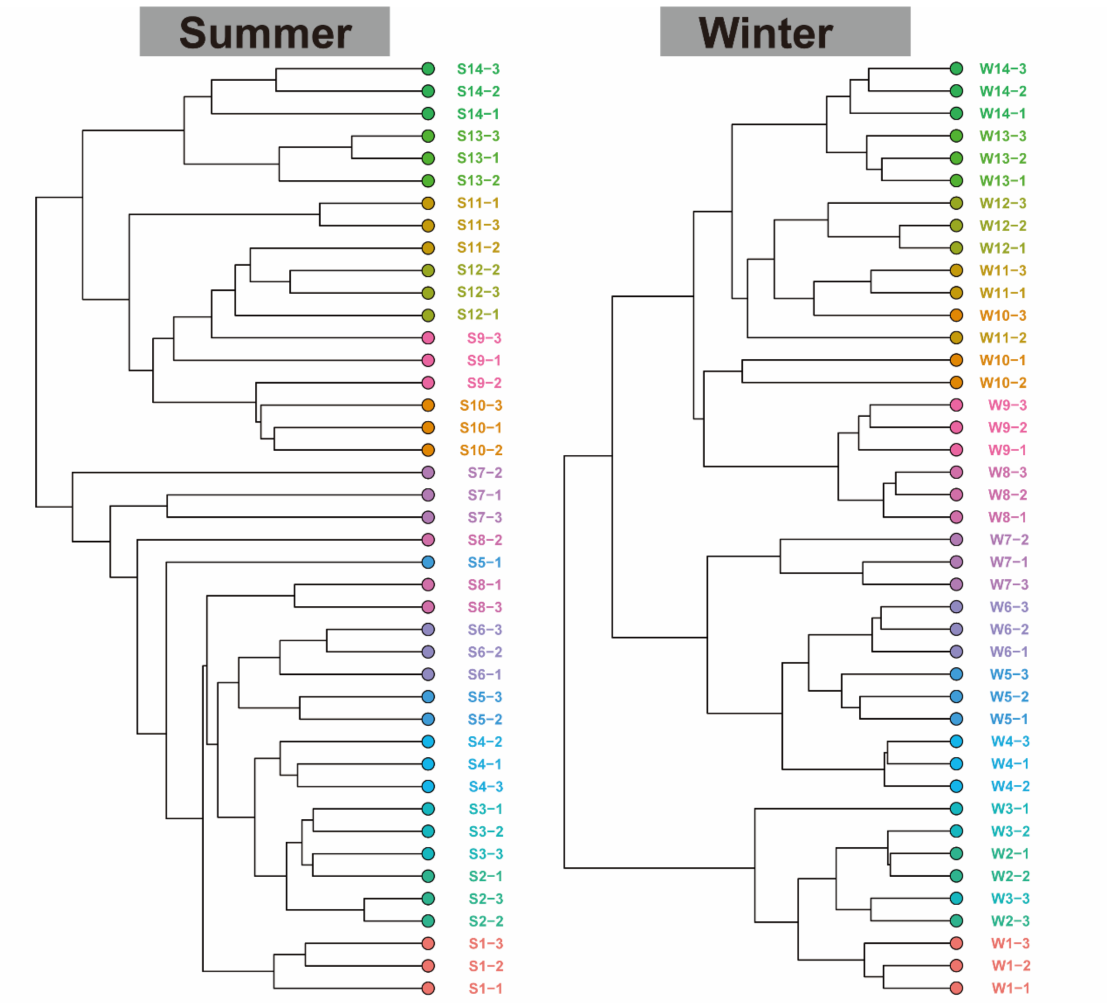


**Fig. S5.** The phylogenetic tree is based on the Bray-Curtis' similarity of meio- and microeukaryotes among 42 samples in summer and winter, respectively. Sample names are colored according to season (S and W) and sample sites (1-14) of sampling in three replicates (1-3) and details are provided in **Supplementary Table S1**.

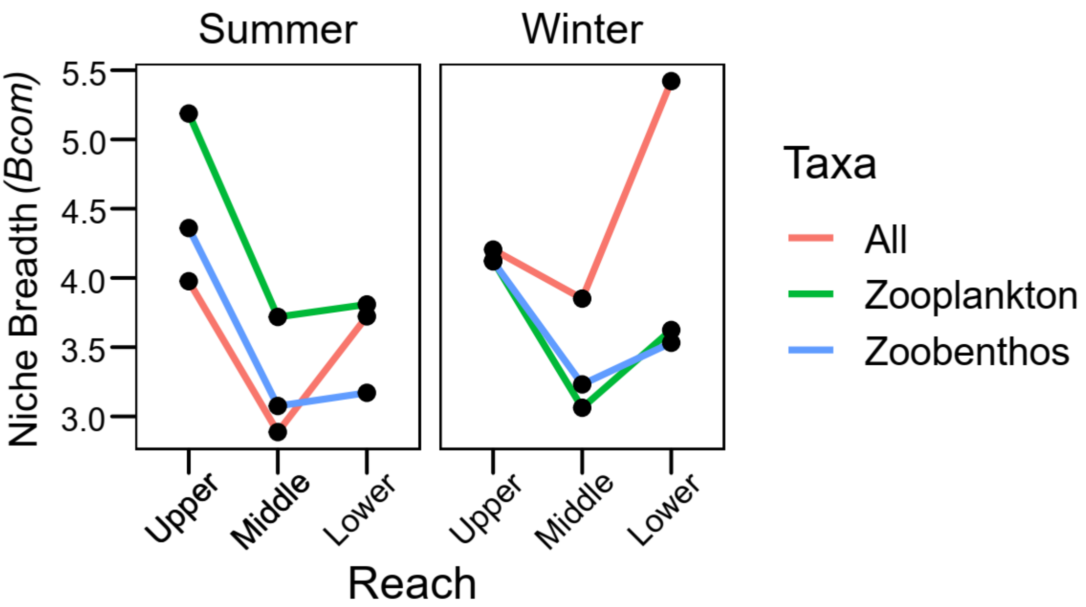


**Fig. S6. Mean habitat niche breadth (*Bcom*) for meio- and microeukaryotes, zooplankton, and zoobenthos at three reaches of TGR in summer and winter.** All, all ASVs (meio- and microeukaryotic community).
